# Targeted Metabolites and Transcriptome Analysis Uncover the Putative Role of Auxin in Floral Sex Determination in *Litchi chinensis* Sonn.

**DOI:** 10.3390/plants13182592

**Published:** 2024-09-16

**Authors:** Zhe Chen, Tingting Yan, Farhat Abbas, Mingchao Yang, Xianghe Wang, Hao Deng, Hongna Zhang, Fuchu Hu

**Affiliations:** 1 Institute of Tropical Fruit Trees, Hainan Academy of Agricultural Sciences/Key Laboratory of Genetic Resources Evaluation and Utilization of Tropical Fruits and Vegetables (Co-Construction by Ministry and Province), Ministry of Agriculture and Rural Affairs/Key Laboratory of Tropical Fruit Tree Biology of Hainan Province, Haikou 571100, China; chenzhe@hnaas.org.cn (Z.C.); yantt_hnaas@163.com (T.Y.); farhatmerani@yahoo.com (F.A.); yangmc_hnaas@163.com (M.Y.); wxh198@sina.com (X.W.); 2 Sanya Research Institute, Hainan Academy of Agricultural Sciences, Sanya 572025, China; 3 Institute of Agro-Products Processing and Design, Hainan Academy of Agricultural Sciences/Key Laboratory of Tropical Fruit and Vegetable Cold-Chain of Hainan Province, Haikou 571100, China; denghao@hnaas.org.cn; 4 Hainan Provincial Key Laboratory of Quality Control of Tropical Horticultural Crops, School of Tropical Agriculture and Forestry, Hainan University, Danzhou 571737, China; 994357@hainanu.edu.cn

**Keywords:** litchi, flower bud differentiation, sex of flower, crop production, auxin

## Abstract

Litchi exhibits a large number of flowers, many flowering batches, and an inconsistent ratio of male and female flowers, frequently leading to a low fruit-setting rate. Floral sexual differentiation is a crucial phase in perennial trees to ensure optimal fruit production. However, the mechanism behind floral differentiation remains unclear. The objective of the study was to identify the role of auxin in floral differentiation at the transcriptional level. The results showed that the ratio of female flowers treated with naphthalene acetic acid (NAA) was significantly lower than that of the control stage (M0/F0). The levels of endogenous auxin and auxin metabolites were measured in male and female flowers at different stages of development. It was found that the levels of IAA, IAA-Glu, IAA-Asp, and IAA-Ala were significantly higher in male flowers compared to female flowers. Next-generation sequencing and modeling were employed to perform an in-depth transcriptome analysis on all flower buds in litchi ‘Feizixiao’ cultivars (*Litchi chinensis* Sonn.). Plant hormones were found to exert a significant impact on the litchi flowering process and flower proliferation. Specifically, a majority of differentially expressed genes (DEGs) related to the auxin pathway were noticeably increased during male flower bud differentiation. The current findings will enhance our comprehension of the process and control mechanism of litchi floral sexual differentiation. It also offers a theoretical foundation for implementing strategies to regulate flowering and enhance fruit production in litchi cultivation.

## 1. Introduction

*Litchi chinensis* Sonn., a member of the Sapindaceae family, is a significant tropical and subtropical fruit tree with a cultivation history spanning over 2300 years, playing an integral role in the fruit industry economy of China [1]. Studies reveal that the flowering of litchi has always been an important factor in the high and relentless output of litchi. Litchi is distinguished by its abundant floral display, numerous flowering phases, and an unpredictable ratio of male and female flowers, frequently leading to lower fruit setting [2]. Therefore, the study of the regulatory mechanism of litchi flower bud sex differentiation can provide a theoretical foundation for the precise regulation of litchi flower sex, hence promoting fruit setting in production.

Sex determination in flowering plants is a multifaceted process involving complicated morphological, physiological, and biochemical mechanisms. It is crucial for adaptability, survival, and reproduction, and is influenced by several internal and external variables [3,4]. Various environmental factors, including temperature, light, mineral nutrition, and water, significantly influence the process of sex differentiation in flower buds of numerous plant species [5]. Most of the environmental regulation of floral differentiation is determined by epigenetic inheritance influenced by DNA modification and small RNA expression [6]. Furthermore, plant hormones are intricately linked to the process of sexual differentiation [7]. Research has demonstrated that gibberellin, auxin, cytokinin, ethylene, polyamine, and other plant hormones exert an influence on the process of sex differentiation in plants [8,9,10].

The flowering pattern of litchi is influenced by various elements, including a variety of traits, climatic conditions, endogenous hormones, and tree nutrition [11]. Previously, Abscisic acid was found to promote flowering via enhancing *LcAP1* expression in *Litchi chinensis* Sonn. Levels of endogenous hormones and endogenous polyamine during sexual differentiation of litchi flowers were studied. Relatively high levels of ABA would be beneficial to female differentiation, while low levels of IAA would be feasible for female flower development [12,13,14]. Litchi is a monoecious plant that produces determinate inflorescences on the current-season terminal shoots. Litchi flowers are grouped into three categories based on the development and function of their stamens and carpels: male flowers (type I), hermaphrodite functional female (type II), and hermaphrodite functional male flowers (type III). Type I flowers are devoid of ovules and possess functioning male characteristics (Male = M) [15]. Typically, there is a sequential pattern of blooming for three types of flowers: male, female, and male. The initial flowers to bloom are usually male flowers (type I) [16]. However, in certain years, the first flowers to bloom may be female, indicating that genes responsible for carpel development are activated and accelerated prematurely. Nevertheless, there is limited knowledge regarding the molecular foundation and regulatory mechanisms that govern the sexual differentiation of litchi flowers.

Floral bud sex differentiation serves as both a biological reality and an efficient technique for long-term plant development to prevent self-pollination. Floral sex distinction varies among different plant species. Hence, herein, we performed targeted metabolomics and transcriptomic analysis of litchi flower buds from the initiation of bud formation to the completion of flowering using next-generation sequencing. This allowed us to examine the histological, morphological, physiological, and molecular biological mechanisms involved in the sexual differentiation of litchi flower buds. The current study aims to uncover the process of floral sex differentiation, development, and its regulatory effects in litchi. Additionally, it seeks to elucidate the regulatory network involving metabolism, signal transduction, and gene expression during the floral sex differentiation stage. These findings will contribute to enhancing the theoretical understanding of floral sex differentiation in tropical fruit trees.

## 2. Materials and Methods

### 2.1. Plant Materials

*Litchi chinensis* ’Feizixiao’ (FZX) was planted in the innovation experimental orchard of the Hainan Academy of Agricultural Science, Haikou, China. Trees approximately five to six years old, exhibiting uniform height and vigor, were selected. We selected robust plants exhibiting consistent growth patterns. Flower buds from both female and male plants were gathered at three distinct stages of development. All these samples, each with three biological replicas, were collected simultaneously to mitigate any variations in gene expression triggered by circadian rhythm factors. The samples were collected from three different directions of a tree, immersed in liquid nitrogen, and kept at −80 °C for RNA extraction.

### 2.2. Exogenous Hormone Application

Exogenous hormone application was performed in the following work based on prior research [17]. When the litchi flower clusters reach a certain level of maturity, with plump and fully formed flower grains and the male flowers still closed, it is recommended to apply a spray of Naphthalene acetic acid (NAA) solution to the clusters. For the control group (CK), spray with water. Prior to spraying, attach three tags to each tree for observation and investigation. Make sure to closely monitor the number of male and female flowers every other day throughout the entire flowering period after applying the spray. We recorded the duration of flowering, the quantity of male and female flowers during flowering, and their ratio. NAA 67 mg/L was sprayed once on flower panicles at the bud stage to examine their impact on floral dynamics. HPLC-MS was used to determine the concentrations of IAA during different developmental stages [18].

### 2.3. Morphological and Structural Observation

Samples were gathered from late February to mid-late March, and the flower development process was divided into three phases. The process of litchi flower bud differentiation typically involves several stages: the maturation of the last autumn shoot, flower induction, flower initiation (referred to as the ‘white point stage’), inflorescence development, flower development, panicle formation, and finally, flowering. It seems that the term ‘bud break’ may refer to the ‘white point stage’, suggesting that the application should take place approximately 30 days after bud break. The morphological and structural observations were performed using a stereoscope.

### 2.4. RNA Extraction, cDNA Library Construction, and Sequencing

Total RNA was isolated using the Quick RNA Isolation Kit and treated with DNase I (TaKaRa, Shiga, Japan) to remove any genomic DNA contamination. The purity of the RNA samples was determined using the NanoPhotometer^®^spectrophotometer (IMPLEN, Westlake Village, CA, USA), and RNA integrity was evaluated using the RNA Nano 6000 Assay Kit of the Bioanalyzer 2100 system (Agilent Technologies, Santa Clara, CA, USA). The index-coded samples were clustered using a cBot Cluster Generation System with TruSeq PE Cluster Kit v3-cBot-HS (Illumina, San Diego, CA, USA) following the instructions provided by the vendor. After cluster generation, the library preparations were sequenced on an Illumina Novaseq platform and 150 bp paired-end reads were generated. The above process was performed at the Shanghai biotree biotech CO., LTD. The size and concentration of the library were evaluated using Qubit 2.0 and Agilent 2100. Using a Hi-Seq 2500 sequencing machine, pair-end (150 PE) Illumina high-throughput sequencing was conducted. The RNA-Seq libraries were prepared for three replicates. The transcriptome raw data have been deposited in the NCBI database with BioProject ID: PRJNA1147420.

**Gene Functional Annotation and Expression Level Analysis**

From the raw reads, the low-quality reads and adopters were filtered. The clean reads were de novo assembled into contigs with an optimized k-mer length = 25 and group pair distance = 300 using the Trinity program 2. The unigene functions were predicted via BLAST against the NCBI non-redundant protein (Nr), NCBI nucleotide sequences (Nt), and Swiss-Prot databases (*E*-value of 10^−5^). The resulting datasets were validated to the Protein family (Pfam) database [19] with HMMER (*E*-value 10^−10^). Unigene sequences were aligned against the Gene Ontology (GO) [20] and Kyoto Encyclopedia of Genes and Genomes (KEGG) [21] databases. The expression levels of genes in the samples were measured via the FPKM procedure. Based on gene read count data, DEGseq software (1.32.0 version) was employed to identify differentially expressed genes (DEGs). The following criteria were established for screening DEGs: | log2 (fold change) | ≥ 1 and false discovery rate (FDR) < 0.05 using the litchi genome [1] as a reference genome. The detailed methodology provided is included in the Supplementary Materials.

### 2.5. Quantitative Analysis of Endogenous Auxin by LC-MS/MS

Chemicals and reagents: HPLC grade acetonitrile (ACN) and methanol (MeOH) were purchased from Merck (Darmstadt, Germany). MilliQ water (Millipore, Bradford, PA, USA) was used in all experiments. All the standards were purchased from Olchemim Ltd. (Olomouc, Czech Republic) and isoReag (Shanghai, China). Acetic acid and formic acid were bought from Sigma-Aldrich (St Louis, MO, USA). The stock solutions of standards were prepared at the concentration of 1 mg/mL in MeOH. All stock solutions were stored at −20 °C. The stock solutions were diluted with MeOH before analysis.

Sample preparation and extraction: Fresh plant samples were harvested, immediately frozen in liquid nitrogen, ground into powder (30 Hz, 1 min), and stored at −80 °C until needed. About 50 mg of plant sample was weighed into a 2 mL plastic microtube, frozen in liquid nitrogen, and dissolved in 1 mL methanol/water/formic acid (15:4:1, *v*/*v*/*v*). About 10 μL internal standard mixed solution (100 ng/mL) was added into the extract as internal standards (IS) for the quantification. The mixture was vortexed for 10 min followed by centrifugation for 5 min (12,000 r/min, and 4 °C). The supernatant was transferred to clean plastic microtubes, followed by evaporation to dryness, dissolved in 100 μL 80% methanol (*v*/*v*), and filtered through a 0.22 μm membrane filter for further LC-MS/MS analysis [22,23,24].

UPLC Conditions: The sample extracts were analyzed using a UPLC-ESI-MS/MS system (UPLC ExionLC™ AD https://sciex.com.cn/ (accessed on 23 April 2023); MS’ Applied Biosystems 6500 Triple Quadrupole, https://sciex.com.cn/). The analytical conditions were as follows, LC: column, Waters ACQUITY UPLC HSS T3 C18 (100 mm × 2.1 mm i.d. 1.8 µm); solvent system, water with 0.04% acetic acid (A), acetonitrile with 0.04% acetic acid (B); gradient program, started at 5% B (0–1 min), increased to 95% B (1–8 min), 95% B (8–9 min), finally ramped back to 5% B (9.1–12 min); flow rate, 0.35 mL/min; temperature, 40 °C; injection volume: 2 μL [25,26].

ESI-MS/MS Conditions: Linear ion trap (LIT) and triple quadrupole (QQQ) scans were acquired on a triple quadrupole-linear ion trap mass spectrometer (QTRAP), QTRAP^®^ 6500+ LC-MS/MS System, equipped with an ESI Turbo IonSpray interface, operating in both positive and negative ion mode and controlled by Analyst 1.6.3 software (Sciex). The ESI source operation parameters were as follows: ion source, ESI+/−; source temperature 550 °C; ion spray voltage (IS) 5500 V˄ Positive˅, −4500 V (Negative); curtain gas (CUR) was set at 35 psi. Phytohormones were analyzed using scheduled multiple reaction monitoring (MRM). Data acquisitions were performed using Analyst 1.6.3 software (Sciex). Multiquant 3.0.3 software (Sciex) was used to quantify all metabolites. Mass spectrometer parameters including the declustering potentials (DP) and collision energies (CE) for individual MRM transitions were conducted with further DP and CE optimization. A specific set of MRM transitions was monitored for each period according to the metabolites eluted within this period [18,27].

### 2.6. Data Analysis

The raw data, in the form of fastq format raw readings, underwent initial processing using custom Perl scripts. During this stage, the data were purified by eliminating reads that contained adapters, poly-N sequences, and reads with low quality from the raw data. Reference genome and gene model annotation files were obtained from the genome website (www.sapindaceae.org; accessed on 23 April 2023). The index of the reference genome was built using Hisat2 v2.0.5 and paired-end clean reads were aligned to the reference genome using Hisat2 v2.0.5. Differential expression analysis of two conditions/groups (two biological replicates per condition) was performed using the DESeq2 R package (1.16.1). Genes with an adjusted *p*-value < 0.05 found by DESeq2 were assigned as differentially expressed.

### 2.7. Validation of DEGs by qRT-PCR

The expression pattern of selected DEGs was validated by qPCR as explained previously [28]. Briefly, the cDNA was synthesized from RNA samples using the PrimeScript RT Reagent Kit with gDNA Eraser (Perfect Real Time) (TaKaRa Bio Inc., Kusatsu, Shiga, Japan), and qRT-PCR was performed using TB Green Premix Ex Taq II (TIi RNaseH Plus) (TaKaRa) on a Bio-Rad CFX96TM Real-Time System (Bio-Rad, Hercules, CA, USA) as explained earlier [29]. Relative expression was calculated using the delta CT method [30]. Actin was used as an endogenous control. All reactions were performed with three biological and technical replications. Primers are listed in Table S4.

### 2.8. Statistical Analysis

Statistical analyses were performed with SPSS 19.0 software (SPSS, Chicago, IL, USA). One-way analysis of variance (ANOVA) was used to evaluate the difference in each sample. Heatmap diagrams were performed using R4.2.1 software with heatmap methods. Significant correlations between qRT-PCR and transcriptome data were analyzed with SPSS software using Pearson’s correlation as the statistical metric. Significant correlations were considered only when an adjusted *p* value was lower than 0.05.

### 2.9. Availability of Supporting Data

The raw data for the digital gene expression analysis were also deposited in the NCBI with Submission ID: SUB14661989 and BioProject ID: PRJNA1147420.

## 3. Results

### 3.1. Exogenous Auxin Application Promotes Male Flower Development

**Figure 1:**
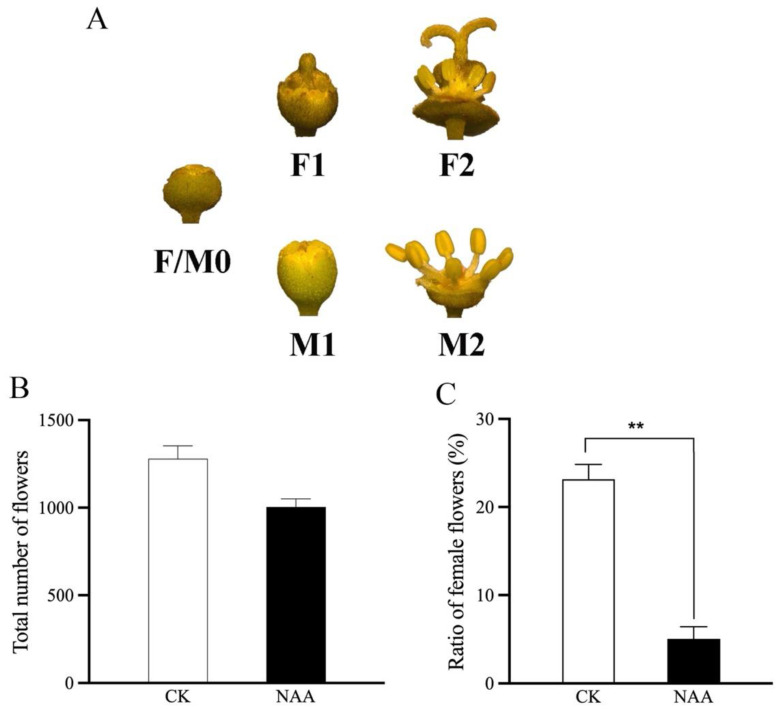
Morphology of litchi flower development and effect of NAA on the ratio of flowers. (**A**) Pictorial observation of litchi floral bud development. Impact of exogenous NAA application on (**B**) total number of flowers and (**C**) ratio of female flowers. CK denotes control. Vertical bars denote standard error of mean of 3 biological replicates; differences at significance of *p* < 0.01 are denoted by double asterisks (**).

The differentiation of inflorescence and the development of floral organs in litchi occurred simultaneously between late December and mid-late March. The process was divided into three distinct stages: sepal formation and amphoteric primordium development stage (F0/M0), pistil and stamen growth stage (F1/M1), and fully mature female and male flowering stage (F2/M2) (Figure 1A). During the F0/M0 stage, the calyx undergoes rapid growth and becomes densely covered with fine hair-like structures, effectively concealing the other internal organs of the flower (Figure 1A). The bud exhibits a green coloration at its base and a white coloration at its apex, while being covered in a layer of fuzz. In the F1/M1 stage, the buds continue to enlarge, and the calyx stays tightly packed; the stamens form distinct divisions, classifying them into four halves; the white hairs on the surface of the pistil increase and get longer. The F2/M2 stage is the optimal period for pollination. During this stage, the buds continue to expand, the calyx splits open, the stamens extend straight out, the anthers are well-developed, the filaments are elongated, and the stigma is cracked at a 180-degree angle.

To investigate whether auxin plays a role in floral sexual differentiation in litchi, we performed exogenous auxin (1-Naphthaleneacetic acid, NAA) application on the litchi panicles before flowering, and then analyzed the floral sex phenotypes. The findings indicated that there was no notable disparity between the NAA treatment and the control group (M0/F0) in terms of the overall number of flowers (Figure 1B,C). However, the ratio of female flowers treated with NAA was significantly reduced compared to the control group (Figure 1C). These findings suggest that auxin exerts a suppressive effect on the growth and development of female flowers.

### 3.2. Endogenous IAA Contents Were Up-Regulated during Male Flower Development

**Figure 2:**
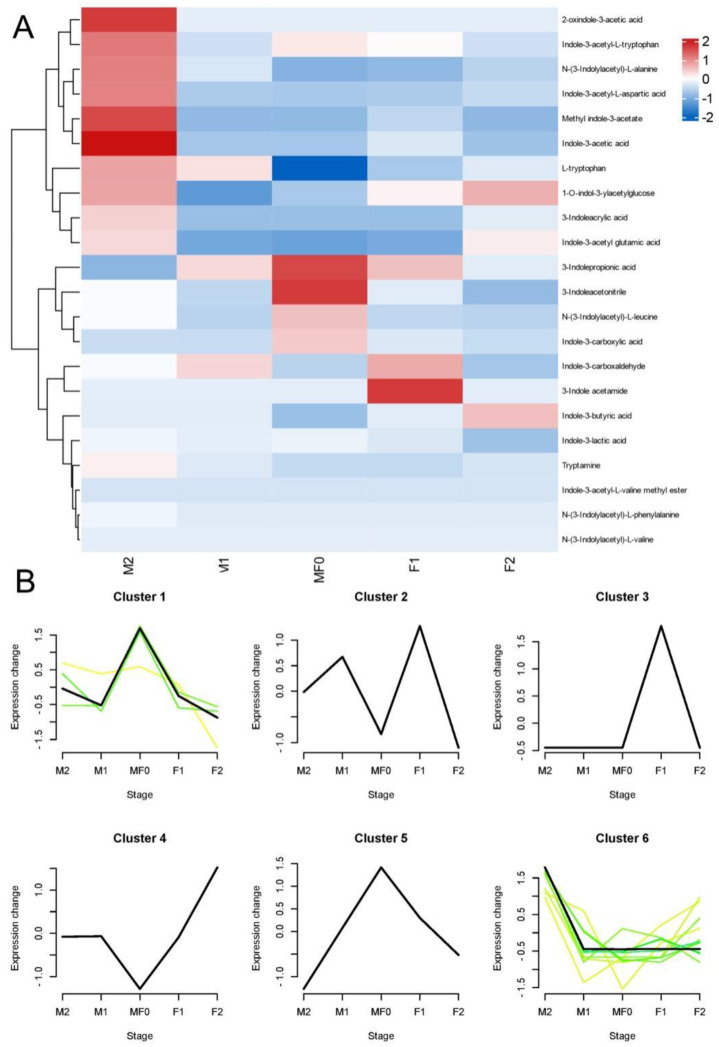
Expression analysis of auxin-related metabolites measured in litchi flowers during different developmental stages. (**A**) Heatmap and (**B**) K-mean cluster analysis of auxin-related metabolites detected during flower bud development. Data are denoted as the SEM of three biological replicates.

To determine the involvement of auxin in the mechanism of floral sex differentiation in litchi, we measured the endogenous auxin content and auxin metabolites at different developmental phases of male and female flowers. Twenty-six endogenous auxin-related metabolites were detected, including IAA (Indole-3-acetic acid), IPA (3-Indolepropionic acid), TRP (L-tryptophan), IAA-Glu (Indole-3-acetyl glutamic acid), IAA-Asp (Indole-3-acetyl-L-aspartic acid), IAA-Ala (N-(3-Indolylacetyl)-L-alanine), and several others (Figure 2: Supplementary Table S1). Figure 2A depicts the amount of auxin measured during litchi flower bud development in a heatmap. The data showed that the majority of auxins were upregulated during litchi male flower bud development compared to control (M0/F0).

The k-means cluster analysis categorized the 26 identified auxin metabolites into 6 different clusters, as shown in Figure 2B. Cluster 1 and Cluster 5 exhibited comparable patterns with elevated levels of auxins during the M0/F0 phases, and Cluster 2 during the M1 and F1 stages. In contrast, Cluster 3 displayed notably greater levels of auxin metabolites exclusively during the F1 stage. Similarly, in Cluster 4, the levels of auxin metabolites were lower at the M0/F0 stage and steadily increased as the female flowers developed. In Cluster 6, the levels of auxin metabolites were higher during the M2 stage compared to previous phases, which remained comparatively stable.

**Figure 3:**
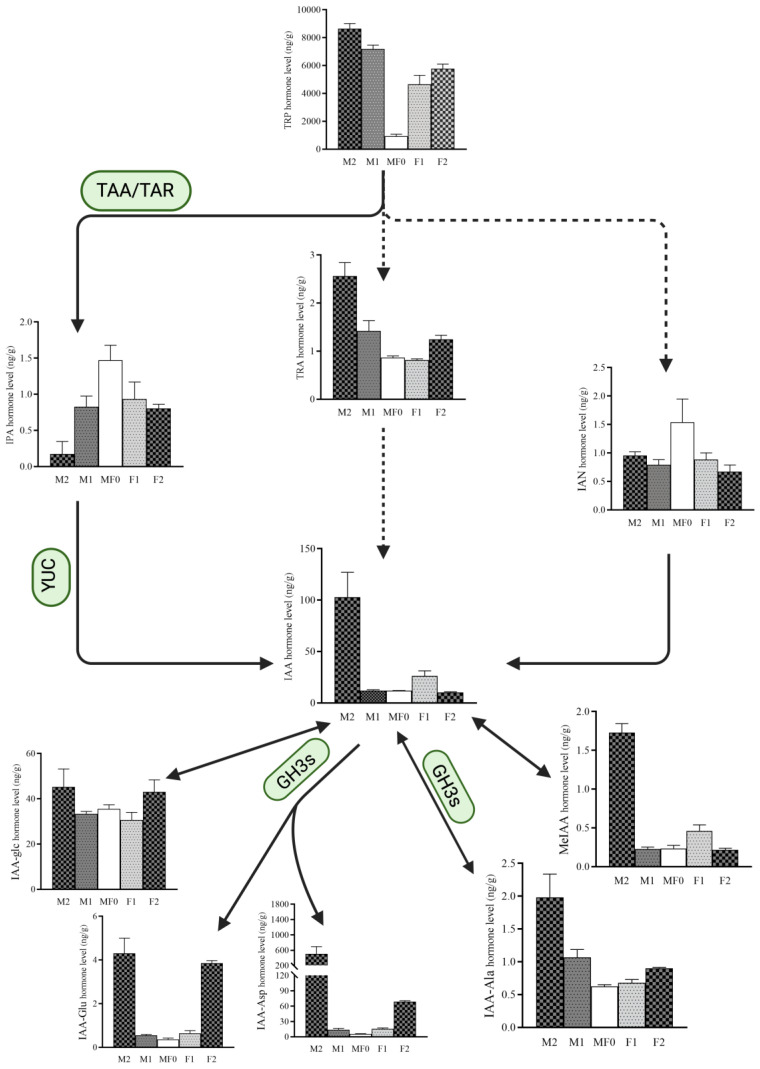
Endogenous content of auxins during different phases of male and female flower development. The contents were measured by LC-MS/MS. The data are denoted by SEM of three biological replicates. IAA; Indole-3-acetic acid, IPA; 3-Indolepropionic acid, TRP; L-tryptophan, IAA-Glu; Indole-3-acetyl glutamic acid, IAA-Asp; Indole-3-acetyl-L-aspartic acid, IAA-Ala; N-(3-Indolylacetyl)-L-alanine.

The content of tryptophan and TRA was substantially higher in male flowers, especially at the fully mature stage compared to control and female flowers. Likewise, the content of IAA, IAA-Glu, IAA-Asp, and IAA-Ala were substantially higher in the male flowers compared to the female flowers, indicating that the elevated level of these metabolites during the later stages of male flower development plays a critical role in the differentiation and overall development of male flowers. Interestingly, the content of IPA, the IAA precursor of the TRP-dependent pathway, displayed was higher in the M0/F0 stage compared to male or female flowers, which is contrary to the pattern of IAA content (Figure 3). However, the IAOx-dependent IAA biosynthesis pathway showed little contribution to the biosynthesis of IAA during litchi floral development. The data suggest that the TRP-dependent IAA biosynthesis pathway is primarily involved in the development of male flowers, with YUCs playing a crucial role in catalyzing IPA into IAA.

### 3.3. Transcriptome Analysis of the Flower Bud Development State

We conducted a comprehensive analysis of transcriptional changes occurring during litchi flower development using next-generation sequencing (RNA-seq) from the bud initiation to the fully open flowering stage. RNA-Seq libraries were prepared for three replicates and for all five conditions. Detailed information on RNA-seq data is enlisted in Supplementary Tables S2 and S3. Briefly, the data yielded 5.68–7.04 GB clean bases with an error rate of less than 0.03%, Q20 and Q30 values of around 98% and 93%, respectively, and a GC value of 44% (Supplementary Table S1). Additionally, the total mapped reads were around 90%, and the unique mapped reads were 84% (Figure S1, Supplementary Table S3). The principal component analysis (PCA) and correlation analysis revealed a strong association among the sampling data (Figure S2). In short, the data indicated that sequences were of higher grade.

### 3.4. Analysis of DEGs Specifically Expressed at the Floral Sexual Differentiation Stage

Fast and accurate alignment of clean reads with the reference genome was performed using HISAT2 software v2.2.0. The analysis resulted in a total of 33,657 unigenes after removing redundancies with CD-hit. Additionally, StringTie software v2.1.4 successfully assembled 1761 novel genes. We conducted GO functional annotation and KEGG pathway analysis of genes. The GO functional annotation revealed that 12,944 genes were categorized into 38 functional branches, including cell components, biological processes, and molecular functions. Moreover, 6377 genes showed enrichment in 140 KEGG pathways.

**Figure 4:**
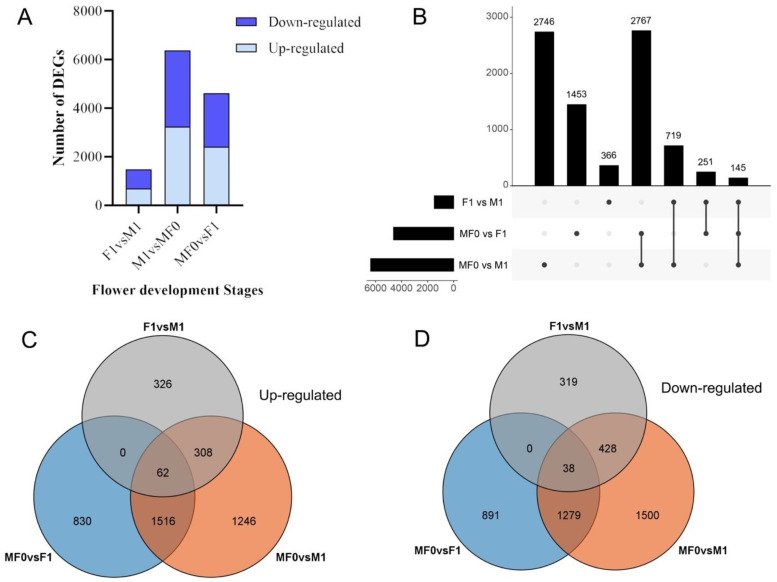
Auxin substantially influences the expression of genes during flower bud development. (**A**) Total number of DEGs. (**B**) UpSet R plot indicating the number of unique DEGs detected during different litchi flower development. Number of upregulated (**C**) and downregulated (**D**) DEGs denoted in Venn diagram.

The transcriptome analysis of female and male flower bud development stages was analyzed to investigate the impact of sex differences. Given the significance of the DEGs between the F1/M1 stages in floral sex determination, our main focus will be on exploring their integral role in this process. A total of 33,658 DEGs were detected, with lengths varying from 36 to 24,288 base pairs (Figure 4). In the M0/F0 vs. F1 stage, M0/F0 vs. M1 stage, and F1 vs. M1 stage, 2408, 3245, and 696 genes were upregulated, while 2208, 3132, and 785 were downregulated, respectively (Figure 4A). The UpSet plot shows that there were 2746, 1453, and 366 DEGs between the M0/F0 vs. M1 stage, M0/F0 vs. F1 stage, and F1 vs. M1 stage, respectively (Figure 4B). Furthermore, 2767, 719, and 251 DEGs were detected between M0/F0 vs. M1 and M0/F0 vs. F1 stages, M0/F0 vs. M1 and F1 vs. M1 stages, and M0/F0 vs. F1 and F1 vs. M1 stages, respectively. Furthermore, 145 DEGs were identified when all stages were compared.

A total of 326 were upregulated and 319 were downregulated between the F1 vs. M1 stages (Figure 4C,D). In the comparison between M0/F0 and F1 stages, there were 830 upregulated and 891 downregulated DEGs. Between the M0/F0 vs. M1 stages, 1246 DEGs were upregulated while 1500 DEGs were downregulated. Remarkably, a total of 62 DEGs were identified across all stages, exhibiting upregulation, whereas 38 DEGs were downregulated.

**Figure 5:**
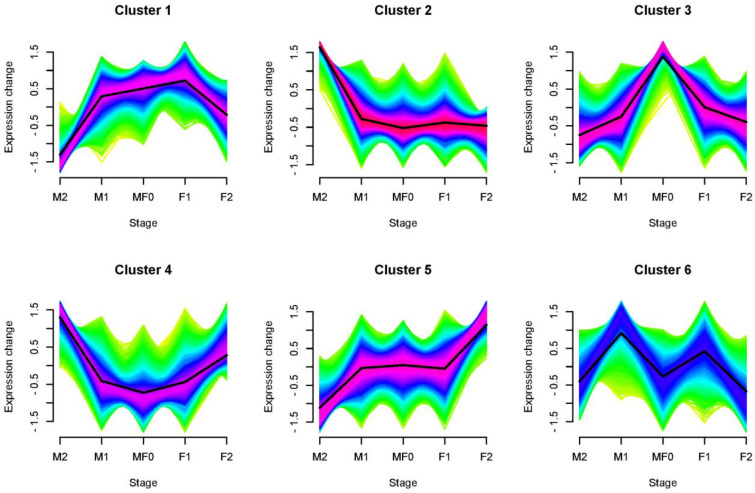
K-means Cluster analysis of DEGs obtained from the litchi floral bud development transcriptome dataset. The black line in the cluster represents the average expression pattern of DEGs.

To further investigate the expression pattern of DEGs, we used Cluster analysis using the RNA-seq dataset (Figure 5). The data indicated that the DEGs can be classified into six separate clusters. The DEGs in Cluster 1 showed greater expression levels at the M1, M0/F0, and F1 stages. In Cluster 2, the DEGs had higher expression levels at the M2 stage. Cluster 3 exhibited DEGs with higher expression levels at the M/F0 stage, while Cluster 4 showed DEGs with higher expression levels at the M2 and F2 stages. Cluster 5 displays DEGs with a higher expression level in the F2 stage compared to previous stages. In contrast, Cluster 6 shows higher expression levels at the M1 and F1 stages. Simply put, the data suggest that the expression of DEGs during a specific stage may be linked to their function in a particular development process.

### 3.5. GO and KEGG Annotation and Enrichment Analysis of DEGs

**Figure 6:**
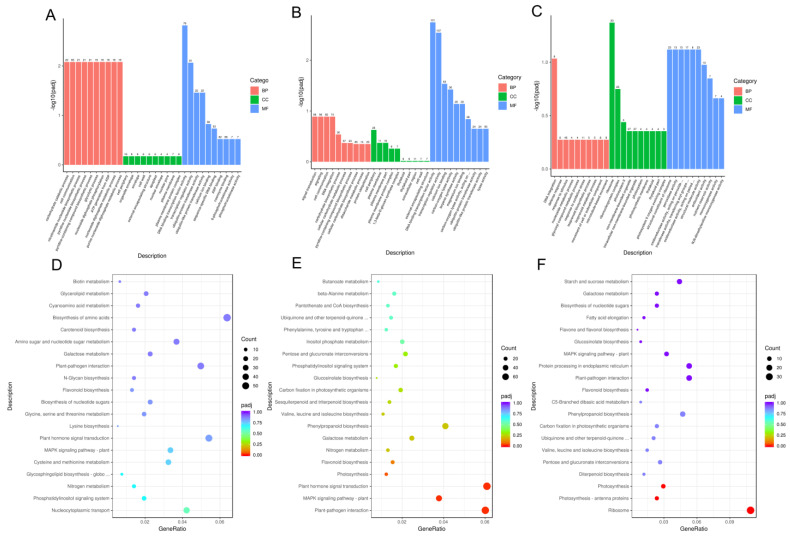
GO and KEGG analysis of DEGs during litchi floral bud development. GO classification among the (**A**) M0/F0 vs. F1, (**B**) M0/F0 vs. M1, and (**C**) F1 vs. M1 comparisons. MF, CC, and MF are denoted in red, green, and blue colors, respectively. BP; biological process, MF; molecular function, CC; cellular component. KEGG analysis of DEGs among the (**D**) M0/F0 vs. F1, (**E**) M0/F0 vs. M1, and (**F**) F1 vs. M1 comparisons.

**Figure 7:**
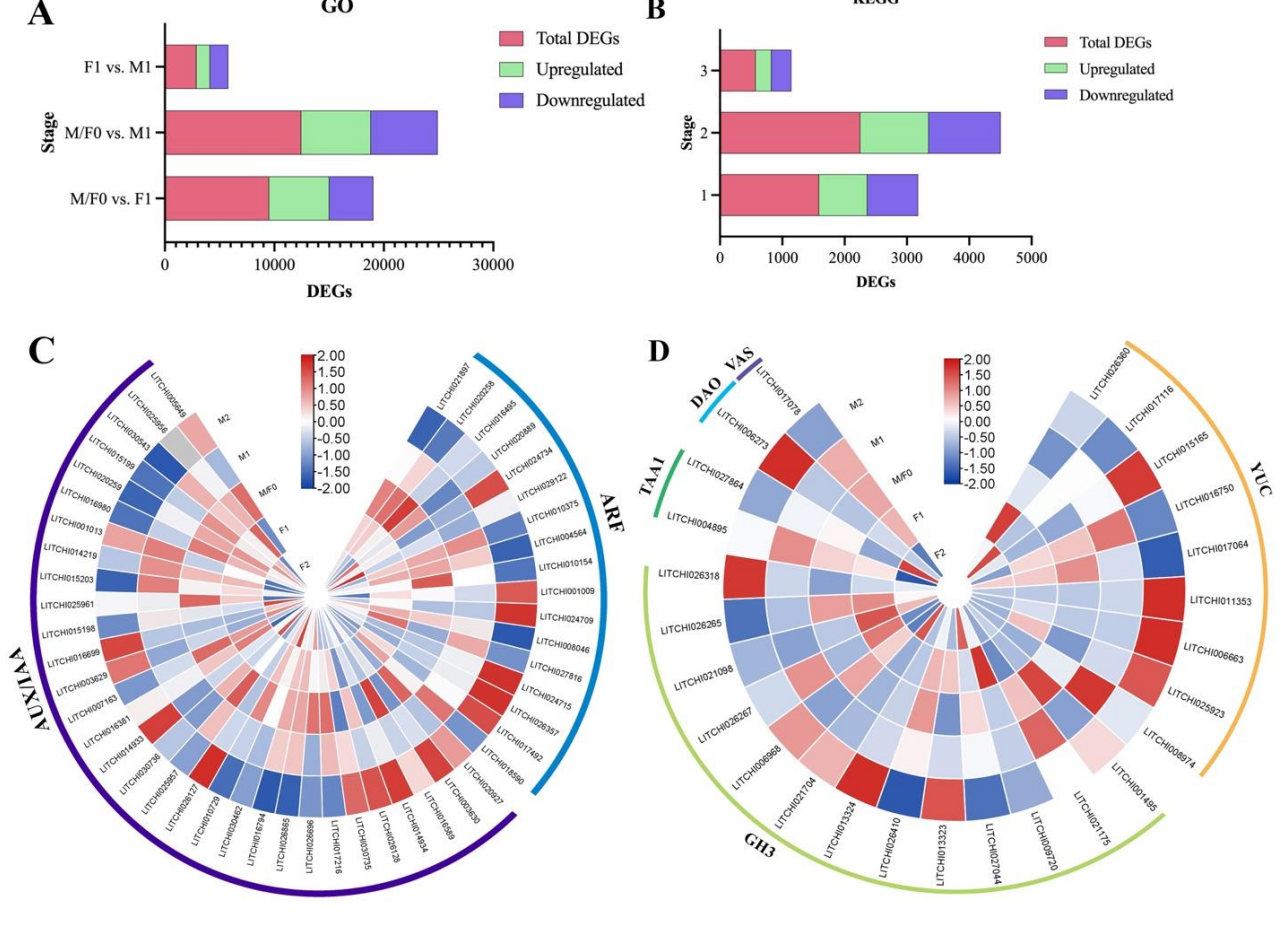
A depiction of DEGs found in the GO and KEGG databases, as well as DEGs related to auxin metabolism and signaling. (**A**) The DEGs found in GO and (**B**) KEGG pathways, including both upregulated and downregulated genes. (**C**) Heatmap showing the expression levels of auxin biosynthesis and (**D**) signaling-related genes identified in DEGs. The color scheme used in this study represents different levels of gene expression. Blue represents low expression, white indicates no specific pattern of expression, and red indicates high expression.

The function of DEGs was evaluated by employing the GO-based enrichment annotation. The GO enrichment annotation grouped the DEGs into three categories: biological process (BP), molecular function (MF), and cellular component (CC) (Figure 6A–C). The biological process had the highest proportion of DEGs between M/F0 and F1, followed by molecular function and cellular component. A total of 9523 DEGs were annotated using gene ontology. Within the set of DEGs that have been annotated with Gene Ontology, two processes stand out as being particularly enriched: carbohydrate catabolic process (GO:0016052) and cell communication (GO:0007154). Likewise, the majority of genes in the molecular function category were mainly associated with DNA binding transcription factor activity (GO:0003700), transcription regulator activity (GO:0140110), and ubiquitin-protein transferase activity (GO:0004842). Notably, the majority of these DEGs were classified within the molecular function category, as shown in Figure 6B. The majority of the DEGs were assigned to DNA binding transcription factor activity (GO:0003700), transcription regulator activity (GO:0140110), calcium ion binding (GO:0005509), and carbon-oxygen lyase activity (GO:0016835). Furthermore, a total of 2894 DEGs were identified, with a majority of them being strongly assigned to the cellular component category (Figure 6C). The majority of the highly enriched DEGs were assigned to the ribosome (GO:0005840). A higher number of DEGs were upregulated between the M0/F0 and F1 stages, as well as between the M0/F0 and M1 stages (Figure 7A). Conversely, there was a lower number of upregulated DEGs in the comparison between females at stage F1 and males at stage M1.

The KEGG database was used to classify DEGs according to the pathways they were involved in or the roles they performed. Based on the KEGG pathway enrichment analysis of DEGs, three common pathways were identified in the M0/F0 vs. F1, M0/F0 vs. M1, and F1 vs. M1 comparisons (Figure 6D–F). The pathways that showed the highest level of enrichment were the “MAPK signaling pathway”, “plant-pathogen interaction”, and “Flavonoid biosynthesis” (Figure 6A–C). Moreover, three shared highly enriched pathways (“plant hormone signal transduction”, “MAPK signaling pathway”, and “plant-pathogen interaction”) that were observed between M0/F0 vs. F1, and M0/F0 vs. M1 (Figure 6D,E). Interestingly, these shared pathways were highly enriched between M0/F0 and F1 compared to M0/F0 and M1, our primary focus. Additionally, between F1 and M1, “Plant pathogen interaction”, “Protein processing in endoplasmic reticulum”, and “Phenylpropanoid biosynthesis” were among the highly upregulated KEGG pathway, while the “Ribosome” KEGG pathway was substantially downregulated (Figure 6F). Furthermore, comparing the libraries M0/F0 vs. F1, M0/F0 vs. M1, and F1 vs. M1 yielded 1589, 2252, and 573 DEGs, respectively (Figure 7B). Interestingly, the number of DEGs was higher across all stages in comparison to upregulated DEGs.

### 3.6. Transcription Factors Associated with Floral Bud Development

Multiple studies unveil that transcription factors (TFs) are an integral part of the floral bud development mechanism. In our analysis, we found a total of 231, 287, and 48 transcription factors (TFs) that were expressed differentially in the comparisons of M0/F0 vs. F1, M0/F0 vs. M1, and F1 vs. M1, respectively (Table 1). A total of 566 copies of transcription factors (TFs) were found. Among them, the AP2/ERF TFs had the highest number with 93 copies, followed by MYB with 53 copies, WRKY with 49 copies, bHLH with 47 copies, C2H2 with 45 copies, MYB-related with 41 copies, NAC with 37 copies, and bZIP with 32 copies. Across all stages, the majority of TFs were highly upregulated versus downregulated. Between M0/F0 and F1, 150 TFs were upregulated and 81 were downregulated, whereas M0/F0 and M1 had 174 TFs upregulated and 113 downregulated, respectively. Similarly, 32 TFs were upregulated and 16 downregulated between the F1 and M1 stages, respectively. Aside from these, numerous other TFs, such as AUX/IAA, ARF, Dof, GATA, YABBY, GRAS, HSF, MADS, SNF2, Tify, and several more were affected across these stages.

### 3.7. Key Hormone-Related DEGs Associated with Auxin Biosynthesis and Signalling

Auxin, along with other plant hormones, plays a crucial role in the flowering and development of litchi flowers. In order to gain a deeper understanding of the regulation of genes that are expressed at various phases of flower bud development, we performed an investigation into the expression of genes connected to plant hormone pathways using a heatmap. Herein, we found 74 DEGs related to auxin biosynthesis and signaling mechanisms (Figure 7A,B). Interestingly, a majority of the DEGs associated with the biosynthesis and signaling of indole acetic acid (IAA) were found to be dramatically increased during the development of male flowers. Notably, the presence of Aux/IAA and auxin response factor (ARF) genes was prominent, as shown in Figure 7C. Of the differentially expressed genes (DEGs) associated with signaling, 31 genes are classified as Aux/IAA, 17 genes are classified as ARF, and 13 genes are classified as GH3. The majority of the Auxin/IAA and ARF genes exhibited a greater expression pattern throughout male flower development, particularly at the fully developed flowering stage, in comparison to female flowers. Interestingly, among the genes implicated in the IAA signaling pathway, we discovered that the class of IAA genes maintained a high expression level during both female flower development and early male floral development at the M1 stage. In contrast, IAA expression in the full bloom stage was suppressed when compared to M0/F0 levels. Since IAAs are major transcriptional repressors in auxin signaling, it is impartial to assume that IAAs play a critical role in suppressing auxin signaling during the development of female flowers, while down-regulated IAA expression allows auxin signaling to occur during male flower development.

Moreover, a total of 12 differentially expressed genes (DEGs) related to the indole-3-acetic acid (IAA) metabolism pathway were discovered. Within these DEGs, seven YUC genes, which encode a key family of enzymes involved in IAA biosynthesis, exhibited significant upregulation during male floral development compared to female flower development. Among these DEGs, three YUC genes (*YUC2/LITCHI006663*, *YUC8/LITCHI025923*, and *YUC11/LITCHI011353*), which encoded one class of essential IAA biosynthesis enzymes, were dramatically elevated in male flower development but had low expression levels in female flowers (Figure 7D). In addition to YUC genes, two TAA1 and DAO enzyme genes were found to have significantly higher expression levels during male floral development compared to female flower development. Curiously, the VAS genes, which play a crucial role in the mechanism of IAA production, exhibited a contrasting pattern. These findings suggest that auxin plays a crucial role in the growth and differentiation of male flowers.

### 3.8. Validation of Auxin and Flower Development-Related Genes via qRT–PCR

**Figure 8:**
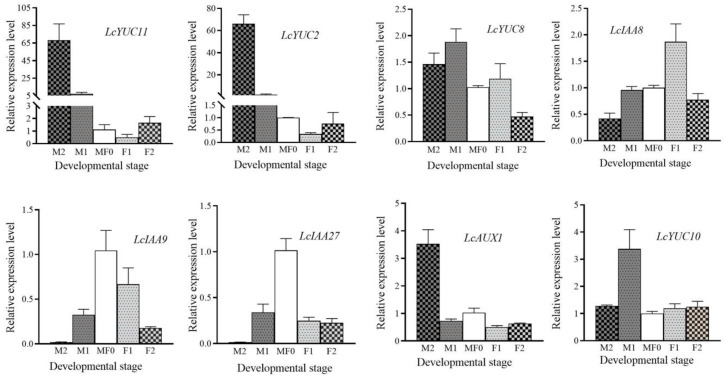
The relative expression of YUC and IAA class genes throughout male and female flower development. Key DEGs associated with floral sexual differentiation. Actin was used as an endogenous control. Data are presented as the mean ± SEM (*n* = 3). The relative expression levels of target genes were calculated via the 2^−ΔΔCt^ method.

To examine the expression profile of chosen genes involved in floral bud development and sexual differentiation, we use quantitative RT-PCR. The qRT-PCR data reveal that the expression of most genes is nearly persistent with the FPKM determined via the transcriptome dataset. The expression of YUC11/YUC2/YUC8/YUC10/AUX1 was higher in the male flower development stage (M1 and M2), but IAA29/IAA27 had an abundant expression level at the M0/F0 stage (Figure 8).

To better understand the elements that control the development of male and female flowers, we examined the transcription of genes associated with the biological pathways of “androecium development” and “gynoecium development” during different stages of flower development. We identified eight genes that displayed different expression patterns in male and female flowers, including *LcQRT2* (*QUARTET 2*)/*LITCHI004398*, *LcSWT12* (*SWEET 12*)/*LITCHI020289*, *LcROXY2* (*GLUTAREDOXIN 2*)/*LITCHI020788*, *LcATXR6* (*HISTONE-LYSINE N-METHYL TRANSFERASE 6*)/*LITCHI012318*, *LcWOX1* (*WUSCHEL-RELATED HOMEOBOX 1*)/*LITCHI023084*, *LcAGL11* (*AGAMEOUS-LIKE MADS-BOX PROTEIN 11)*/*LITCHI011705*, *LcEPFL1* (*EPIDERMAL PATTERNING FACTOR-LIKE PROTEIN 1*)/*LITCHI007305*, and *LcTT16* (*TRANSPARENT TESTA 16*)/*LITCHI006184*.

**Figure 9:**
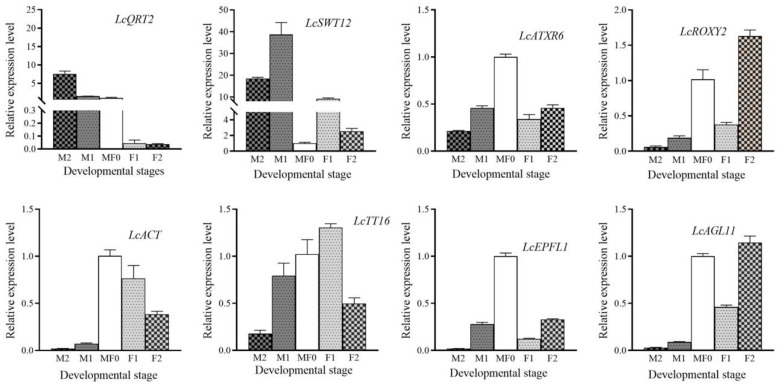
The relative expression of genes associated with androecium or gynoecium development. Actin was used as an endogenous control. Data are presented as the mean ± SEM (n = 3). The relative expression levels of target genes were calculated via the 2^−ΔΔCt^ method.

The expression of *LcQRT2* was significantly increased by more than fivefold in the M2 stage compared to that in the M/F0 stage (Figure 9). However, the expression during female flower development was negligible. The expression levels of *LcSWT12* were significantly higher during male floral development compared to female flower development. However, during male flower development, particularly in the M2 stage, the expression of these genes was dramatically suppressed. It is important to mention that *LcROXY2*, *LcATXR6*, *LcEPFL1*, and *LcTT16* did not exhibit significant differences between the M1 and F1 phases. However, the expression of *LcAGL11* and *LcWOX1* in the M1 stage differed from that in the F1 stage by more than five times.

## 4. Discussion

Research on the morphology of sex differentiation in plants reveals that the primary factor determining the sex difference is the growth of floral organs. Parthenos flowers, which lack fertilization, emerge from bisexual blooms by the degradation of either female or male organs [6]. The present research focused on examining the morphological and structural changes that occur throughout the development of litchi flower buds. The flowering pattern of litchi ‘Feizixiao’ cultivars was categorized into three distinct periods (Figure 1). The stages of development are as follows: F0/M0 corresponds to the stage of sepal formation and amphoteric primordium development, F1/M1 corresponds to the stage of the pistil and stamen growth, and F2/M2 corresponds to the time when female and male flowers are fully open. Auxin, a prominent phytohormone, regulates the various developmental processes that occur throughout the lifespan of plants. However, the role of auxin in floral sexual differentiation is limited.

### 4.1. Auxin Promotes Male Flower Development in Litchi

Auxin regulates plant development and growth in a dose-dependent way, with radial auxin concentration variations across plant tissues playing a pivotal role [31]. Plants have three naturally occurring chemicals that directly affect auxin activity: indole-3-acetic acid (IAA), 4-chloroindole-3-acetic acid (4-Cl-IAA), and phenylacetic acid (PAA). Among these molecules, IAA is the most extensively studied and understood, and synthesized via both tryptophan (Trp)-dependent and Trp-independent pathway [32,33]. Herein, we performed exogenous auxin (NAA) treatment, which induced male flower development in litchi, and the ratio of female flowers was substantially reduced, implying its putative key role in litchi male flower differentiation (Figure 1). In addition, we used LC-MS to identify auxin-related metabolites, yielding 26 compounds (Figure 2 and Figure 3). Fascinatingly, the majority of auxins were upregulated during litchi male flower bud development (M1) compared to the control (M0/F0). Our results are consistent with previous research showing that Auxin is involved in numerous stages of plant development and growth, including cell division, apical dominance, vascular differentiation, lateral/adventitious root production, and the development of fruits and flowers [34,35].

Moreover, tryptophan, TRA, IAA, MeIAA, and IAA-Ala levels were substantially higher in the male flowers compared to control (M0/F0) and female flowers, implying their putative role in floral sexual differentiation (Figure 2). Our findings correspond to previous research indicating that tryptophan, IAA, and other auxin-related chemicals play a crucial role in the growth and development of flowers, as noticed in rice. Specifically, a deficit of tryptophan resulted in reduced levels of IAA and aberrant development of floral organs [36]. In addition, the K-means clustering analysis categorized auxin metabolites into six distinct groups, each characterized by varying quantities of auxin-related metabolites at specific stages (Figure 3). The abundance of metabolites at specific stages of floral development suggests their involvement in those particular stages.

### 4.2. Auxin Finetunes the Intricate Mechanism of Male Flower Development

There has been a growing interest in understanding the physiological and molecular mechanisms of floral sexual differentiation in perennials through the application of RNA sequencing technology [37,38] in recent years, thereby giving new insight into potential pathways involved in the litchi floral sexual differentiation mechanism. Herein, the molecular mechanism underlying floral sex development and differentiation was investigated using different flower development stages of ‘Feizixiao’ as the study material through the application of RNA-seq technology. The data yielded clean bases of 5.68–7.04 GB, resulting in 33,657 unigenes after successfully removing redundancies using CD-hit and StringTie software. Additionally, the assembly process successfully identified 1761 novel genes (Figure S1, Supplementary Tables S1–S3). GO functional annotation showed that 12,944 genes were categorized into 38 functional branches, including cell components, biological processes, and molecular functions, while 6377 genes were enriched in 140 KEGG pathways (Figure 4, Figure 5 and Figure 6). However, the amount of unigenes and new genes varies depending on the sampling data and species [39].

The UpSet plot and Venn diagram unfold that the number of DEGs was higher between M/F0 vs. M1 stage followed by M0/F0 vs. F1 and F1 vs. M1 stage, respectively (Figure 4 and Figure 5). Furthermore, across all stages, the number of upregulated genes was greater than the number of downregulated genes, indicating that they played a specific role at each step. It has been proven that gene upregulation in response to certain treatments implies a vital function in the transcriptional regulation of specific developmental processes [29,40,41]. Similarly, a total of 12,466 DEGs were annotated on the Gene Ontology, while comparing the libraries M0/F0 vs. F1, M0/F0 vs. M1, and F1 vs. M1 yielded 1589, 2252, and 573 DEGs, respectively, in KEGG database (Figure 6 and Figure 7). Captivatingly, “Plant pathogen interaction” “Hormone signaling transduction”, and “MAPK signaling pathway” were highly enriched between M/F0 and F1 compared to M0/F0 and M1, our primary focus. Similar studies showed that “hormonal signaling transduction” and “MAPK signaling pathways” play a vital function in floral organ development [42,43,44,45].

### 4.3. Transcriptional Regulation of Floral Organ Development

We conducted additional research to determine if particular genes or signaling pathways could be linked to the various stages of flower bud development. The majority of DEGs associated with the signal transduction pathway of auxin and abscisic acid were significantly increased during male flower bud differentiation. Within the DEGs, the predominant hormones were primarily associated with auxin metabolism (Figure 7). A total of 74 DEGs associated with auxin production and signaling mechanisms were identified. The majority of these genes were elevated during the male flower development stages, providing strong evidence to support our idea that they play a crucial role in floral differentiation. Most of them fall under the category of auxin signaling, such as Aux/IAA, ARF, and GH3. In the male flowering stage, there was a noticeable increase in the expression of auxin-biosynthesis-related genes such as YUC, TAA1, DAO, and VAS enzyme genes compared to the control (M0/F0) and female stages (Figure 7 and Figure 8). Previous research has also demonstrated that YUC, TAA1, DAO, and VAS play an important function in the process of auxin production [46,47,48]. Likewise, YUCCA, TAA1, and WOX genes regulate the growth and formation of several floral and root organs in rice, Arabidopsis, and tomato [49,50]. In our study, three YUC genes (*YUC2*/*LITCHI006663*, *YUC8*/*LITCHI025923*, and *YUC11*/*LITCHI011353*), which encoded one class of essential IAA biosynthesis enzymes, were dramatically elevated in male flower development but had low expression levels in female flowers (Figure 7D). These findings suggest that auxin plays a crucial role in the growth and differentiation of male flowers.

Applying exogenous growth hormones has consistently proven to be a successful method for stimulating the development of female and male flowers in plants. The majority of research on plant sex differentiation primarily concentrates on the stage of flower bud differentiation. It is hypothesized that applying plant growth regulators at this time can alter the plant’s internal hormones, which serve as signals for inducing sex differentiation [51,52]. Additionally, we examined the expression pattern of genes involved in male and female flower development, such as QRT2/SWT/ROXY/ATXR, across the various stages of flower growth and differentiation (Figure 8 and Figure 9). QRT2 codes for a polygalacturonase enzyme that is necessary for the differentiation of pollen in the model crop Arabidopsis [53]. On the other hand, SWT12 codes for a protein belonging to the SWEET sucrose efflux transporter family, which is essential for the maturation of pollen [54]. The ROXY2 gene in Arabidopsis codes for a protein belonging to the CC-type glutaredoxin family. It has been observed that roxy1 roxy2 double mutants exhibit sterility and impaired pollen development [55]. ATXR6 is an H3K27 monomethyltransferase that plays a crucial role in the regulation of anther dehiscence, as demonstrated by Raynaud et al. [56]. WUS clade transcription factor WOX1 regulates ovule and pollen formation. Cucumber *CsWOX1* overexpression caused shorter filaments and degraded pollens [23], comparable to litchi female flowers (Figure 9). These findings indicate that *LcQRT2*, *LcSWT12*, *LcROXY2*, and *LcATXR6* are likely the crucial proteins responsible for the variation in anther development between male and female flowers. On the other hand, *LcWOX1*, *LcEPFL1*, *LcAGL11*, and *LcTT16* are likely involved in the formation of carpels and ovules. In short, our study reveals that certain hormones or their signals originate in nature throughout the flowering process to control the sexuality of flower buds. Additionally, disrupting the flowering process would impact the sexual differentiation of subsequent flowers within the same cluster.

## 5. Conclusions

In this study, we employed RNA-Seq, LC-MS/MS, and hormone application to investigate the molecular mechanism responsible for the sex determination of flowers in *Litchi chinensis* Sonn. The targeted metabolite findings indicated that the levels of auxin-related metabolites were predominantly elevated in male flowers as compared to female flowers. The transcriptome study revealed that the male flowers had a greater number of DEGs compared to both the control stage and the female flowering stages. Additionally, the number of upregulated DEGs was higher than the number of downregulated DEGs. Furthermore, we found that auxin biosynthesis and signaling-related metabolites play an important role in floral development, as revealed by KEGG, with the majority of them being elevated. These results provide valuable information on how to comprehend the molecular mechanism of flower sex determination and serve as a basis for identifying candidate genes.

## Figures and Tables

**Table 1:** Transcription factors detected in the significant DEGs.

	M0/F0 vs. F1		M0/F0 vs. M1		F1 vs. M1		Sum
Transcription Factors	Up	Down	Up	Down	Up	Down	
AP2	35	4	36	10	5	3	93
ARF	4	1	5	0	1	1	12
AUX/IAA	5	3	5	3	0	0	16
bHLH	9	10	11	11	5	1	47
bZIP	5	7	9	8	2	1	32
Dof	5	4	3	6	0	0	18
GATA	4	0	5	2	0	1	12
YABBY	1	2	0	3	0	1	7
C2H2	13	5	12	11	0	4	45
GRAS	7	2	10	4	1	0	24
HSF	8	0	9	1	1	0	19
MADS	3	3	2	8	3	3	22
MYB	10	10	14	14	4	1	53
MYB-related	5	11	11	11	3	0	41
NAC	18	7	0	8	4	0	37
SNF2	6	0	24	0	0	0	30
Tify	4	1	4	0	0	0	9
WRKY	8	11	14	13	3	0	49
Total TFs	150	81	174	113	32	16	566

## Data Availability

The raw data for the digital gene expression analysis were also deposited in the NCBI with Submission ID: SUB14661989 and BioProject ID: PRJNA1147420.

## Supplementary Materials

The following supporting information can be downloaded at: https://www.mdpi.com/article/10.3390/plants13182592/s1, Figure S1: Total raw read counts found from the litchi transcriptome dataset; Figure S2: PCA and correlation analysis of sampling data during floral bud development; Table S1: Endogenous auxin-related metabolites detected during litchi flower differentiation after NAA application; Table S2: Statistical analysis of litchi flower RNA-seq data; Table S3: Statistics for the sample and reference genome comparisons; Table S4: List of primers used in the assay.
