# Supplementary material for: Targeted Metabolites and Transcriptome Analysis Uncover the Putative Role of Auxin in Floral Sex Determination in Litchi chinensis Sonn
Source: Plants (Basel). 2024 Sep 16;13(18):2592. doi: 10.3390/plants13182592 (PMC11435090; doi:10.3390/plants13182592)
Supplement: Supplementary file 1 [file plants-13-02592-s001.zip › Report English Version.pdf]

DeepL

Subscribe to DeepL Pro to translate larger documents.
Visit www.DeepL.com/pro for more information.

Personalbio [illegible]

派森诺 | Personalbio

项目结题报告书

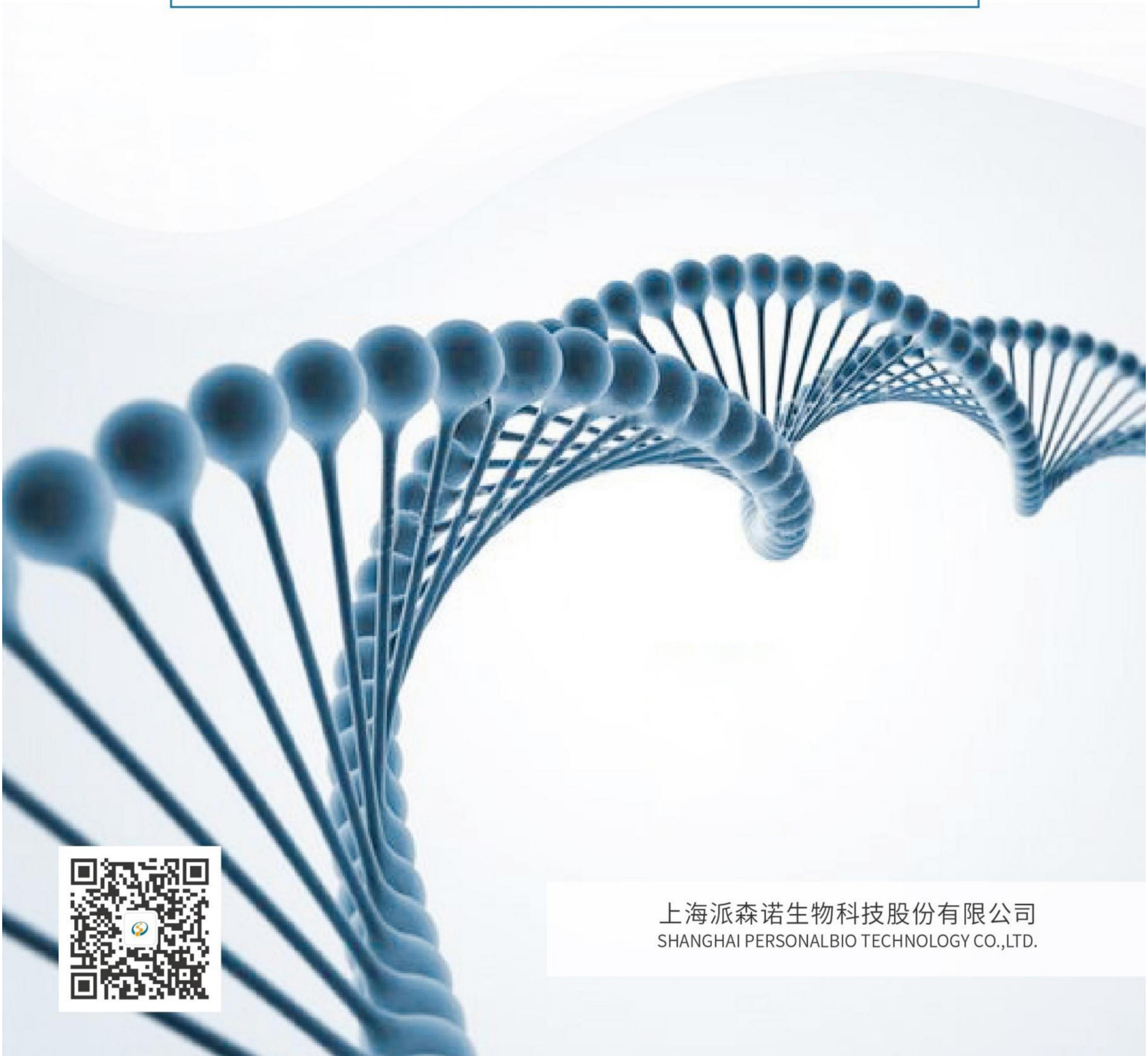

上海派森诺生物科技股份有限公司
SHANGHAI PERSONALBIO TECHNOLOGY CO.,LTD.

catalogs

1. Overview of the overall project process

Project Information ---------- 5
Analyzing Processes ---------- 5

2. Project analysis results

2.1 Raw data processing and quality control

Data collation ---------- 7
Data Filtering ---------- 7
Base Mass Distribution ---------- 8
Distribution of base content ---------- 9
R eads mean mass distribution ---------- 1 0

2.2 Analysis of species composition

Comparison results statistics ---------- 1 1

2.3 Quality control of comparison results

Gene coverage is one degree ---------- 1 2
Saturation analysis ---------- 1 3
Comparison of regional distribution statistics ---------- 1 4

2.4 Expression analysis

Expression analysis ---------- 1 5
Expression interval statistics ---------- 1 7
Distribution of known genotypes in the sample ---------- 1 8
F PKM density distribution ---------- 1 8
Correlation analysis ---------- 2 0
P CA Analysis ---------- 2 0

2.5 Expression difference analysis

Differential Expression Analysis ---------- 2 1
Differential Expression Results Statistics ---------- 2 2
Volcano map ---------- 2 3
Cluster analysis ---------- 2 4
Trend analysis ---------- 2 5
Differential Gene Wayne Diagrams ---------- 2 6
Genome Circle Map ---------- 2 9
Protein Network Interaction Analysis ---------- 2 9
Analysis of exon differences ---------- 3 0

2.6 enrichment analysis

G O enrichment analysis ---------- 3 1
K EGG enrichment analysis ---------- 3 4

2.7 Functional potential prediction

Transcript splicing ---------- 3 7

Analysis of new transcripts ---- 37
UTR Optimization Analysis ---- 38
Differential Variable Shear Analysis ---- 38
2.8 variation detection
SNP ---- 40
InDel ---- 42
Annovar Notes ---- 43
Mutation type statistics ---- 45
2.9 transcription factor analysis
Transcription factor family distribution ---- 46
Distribution of differentially expressed transcription factors ---- 47
2.10 appendice
Database Introduction ---- 49
Introduction to the software used ---- 50
Noun Explanation ---- 51
Common Terminology ---- 51
Frequently Asked Questions ---- 59
References ---- 60

1. Overview of the overall project process

Project information

Item number	TR202212261020DRGP
Contract number	YF20191017
Proposal number	TPL2022121188
The name of the project	Resequencing of 300 samples of maize
Project category	Eukaryotic parametric transcriptome analysis
Project type	Standard analysis
Completion date	2022/12/29 14:18:14
After-sales mailbox	transsupport@personalbio.cn

Analyze the process

The raw downstream data (Raw Data) is first filtered, and the high quality sequences (Clean Data) obtained after filtering are compared to the reference genome of the species. Based on the comparison results, the expression amount of each gene is calculated. Based on this, the samples were further analyzed for expression difference analysis, enrichment analysis and clustering analysis. The Reads on the comparison were spliced and the transcript sequences were reduced.

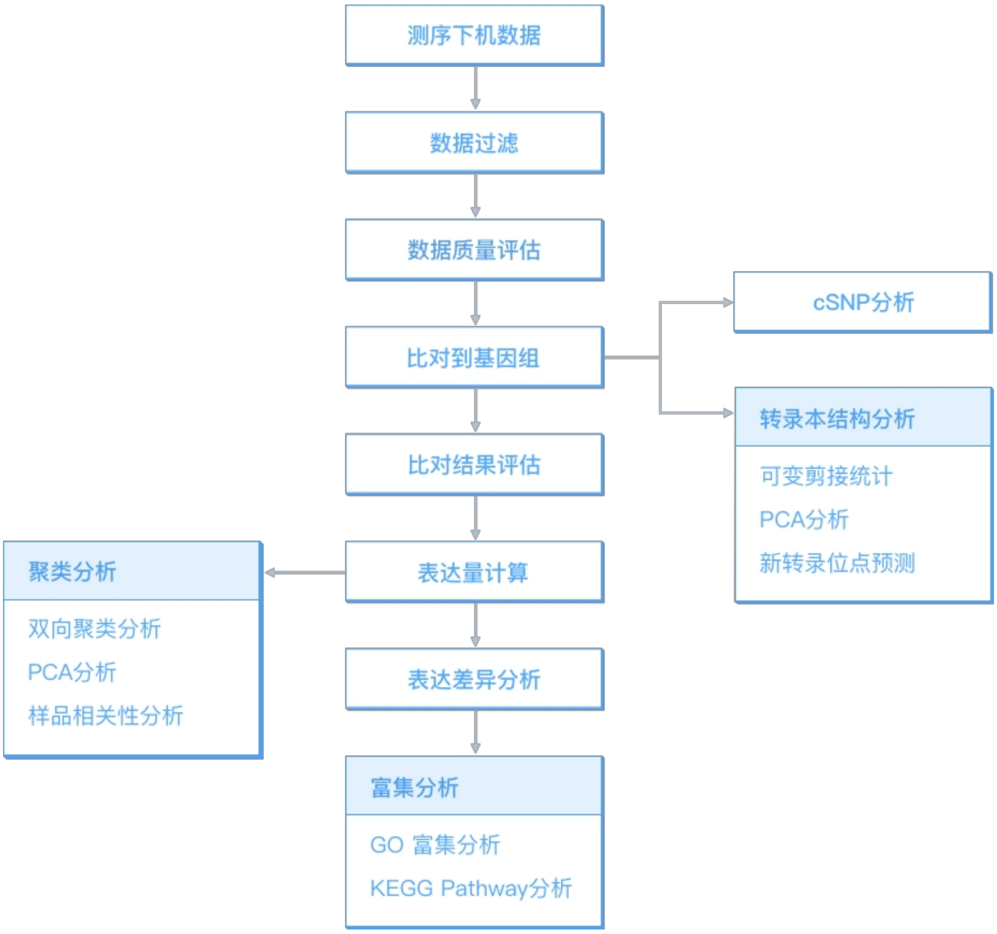
测序下机数据
数据过滤
数据质量评估
比对到基因组
cSNP分析
转录本结构分析
可变剪接统计
PCA分析
新转录位点预测
比对结果评估
表达量计算
聚类分析
双向聚类分析
PCA分析
样品相关性分析
表达差异分析
富集分析
GO 富集分析
KEGG Pathway分析

2.Project analysis results

2.1 Raw data processing and quality control

Data collation

The samples were up-sequenced to obtain the image files, which were transformed by the software that came with the sequencing platform to generate the Raw Data of FASTQ, i.e., the down-sequenced data. The Raw Data of each sample were counted separately, including sample name, Q30, percentage of ambiguous bases, and Q20(%) and Q30(%).

Analysis results.

Data collation table

Sample	Raw_Read_Number	Raw_Bases	Raw_Q30_number	Raw_N_rat
A1	38144078	5759755778	5499717597	0.001619
A2	38341930	5789631430	5512303864	0.001607
A3	42668624	6442962224	6157020529	0.001608
B1	39239266	5925129166	5673125664	0.001634
B2	44138454	6664906554	6279482057	0.002696
B3	39420128	5952439328	5696634871	0.00162
C1	42168782	6367486082	6077676800	0.001628
C2	42209184	6373586784	6082397238	0.001626
C3	35909030	5422263530	5177368204	0.001627
D1	37682080	5689994080	5431080114	0.001618

Sample: Sample name Reads No.: Total number of Reads Bases (bp): Total number of bases Q30 (bp): Total number of bases with 99.9% or more base recognition accuracy N (%): Percentage of ambiguous bases Q20 (%): Percentage of bases with 99% or more base recognition accuracy Q30 (%): Percentage of bases with 99.9% or more base recognition accuracy Percentage of bases with base recognition accuracy of 99.9% or more

Data Filtering

Sequencing data contains some joints and low-quality reads, which will cause great interference to the subsequent analysis of information, so it is necessary to further filter

the sequencing data. The criteria for data filtering mainly include:

1) Cutadapt was used to remove the 3' end of the junction, and the removed portion had at least 10 bp Overlap with known junctions.

(AGATCGGAAG), allowing for 20% base mismatches;

2) Removal of Reads with average mass

fractions below Q20; Analytical results.

Data Filter Table

Sample	Trimmed_Read_Number	Trimmed_Bases	Useful_read%	Useful_
A1	35320212	5333352012	92.59	92
A2	35483680	5358035680	92.54	92
A3	39444484	5956117084	92.44	92
B1	36326546	5485308446	92.57	92
B2	40818766	6163633666	92.47	92
B3	36485760	5509349760	92.55	92
C1	39048936	5896389336	92.6	9
C2	39082320	5901430320	92.59	92
C3	33253856	5021332256	92.6	9
D1	34895464	5269215064	92.6	9

Sample: Sample name Clean Reads No: number of high-quality sequence reads Clean Data (bp): number of high-quality sequence bases Clean Reads %: high-quality sequence reads as a percentage of sequencing reads Clean Data %: high-quality sequence bases as a percentage of sequencing bases

base mass distribution

The sequencing error rate is affected by a combination of several factors, including the sequencer itself, sequencing reagents, and samples. For RNASeq technology, the sequencing error rate distribution is characterized by two features:

1) Sequencing error rates increase with the length of sequenced sequences due to the consumption of chemical reagents during the sequencing process, a characteristic of all Illumina high-throughput sequencing platforms;

2) The position of the first 6 bases (i.e., the length of the random primer required for reverse transcription during library construction) also occurs with a higher rate of

sequencing errors, which are caused by incomplete binding of the random primer to the RNA template.

We evaluated the base quality at individual positions using a single base quality distribution plot of the sequencing data. In general, Reads have lower base quality at the 5' and 3' ends and higher base quality in the middle part. Most of the sequences have a base quality of 20 or more, which represents a good sequencing quality.

Analysis results.

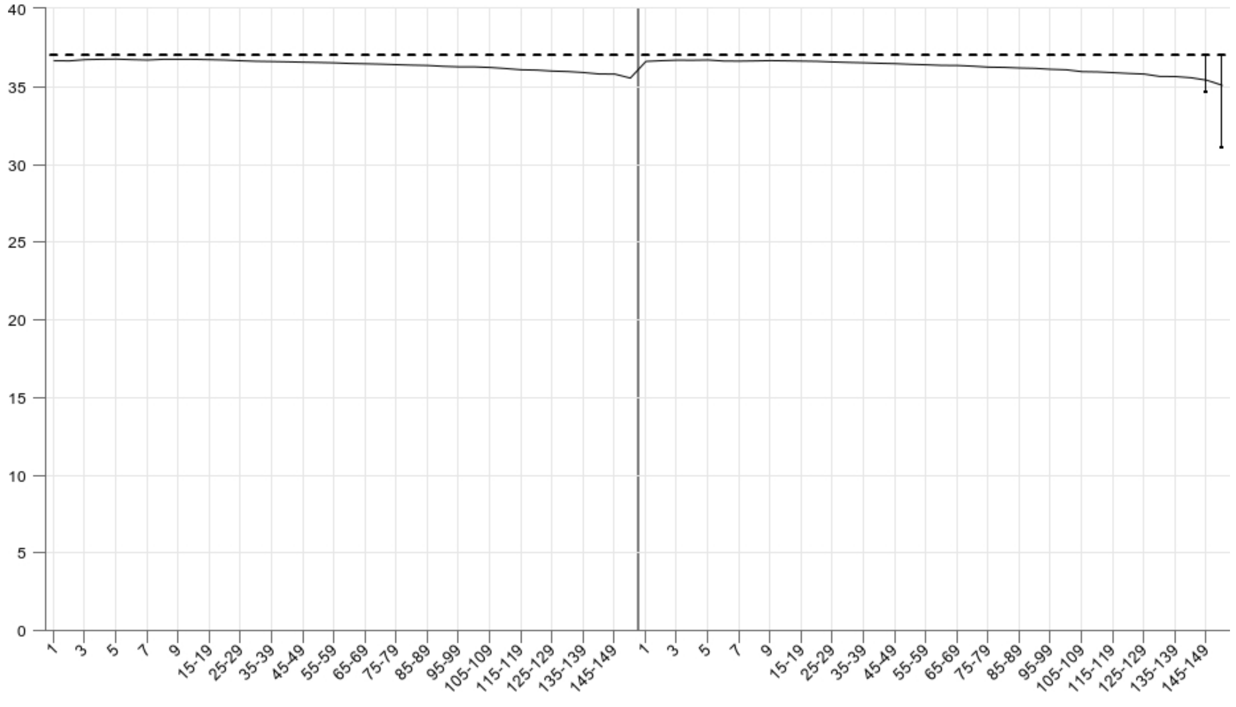

The horizontal coordinate is the base position (5'->3') in the Reads, and the vertical coordinate is the base Q value of the corresponding site.

Distribution of base content

The base content distribution is generally used to detect the presence or absence of AT and GC segregation. For RNASeq, given the randomness of sequence interruption and the principle of equal G/ C and A/T content respectively, theoretically the GC content in each sequencing cycle is equal, the AT content is equal (AT segregation and/or GC segregation may occur in the case of strand-specific library building), and it is basically stable and constant in a horizontal line throughout the sequencing process. However, in the existing high-throughput sequencing technology, the 6bp random primers used in reverse transcription synthesis of cDNAs cause a certain preference in the nucleotide composition of the first few positions, and such fluctuations are normal.

Analysis results.

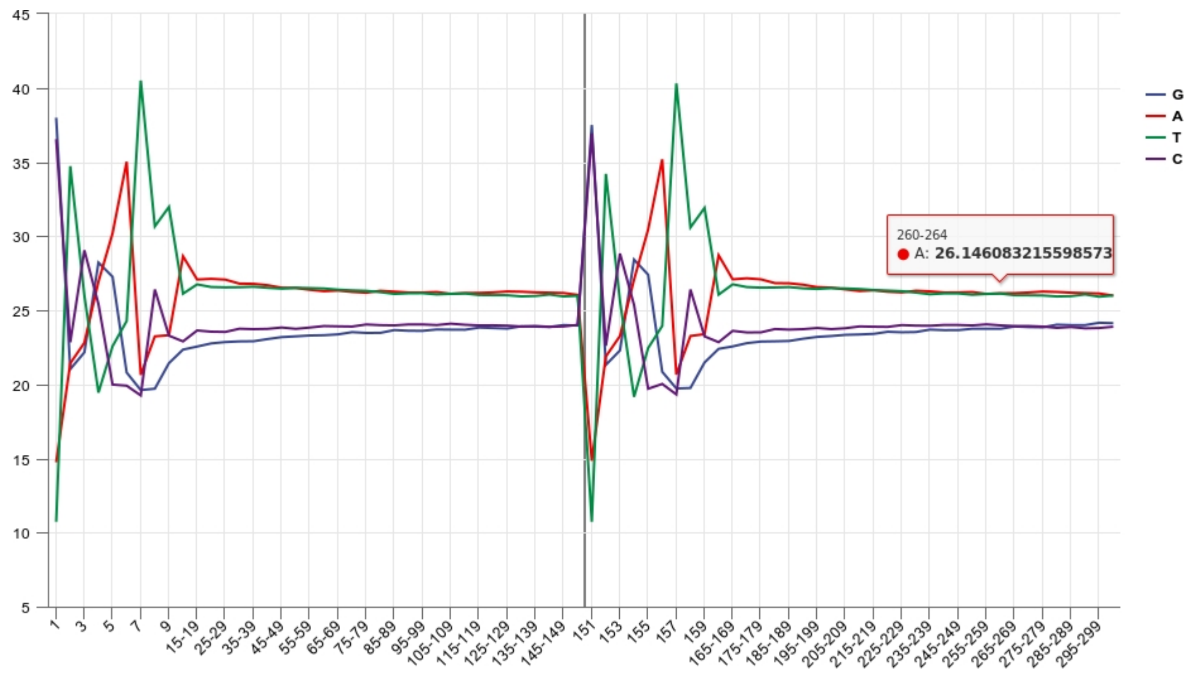

The horizontal coordinate is the base position (5'->3') in the Reads, and the vertical coordinate is a count of the percentage of a base at that site.

Reads Mean Mass Distribution

Reads average quality distribution is mainly used to detect the average quality distribution of sequencing data. The peak tip represents the sequencing quality of the main Reads, and the peak width represents the overall sequencing quality distribution; a larger peak width or front trailing peak indicates that the quality of some parts of the sequencing data is low, and a lower value of the peak tip indicates that the overall sequencing results are poor, while a lack of peak tip indicates that the overall quality of the Reads is very good.

Analysis results.

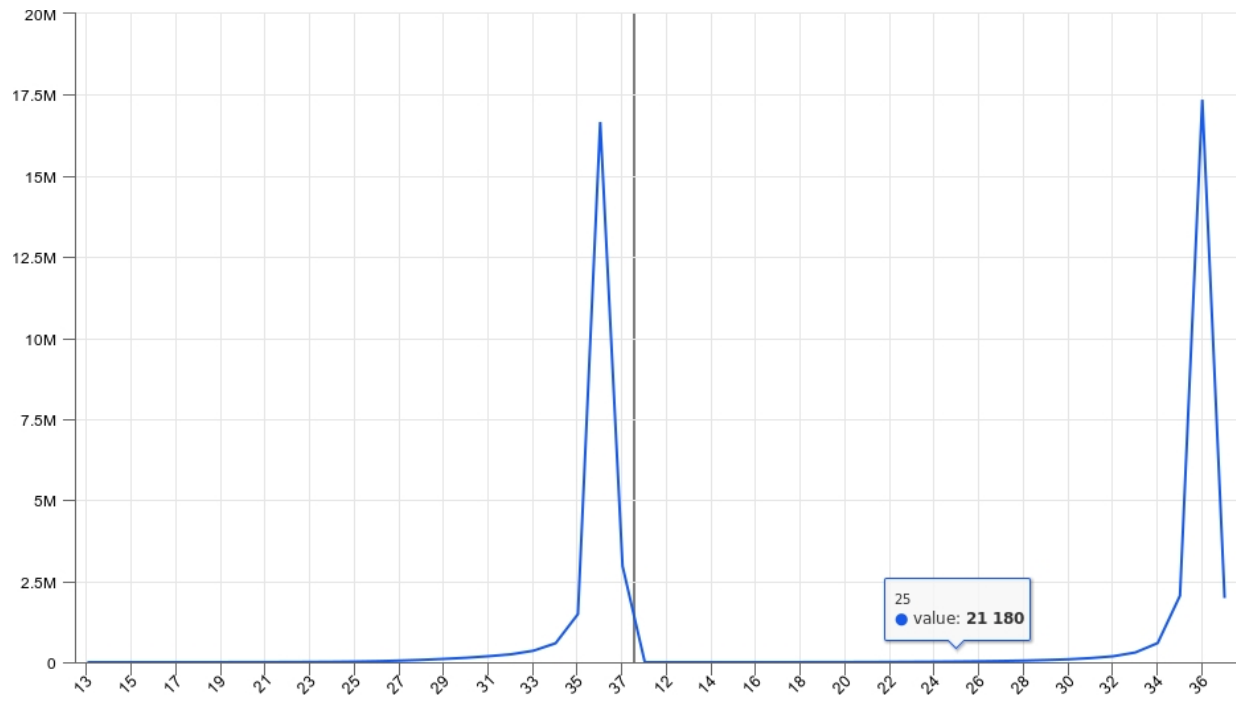

The Reads quality distribution is plotted with the horizontal coordinate indicating the average quality of the Reads and the vertical coordinate being the number of Reads corresponding to the average quality value.

2.2 comparison analysis

Comparison results statistics

Filtered Reads were aligned to the reference genome using TopHat2's upgraded HISAT2 (http://ccb.jhu.edu/software/hisat2/index.shtml) software. HISAT2 is faster and less resource-intensive using the improved BWT algorithm. HISAT2 uses default parameters for non-strand-specific libraries, while strand-specific libraries require the library type to be specified (i.e., use --rna-strandness RF for first, --rna-strandness FR for second). If the reference genome is selected appropriately and there is no contamination in the associated experiments, the Mapping percentage of sequenced sequences will generally be higher than 70%. the reason when the Mapping percentage is low may be:

1) The reference genome is poorly assembled, or the species measured is distantly related to the reference genome;

2) The Mapping Rate is relatively low due to the special pre-processing of the sample or the high variability of the sample relative to the reference genome.

Analysis results.

Comparative results statistics table

Sample	Clean_Reads	Total_Mapped	Multiple_Mapped	Uniquely_

A1	35320212	34034409 (96.36%)	1807296 (5.31%)	32227113
A2	35483680	34158619 (96.27%)	1821961 (5.33%)	32336658
Sample	Clean_Reads	Total_Mapped	Multiple_Mapped	Uniquely_
A3	39444484	37955196 (96.22%)	2105807 (5.55%)	35849389
B1	36326546	34950678 (96.21%)	1867269 (5.34%)	33083409
B2	40818766	39045338 (95.66%)	2098476 (5.37%)	36946862
B3	36485760	34979441 (95.87%)	1881711 (5.38%)	33097730
C1	39048936	37597550 (96.28%)	1886790 (5.02%)	35710760
C2	39082320	37697633 (96.46%)	1821696 (4.83%)	35875937
C3	33253856	32101348 (96.53%)	1755636 (5.47%)	30345712
D1	34895464	33588231 (96.25%)	1741204 (5.18%)	31847027

Sample: Sample Clean Reads: Total number of sequences used for comparison Total Mapped: total number of sequences aligned to the reference genome, in percent Total Mapped / Clean Reads Multiple Mapped: total number of sequences aligned to multiple positions, in percent Multiple Mapped / Total Mapped Uniquely Mapped: total number of sequences aligned to only one position, in percent Uniquely Mapped / Total Mapped Uniquely Mapped: total number of sequences aligned to only one position, in percent Uniquely Mapped / Total Mapped

2.3 Quality control of comparison results

Gene coverage at one degree

Demonstrates sequence coverage on the 5' to 3' regions of all genes for each sample, which is used to assess the homogeneity (or whether there is a bias) of the sequencing results. Ideally, the distribution of Reads over all expressed genes should show a homogenized distribution.

> Analysis results.

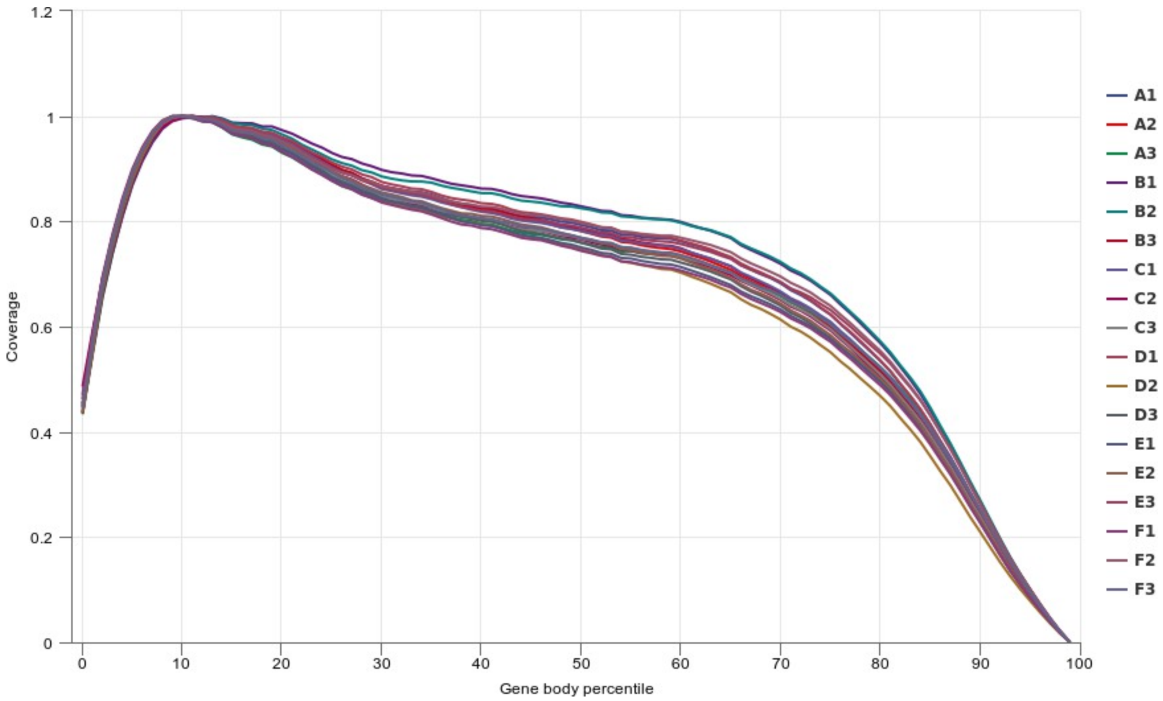

The horizontal coordinate is the percentage of the base length of a single gene to the total base length, 0 indicates the 5' end of the gene,100 indicates the 3' end of the gene; the vertical coordinate is the sum of the number of sequence entries in the corresponding intervals on the horizontal axis position of all genes compared to the total number of genes. The figure reflects the results of the overlay of the coverage of all genes, and the vertical coordinate of each point in the curve indicates the number of all sequences of all genes at that relative scale position; the curve reflects whether the sequences obtained from sequencing are evenly distributed over the genes. If there are no obvious bias fronts, the sequencing is unbiased.

Saturation analysis

We used RSeQC to analyze expression saturation, i.e., we sampled the sequencing results at 5%, 10%, 15%.... ...100%, calculate all gene expressions separately for different sampling ratios (i.e., 20 times for each gene), and then compare with the actual expression (assuming that the 100% sampling case results in the actual expression) to obtain the relative error.

The main purpose of saturation analysis is to assess whether the measured data volume is sufficient for the correct calculation of gene expression. Theoretically, the expression of a gene deviates greatly from the actual expression in the case of a small amount of data, and when the amount of data reaches the saturation threshold, the amount of data grows further and the expression of the gene is nearly unchanged, at which point the expression of the gene is no longer affected by the amount of data.

Analysis results.

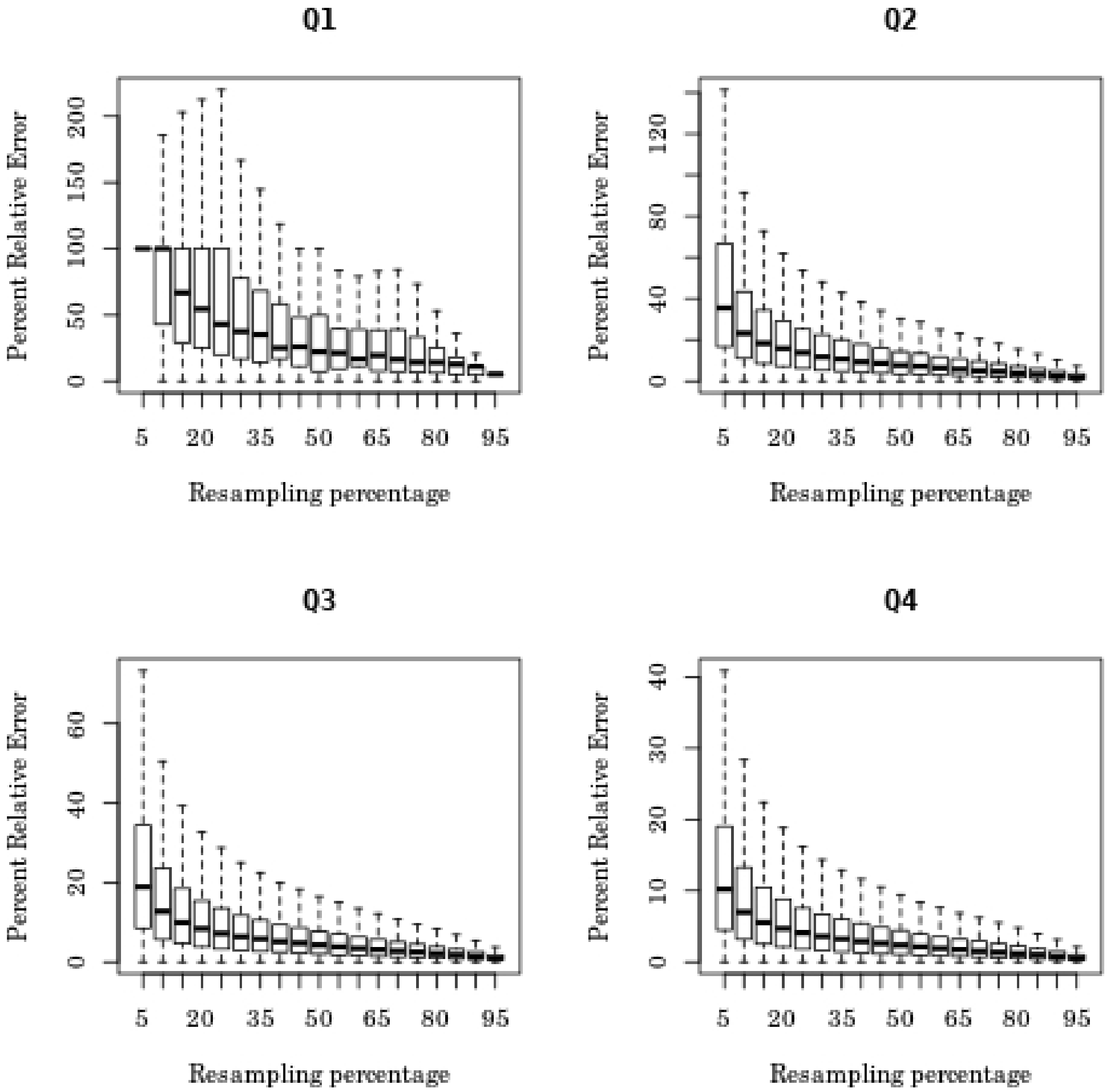

The horizontal coordinate is the ratio of resampling, and the vertical coordinate is the relative error between the expression and the actual expression of the gene at that ratio. Genes were categorized into four groups according to their expression levels; Q1: genes with expression levels in the bottom 25%; Q2: genes with expression levels in the bottom 25%-50%; Q3: genes with expression levels in the top 25%-50%; Q4: genes with expression levels in the top 25%. percent of genes; Q2: genes whose expression levels reside in the inverse 25-50 percent; Q3: genes whose expression levels reside in the top 25-50 percent; Q4: genes whose expression levels reside in the top 25 percent.

Comparison of regional distribution statistics

The distribution of Reads compared to the genome was counted, and the localized regions were classified into CDS (coding regions), and Intron (introns),
Intergenic (intergenic region) and UTR (5' and 3' untranslated region).

In species with more fully annotated genomes, reads aligned to the CDS (coding region) are usually the most abundant Reads aligned to the Intron (intronic) region originate from pre-mRNA residues or result from intron retention events that occur during variable shearing,

while Reads aligned to the Intergenic (intergenic region) may be transcribed from novel genes or new non-coding RNAs

Analysis results.

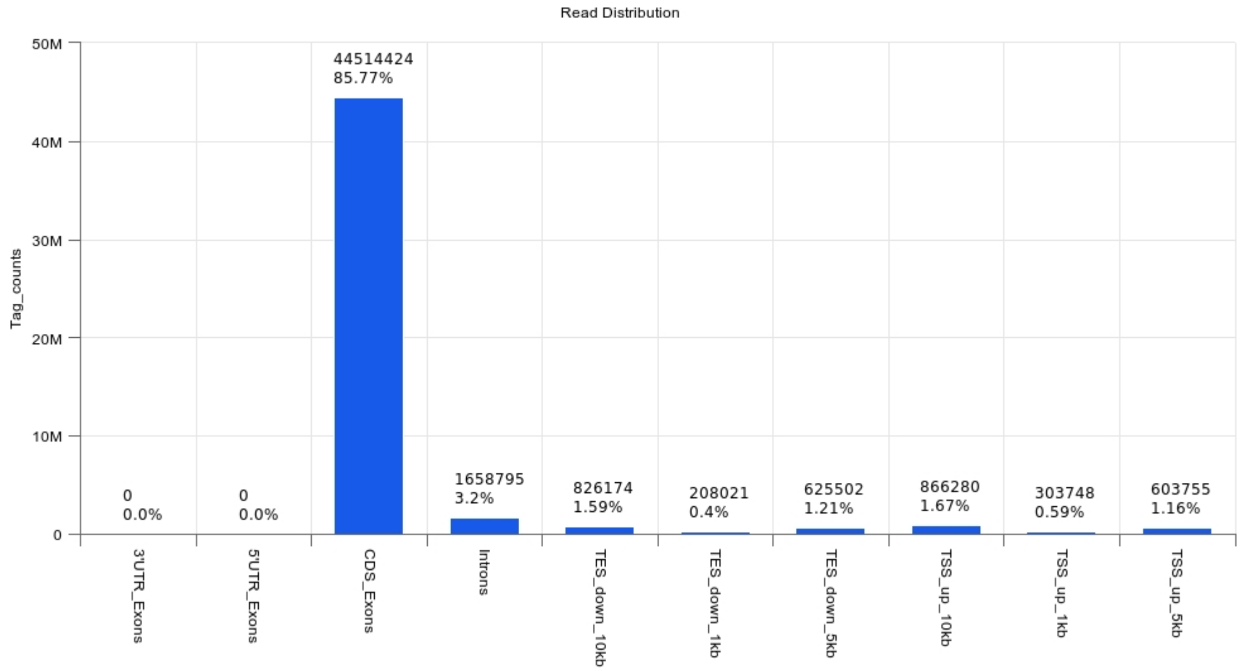

The horizontal coordinate is the different annotated regions of the gene and the vertical coordinate is the number of reads compared to the different regions.

2.4 Expression analysis

Expression analysis

We used HTSeq to statistically compare the Read Count value of each gene as the raw expression of the gene, and the Reads count is positively correlated with the true expression level of the gene, as well as the length of the gene and the sequencing depth. In order to make gene expression levels comparable across genes and samples, we used FPKM to normalize the expression (Nosrmalization), FPKM (Fragments Per Kilo bases per Million fragments) is the number of fragments from a gene per kilobase length per million fragments. FPKM (Fragments Per Kilo bases per Million fragments) is the number of fragments per million fragments per kilobase length from a gene, and for Pair-End sequencing, there are two Reads for each Fragment, and FPKM only counts the number of Fragments that can be matched to the same transcript by both Reads. In the reference transcriptome, we generally consider that genes with FPKM>1 are expressed. This threshold is recommended by mainstream journals, and can also reflect the gene expression level well.

$$\text{FPKM} = \frac{total\ exon\ reads}{mapped\ reads\ (millions) * exon\ length\ (KB)}$$

The statistical method is as follows: first read the gene structure annotation information (GTF file), and then compare the results with the gene structure and count the results.HTSeq has three statistical schemes, as shown in the figure below, the difference is that when a Read covers only part of a gene region or part of a gene's intron region, the

Union scheme and the Intersection_ nonempty scheme determine that the Read belongs to the gene, while the Intersection_strict scheme determines that the Read does not belong to any gene. nonempty scheme determines that the Read belongs to the gene, while the Intersection_strict scheme determines that the Read does not belong to any gene; when a Read covers all of a gene and part of another gene, the Union scheme determines that the Read belongs to both genes, and the Intersection_strict scheme and the Intersection_nonempty scheme determine that the Read belongs to both genes. strict and Intersection_nonempty schemes identify the Read as belonging to the first gene. If there is no special requirement, the Union scheme should be used, which is more robust (in case of strand-specific libraries, it is also necessary to determine whether the orientation of the Feature is consistent with that of the annotation).

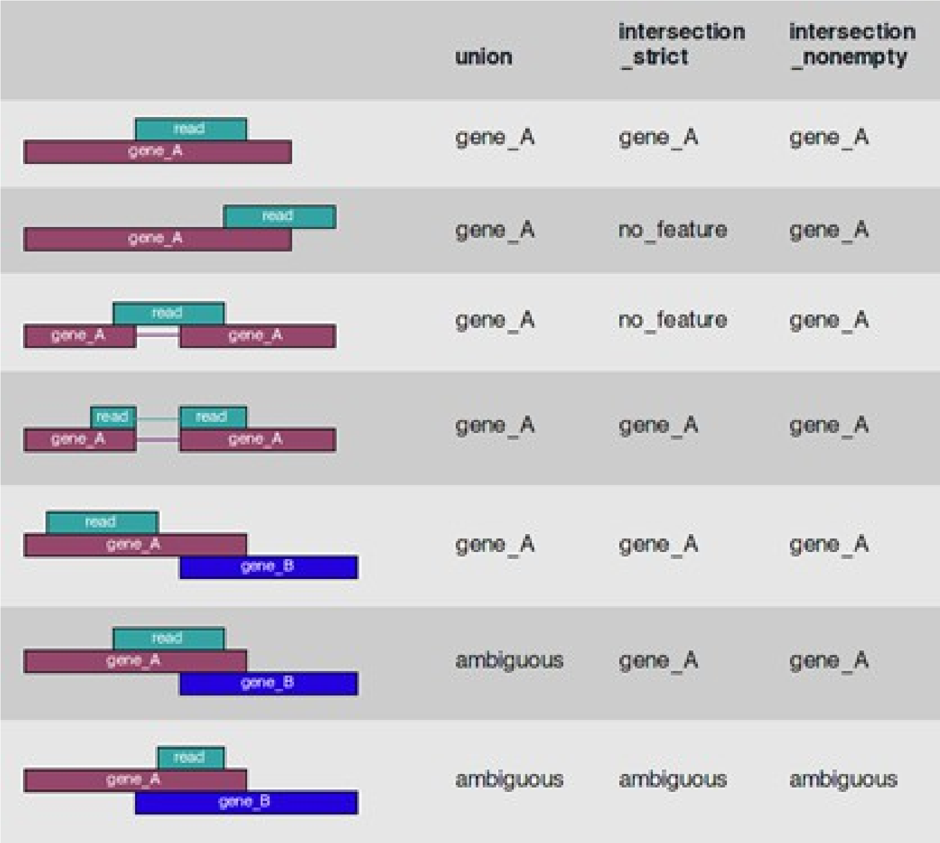

Analysis results.

Expression Analysis Scale (EAS)

Gene_ID	A1:read count	A1:fpkm	A2:read count
BnaA01G0000100ZS	0	0	0
BnaA01G0000200ZS	1	0.0864718547008064	0
BnaA01G0000300ZS	0	0	0
BnaA01G0000400ZS	2	0.399659832650786	1
BnaA01G0000500ZS	24	1.03577902182454	26
BnaA01G0000600ZS	0	0	1
BnaA01G0000700ZS	0	0	0
BnaA01G0000800ZS	0	0	0

Gene_ID	A1:read count	A1:fpkm	A2:read count
BnaA01G0000900ZS	0	0	0
BnaA01G0001000ZS	2	0.0568667039682465	5

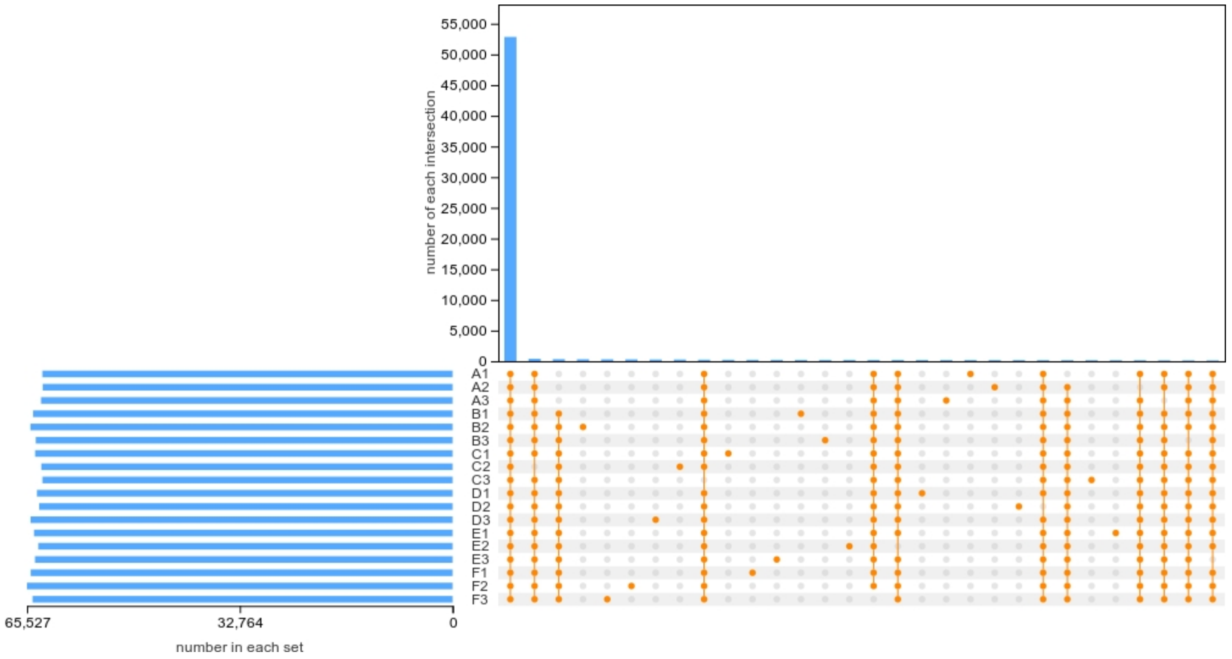

number in each set indicates the number of all genes identified in each sample; number of each intersection indicates the number of genes identified in multiple samples; the line of all points in the abscissa indicates the number of genes common to all samples, and the rest of the single points, or the line of multiple points, indicate the number of genes specific to the sample in question. The remaining single or multiple point lines indicate the number of unique genes identified in the relevant samples.

Expression interval statistics

According to the table of expression calculation results, the expression amount was divided into different intervals, and the number of genes within different expression amount intervals was counted for each sample.

Analysis results.

Expression interval statistics

Type	A1	A2	A3	B1	B2	B3	C1
0~0.01	37766	37814	37557	36335	35927	36755	3664
0.01~0.1	2329	2299	2488	2252	2613	2224	251

0.1~1	12361	12265	12264	12491	12819	12709	1274
Type	A1	A2	A3	B1	B2	B3	C1
1~10	28852	28783	28723	29705	29445	29650	3028
10~100	18400	18547	18703	19008	18954	18354	1739
100~1000	1194	1197	1169	1114	1146	1208	133
>1000	17	14	15	14	15	19	12

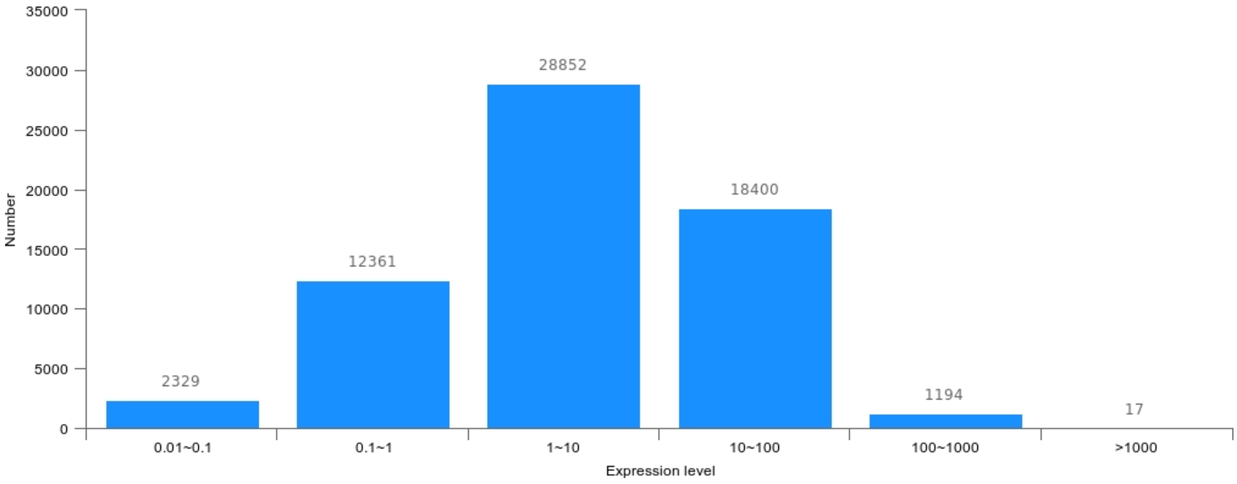

The horizontal coordinates indicate the range of expression values and the vertical coordinates indicate the number of genes in that expression interval.

Distribution of known genotypes in the sample

Expression statistics of different gene types in the annotation file of the

> genome based on the results of expression. Analysis results.

Table of distribution of known genotypes in the sample

Sample	A1	A2	A3	B1	B2
protein_coding	14017523	14073926	15641706	14407996	160169

The first row is the name of the sample and the first column is the different gene types, the types being derived from the annotation file of the genome.

FPKM density distribution

It is generally recognized that the expression analysis of the transcriptome includes three horizontal levels, i.e., gene expression level, transcript expression level, exon expression water
Ping. Expression of different shear forms of the same gene may cause very different biological effects. the FPKM density distribution enables a holistic view of the

The expression pattern of all genes in the sample is generally characterized by a large majority of moderately expressed genes and a small percentage of low and highly expressed genes.

Analysis results.

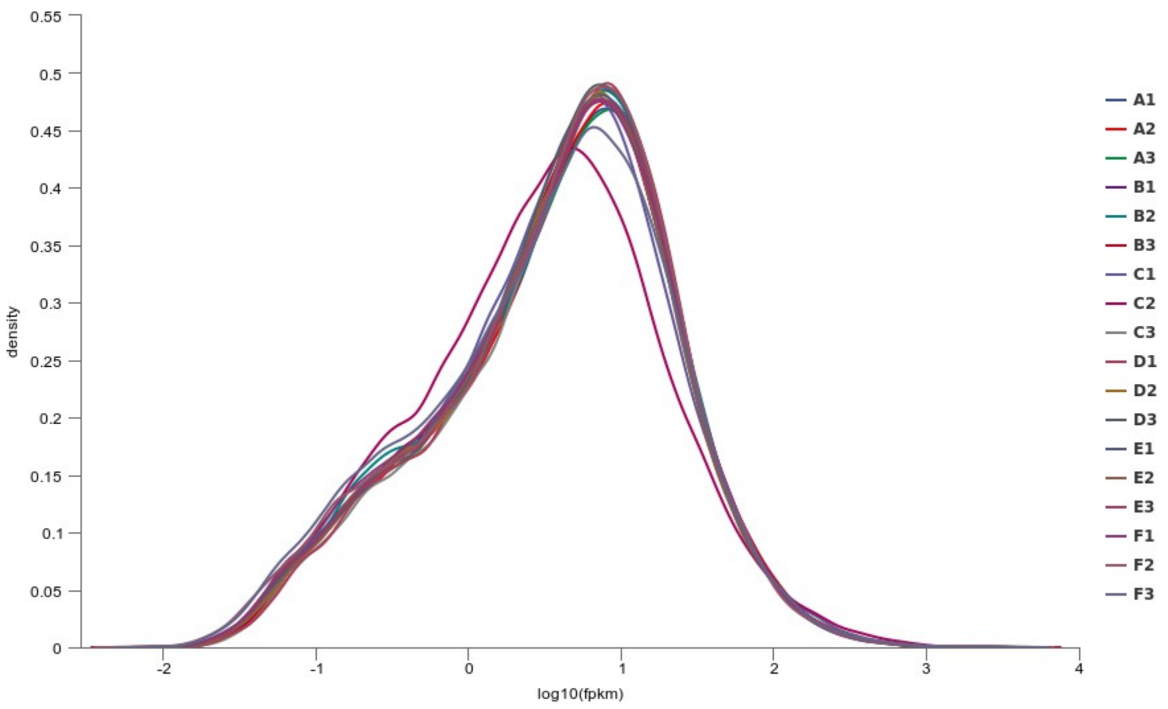

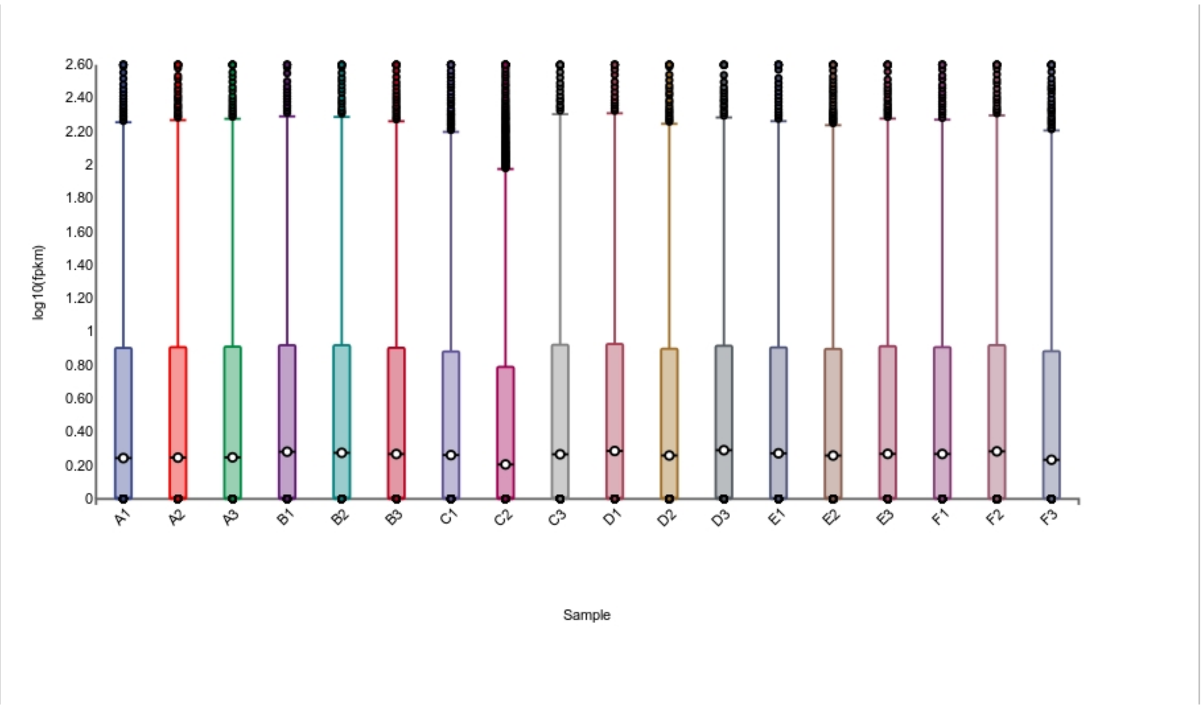

Density plot: horizontal coordinate is the log10(FPKM) value of the gene, vertical coordinate is the density of the gene distribution corresponding to the amount of expression; violin plot: the horizontal line in the middle of the box shape is the median, the upper and lower edges of the box shape are 75%, and the upper and lower limits are 90%. The outer shape is the kernel density estimate.

correlation analysis

The correlation of gene expression levels between samples is an important indicator to test the reliability of the experiment and the reasonableness of sample selection, and the correlation of gene expression levels between samples should be checked before doing differential expression analysis.

We used the Pearson correlation coefficient to indicate the correlation of gene expression levels between samples, and the closer the correlation coefficient is to 1, the higher the similarity of expression patterns between samples. In general, correlation coefficients between 0.8 and 1 are extremely strong correlations, and if the correlation coefficient between biological replicates is lower than 0.8, it indicates a low correlation between samples.

Analysis results.

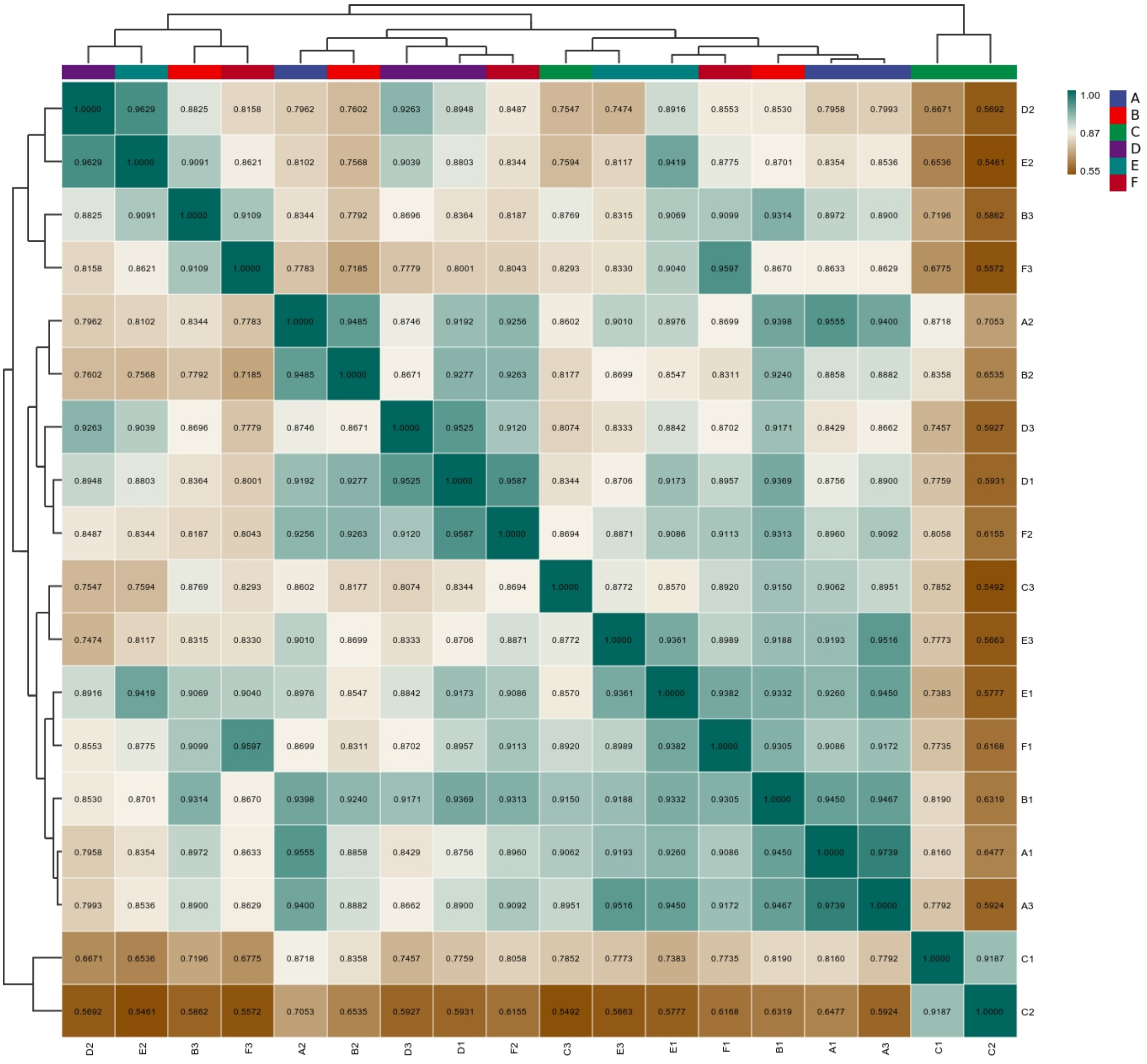

The left side and the top side show the sample clustering situation, the right side and the bottom side of the figure show the sample names, and the different colored squares represent the high and low correlation situation of the two samples.

PCA analysis

PCA PrincipalComponents Analysis (PCA), reduces high-dimensional data to two or three dimensions by linear transformation, while maintaining the features that contribute the most to the variance of each party, i.e., reducing the complexity of the data. When there are multiple samples, we use the R language's

DESeq software package, PCA principal component analysis was performed on each sample based on expression.PCA analysis clusters similar samples together, with closer distances indicating higher similarity between samples.

Analysis results.

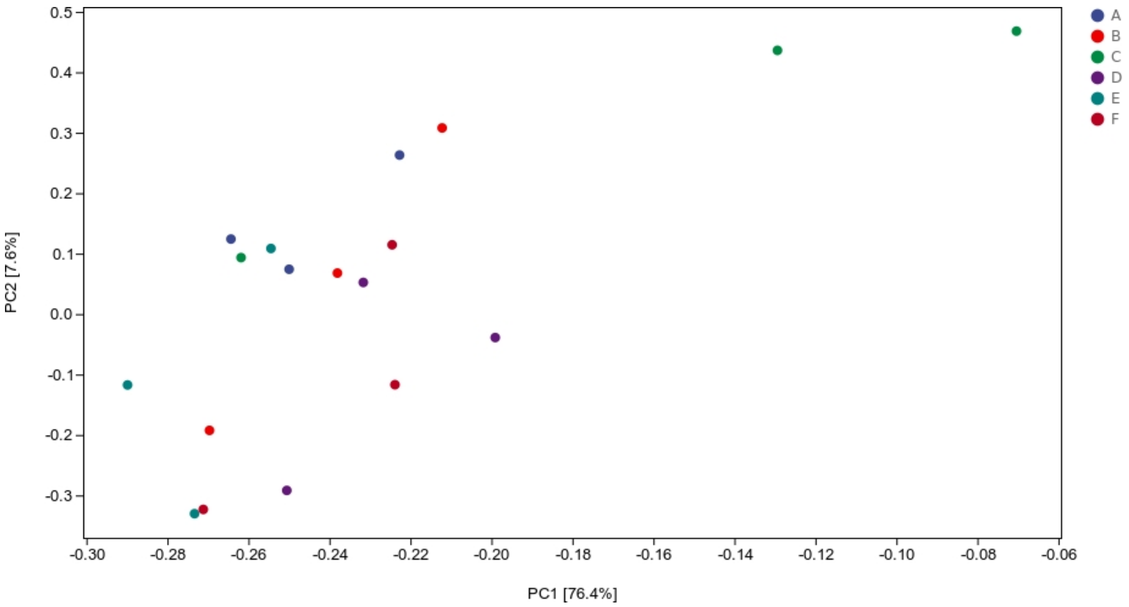

The horizontal coordinate is the first principal component and the vertical coordinate is the second principal component. Different shapes in the figure indicate different samples and different colors indicate different groupings.

2.5 Expression difference analysis

Differential Expression Analysis

We used DESeq to differentially analyze the gene expression and calculated the

differentially expressed ploidy and significance P-value for each gene. Analysis results.

Differential expression analysis table

id	baseMean	baseMean_A	baseMea
BnaC05G0436400ZS	2079.38748579588	18.403268642248	4140.3717
BnaC05G0400000ZS	1446.96880848978	10.8152252451751	2883.12239
BnaA10G0170300ZS	1265.38793372533	11.7132717675425	2519.06259
BnaC05G0526400ZS	1116.16705299878	9.13564310297736	2223.19846

id	baseMean	baseMean_A	baseMea
BnaC05G0500400ZS	865.398323554235	4.7247965098569	1726.07185
BnaC05G0490600ZS	1600.8020571403	22.566977553403	3179.0371
BnaC05G0410200ZS	1171.47649008306	14.1274953935748	2328.82548
BnaC05G0343300ZS	831.693800275747	6.10445139416919	1657.28314
BnaC05G0359600ZS	1379.48822883259	23.3597313003141	2735.61672
BnaC05G0472600ZS	1018.58660649679	6.54506106004232	2030.62815

id: gene number baseMean: the result of homogenization of the read count of the gene for all the samples of the two groups in which the difference is compared baseMean(sample): the result of homogenization of the read count of the gene for all the samples of the group foldChange(Case/Control): fold change of expression differences log2FoldChange: logarithmic value of expression variance versus 2 pval: significance p-value padj: corrected significance p-value

Differential Expression Results Statistics

The results of differential expression analysis were screened for differentially expressed genes with the following conditions: expression differential multiplicity |log2FoldChange|>1, significance P-value<0.05, and the screened set of significantly differentiated genes were counted, and bar charts were made for the differentially expressed genes between different comparative groups, and the number of up-regulated differentially expressed genes and down-regulated differentially expressed genes in each comparative group were counted. number of differential genes in each comparison group.

Analysis results.

Statistical table of differential expression results

Control_vs_Treat	Up-regulated	Down-regulated	Total
F_vs_B	1556	2018	3574
F_vs_C	2272	1780	4052
F_vs_D	2517	3103	5620
F_vs_A	1945	3015	4960
B_vs_D	1551	2154	3705

E_vs_D	1291	1198	2489
F_vs_E	953	1904	2857
Control_vs_Treat	Up-regulated	Down-regulated	Total
C_vs_D	2477	4274	6751
A_vs_D	4051	3825	7876

Control: control sample Treat: experimental sample Up-regulated: Treat number of genes up-regulated compared to Control Down- regulated: Treat number of genes down-regulated compared to Control Total: Treat total number of differentially expressed genes compared to Control

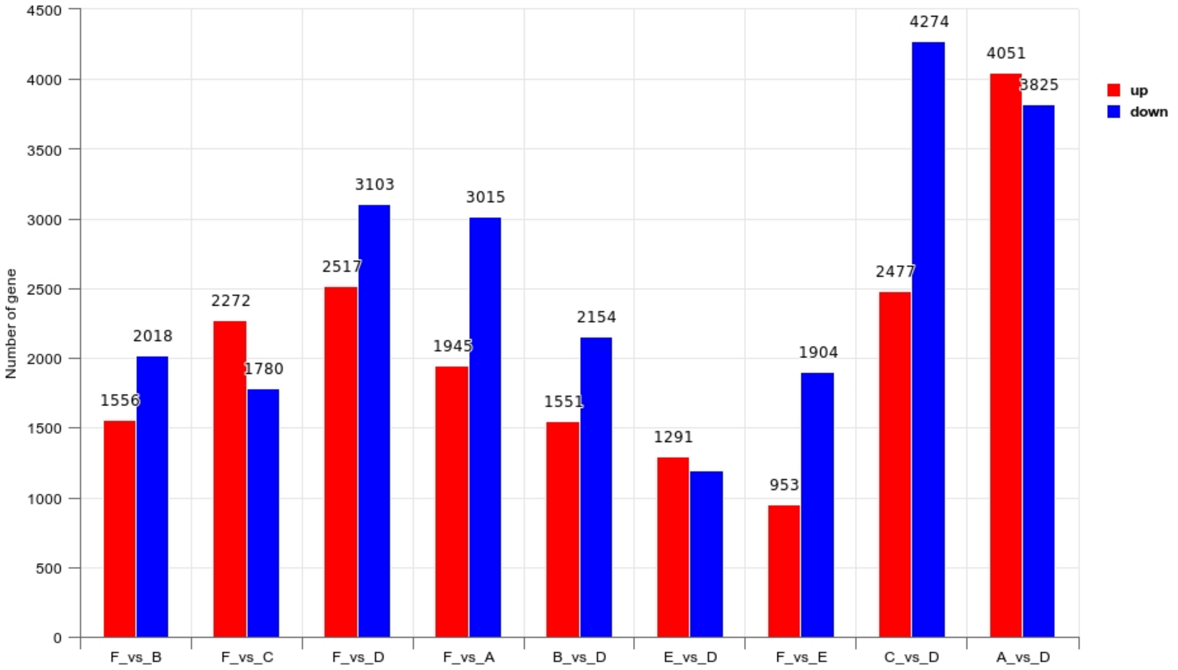

Horizontal coordinates indicate the comparison group for which the analysis of variance was performed, vertical coordinates indicate the number of differentiated genes, and colors indicate up- or down-regulation.

volcanic composition

The R language ggplots2 software package was used to draw the volcano diagram of differentially expressed genes, the volcano diagram demonstrates the gene distribution, the expression fold difference of the genes and the significance results, under normal conditions, the left and right distribution of the differential genes in this diagram should be approximately symmetrical, the left side is the down-regulation of the genes in Case compared with Control, and the right side is the up-regulation of the genes in Case compared with Control.The MA diagram is often used to show the distribution of genes after standardization, generally after standardization, the expression of genes is symmetrically distributed, i.e., the trend of expression difference is not biased with the change of gene expression.

Analysis results.

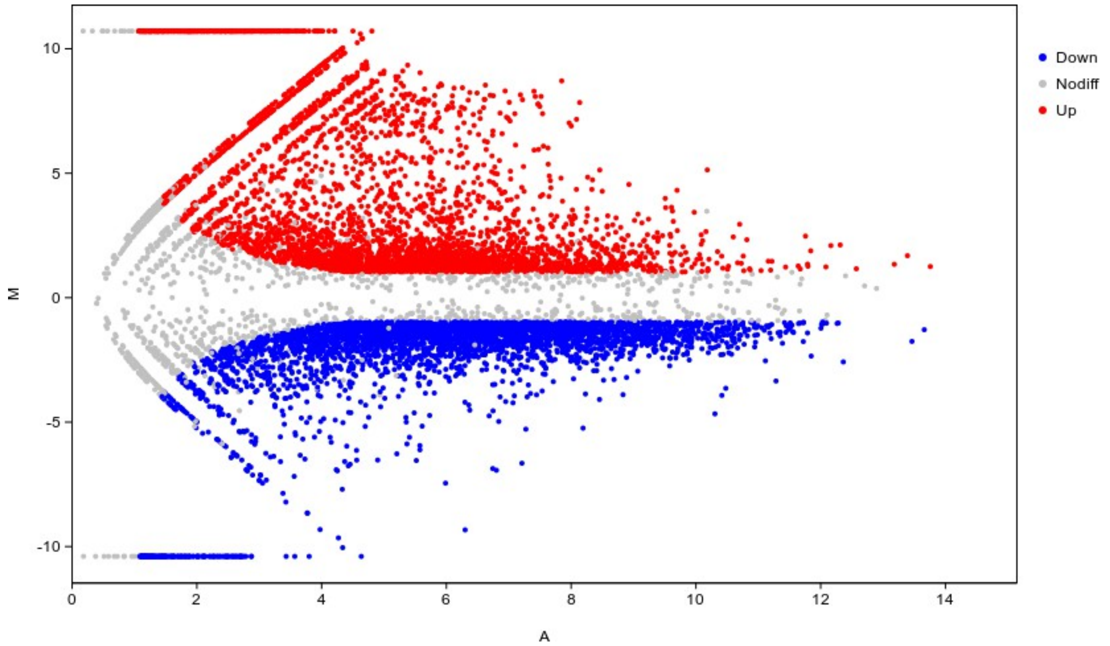

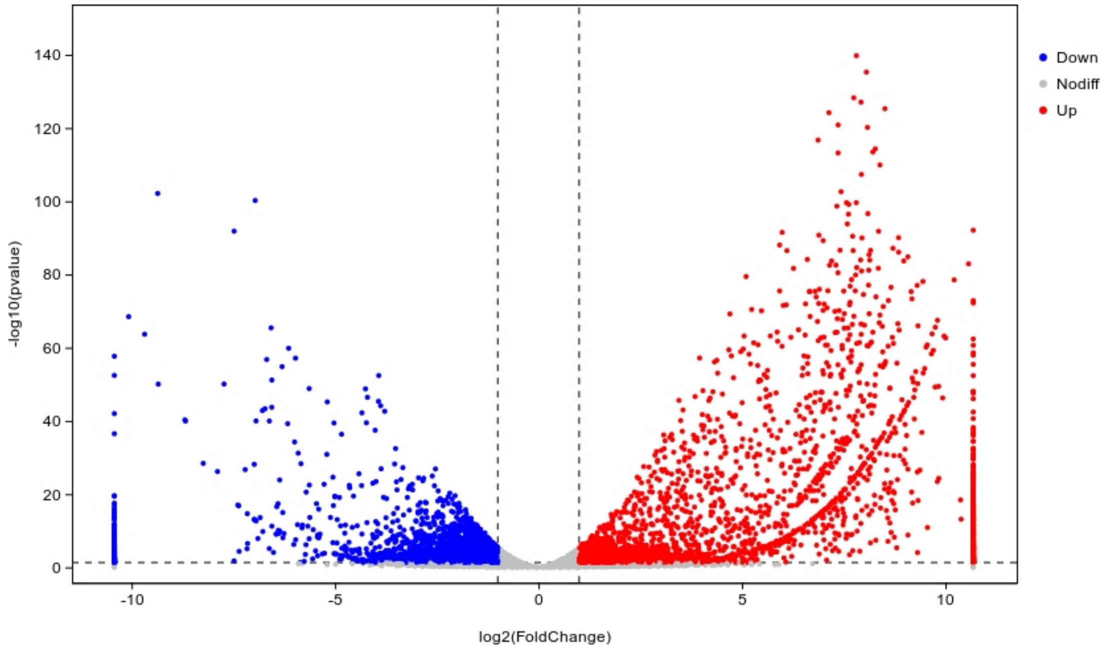

1. Volcano plot: horizontal coordinate is log2FoldChange, vertical coordinate is the significance level taking negative logarithmic values against 10. The two vertical dashed lines in the plot are the thresholds for differential expression multiplicity; the horizontal dashed line is the significance level threshold. The color indicates whether the gene is up-regulated, down-regulated or non-significantly differentially expressed. 2. MA plot: the horizontal coordinate is the logarithmic value of the product of the gene expression of the two samples over 2, i.e., log2(A*B), A and B indicate the expression of the gene in the two samples, respectively, and the vertical coordinate is the logarithmic value of the quotient of expression over 2, i.e., log2(A/B). The color indicates whether the gene is up-regulated, down-regulated or non-significantly differentially expressed.

cluster analysis

Cluster analysis is used to determine the expression patterns of differentially expressed genes under different experimental conditions; genes with high correlation in expression among samples are grouped together, and usually these genes are actually linked in some biological processes, or in some metabolic or signaling pathways. Therefore, through expression clustering, we can discover unknown biological connections between genes.

We used the R language Pheatmap software package to analyze the concatenation of differential genes and samples from all comparison groups in a bidirectional clustering analysis, based on the expression levels of the same gene in different samples and the expression patterns of different genes in the same samples, and clustering by using the Euclidean method to calculate distances, and the hierarchical clustering longest distance method (Complete Linkage).

Analysis results.

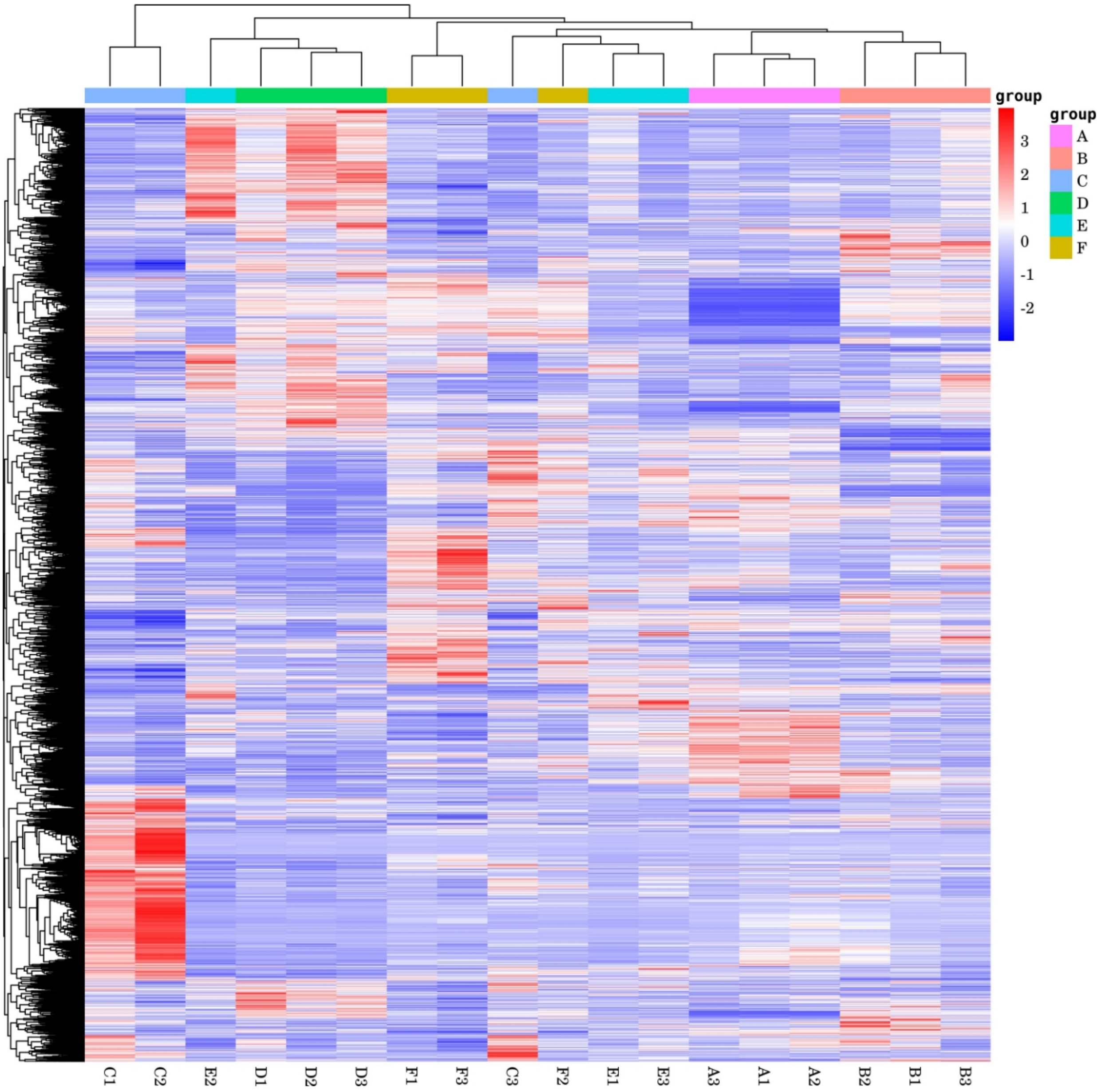

Genes are represented horizontally, one sample per column, with red indicating highly expressed genes and green indicating lowly expressed genes.

Trend analysis

The trend analysis, which is based on the results of the bi-directional clustering heatmap, further divides the genes into different clusters (by default into 9) based on the similarity of

their expression patterns. We consider the genes within each cluster to belong to one class and are more likely to exercise similar functions. The blue trend line of this graph visualizes the change in expression of different types of genes among samples, and thus can be used to narrow down the analysis and focus on key genes.

Analysis results.

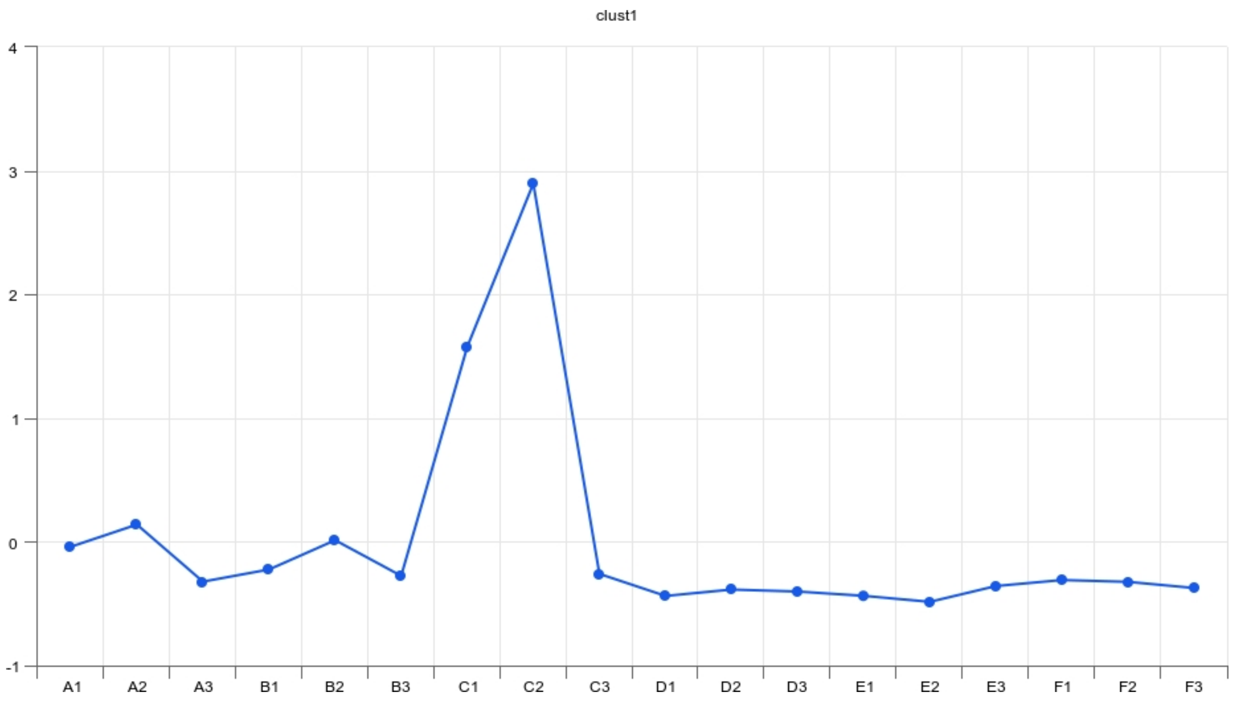

The gray line in the figure demonstrates the expression pattern of the genes in each Cluster, and the blue line indicates the average of the expression of all the genes in the Cluster in the sample.

Differential Gene Wayne Diagram

Based on the results of the difference analysis, the number of shared unique differential genes between the comparison groups was counted. Wayne plots (only Wayne plots for two-, three-, four-, and five-group comparisons were provided) demonstrated the number of differentiated genes between the comparison groups, as well as the overlapping relationships between the comparison groups, Upset plots
(Only matrix plots for more than two comparisons are provided.) The number of differential genes shared between the two-by-two comparison groups is shown. (e.g., source of samples, grouping of experiments), as far as possible, according to their patterns of change to extract the change patterns related to them in the original data, without focusing on other irrelevant data information. For example, LDA analysis used in LEfSe analysis is a supervised pattern recognition method based on supervision. (e.g. source of samples, grouping of experiments), as far as possible in accordance with their change rules to extract the original data in the change pattern related to it, without focusing on other irrelevant data information. For example, the LDA analysis used in LEfSe analysis is a supervised pattern recognition based method.

Analysis results.

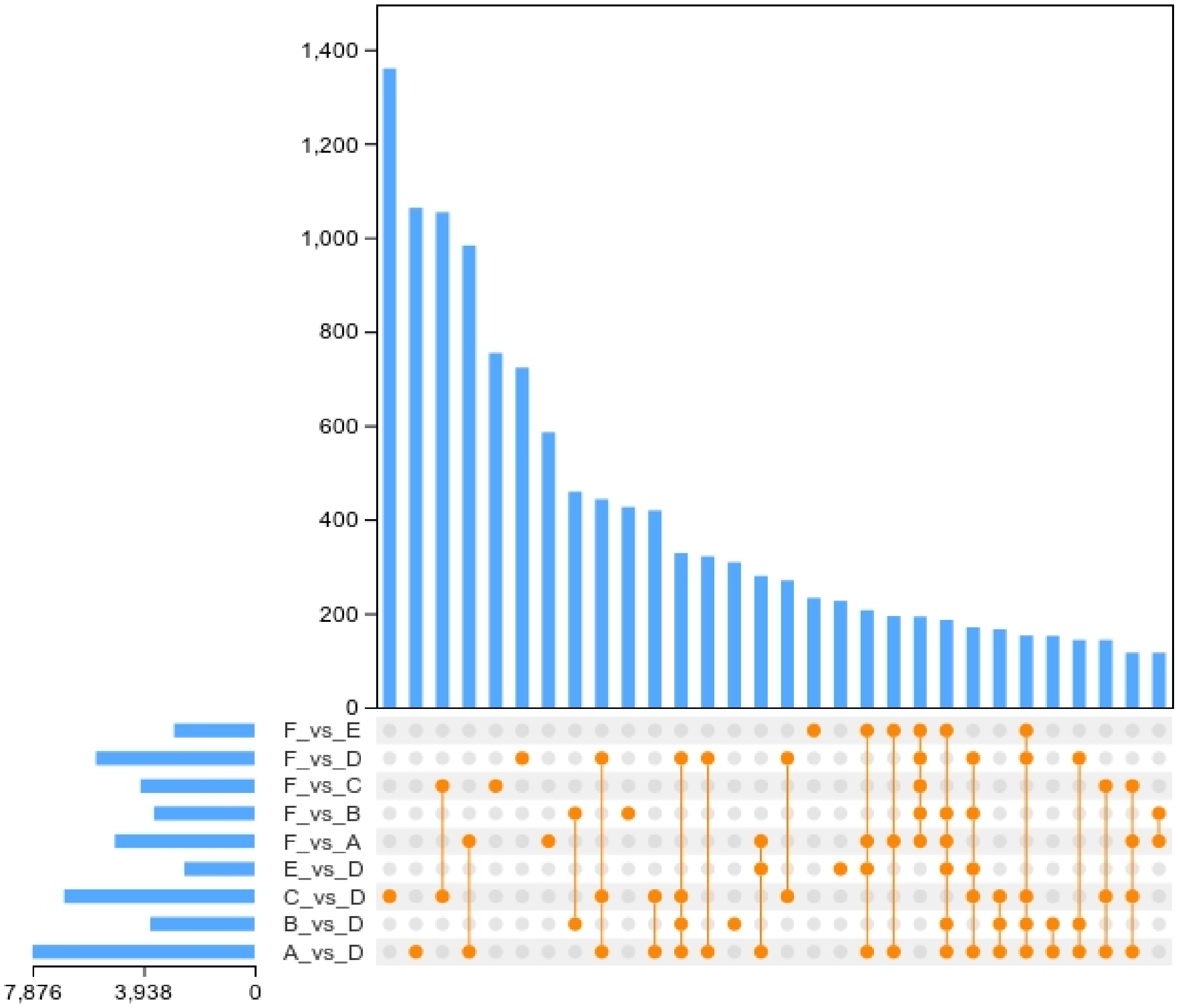

1,400
1,200
1,000
800
600
400
200
0
F_vs_E
F_vs_D
F_vs_C
F_vs_B
F_vs_A
E_vs_D
C_vs_D
B_vs_D
A_vs_D
7,876
3,938
0

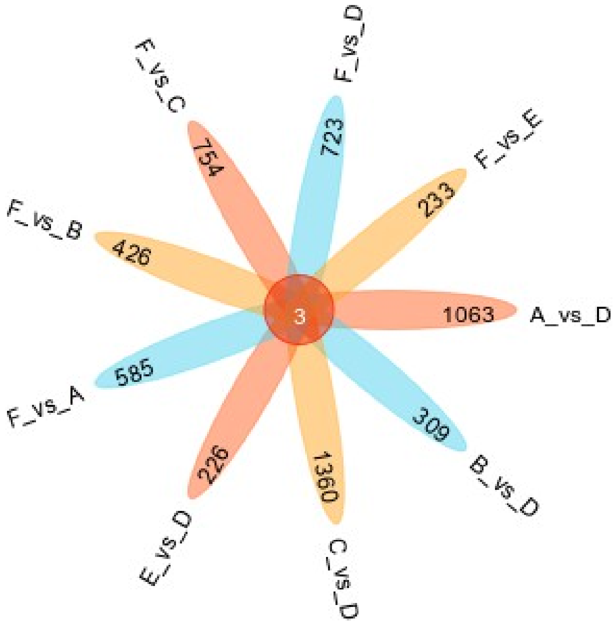
F_vs_D
723
F_vs_C
754
F_vs_E
233
F_vs_B
426
3
1063
A_vs_D
585
F_vs_A
309
B_vs_D
226
E_vs_D
1360
C_vs_D

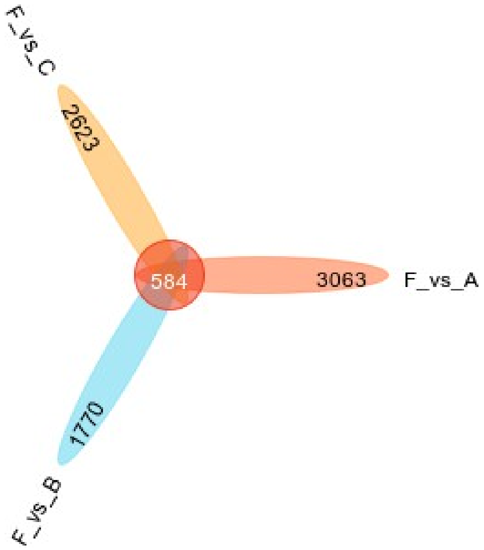

1. Upset chart: number in each set represents the number of all differential genes identified in each comparison group, number of each intersection represents the number of shared differential genes identified in multiple comparison groups, a point in the horizontal coordinate represents the number of unique differential genes identified in the comparison group, and a line connecting multiple points in the horizontal coordinate represents the number of shared differential genes identified in multiple comparison groups in the connecting line. Venn diagram: the sum of the numbers in each circle represents the total number of differential genes in the comparison group, and the overlapping part of the circle represents the differential genes shared between two comparison groups.

genome circle diagram

Using the R language Circlize package, genomic circle maps are created by marking differentially expressed RNAs on the genome based on genomic information and RNA differential expression analysis results, which can reflect the distribution of differentially expressed genes on chromosomes between different comparative groups.

Protein Network Interaction Analysis

Protein Interaction Network Analysis, abbreviated as PPI analysis, is an analysis that reveals the interactions between genes. The analysis uses the STRING database for the prediction of interactions.STRING database (Search3 Tool for theRetrieval of Interacting Genes/Proteins) is a protein interactions database developed by EMBL, https://string-db.org/cgi/ input.pl, which contains protein interactions from the strongest experimental evidence to data mining and homology prediction.

PPI analysis allows the exploration of interactions among the target gene set, screening key genes from the gene set to further narrow down the target, and is an important component of data mining. We performed protein interactions analysis based on the STRING database as a way to reveal the role relationships between target genes. When the PPI information of the

species was included in the STRING database, we screened the PPI interaction pairs containing differential genes and with Score>0.95 in the direct database based on the results of gene differential expression analysis. The Score value can be adjusted when the network is too large or too small.

When there is no PPI information for the species in the STRING database, we select the similar species to compare with the protein sequences of the species, and then get the interrelationship between the proteins of the species. Then we screened the PPI pairs that contained differential genes and had Score>0.95. Finally, we obtained the interrelationships between all the target genes (*PPI.network.txt), which were plotted by Cytoscape.

Analysis results.

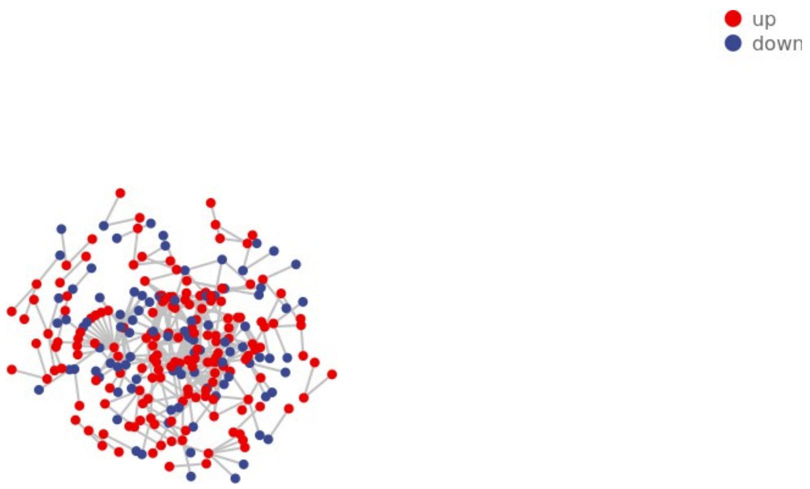

The figure shows a network diagram of protein interaction networks with expression binding, where the dots are genes (corresponding proteins), where up indicates up-regulated genes and down indicates down-regulated genes

Analysis of exon differences

The DEXSeq package is used to analyze RNA-seq experimental data for exonusage differences, where exonusage differences refer to differences in exon usage due to experimental conditions.DEXSeq internally calls the DESeq2 software to perform the analysis, which is based on the same principles as the DESeq2 software.

The basic idea of PDEU (differentialexon usage) is that, for each exon (or part of exon) of each sample, we count the number of reads that are compared to that exon and the number of reads that are compared to other exons of the same gene (which contains multiple transcripts), and then we calculate the ratio of these two statistics, and then we finally deduce the relative exon usage change based on the change of the ratio under different experimental conditions. , the relative exon usage change was deduced. For an exon within a gene, this exon usage is synchronized to the proportion of that exon that is spliced into the transcriptome (variable splicing), which also incorporates variable splicing that occurs at the 5' and 3' ends of the transcript that can lead to differential exon usage at transcript boundaries. Thus, the DEU
(Bydifferential exon usage) is more intuitive compared to variable splicing. >0.95 PPI action pairs. Score value can be adjusted when the network is too large or too small.

> Analysis results.

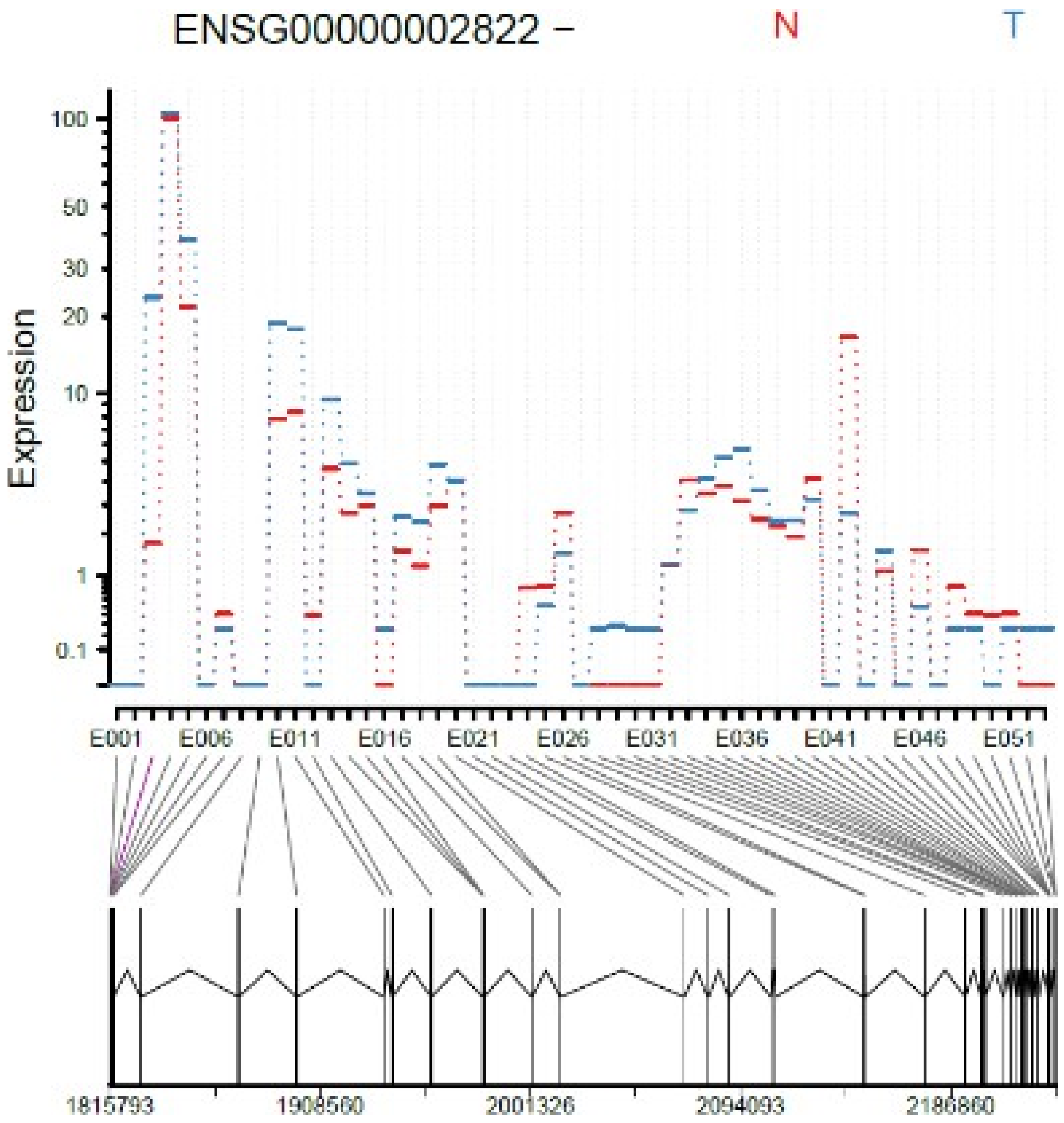

Horizontal coordinates are mean expression, vertical coordinates are log2FoldChange, and significant differences (padj<0.05) are shown in red Results

2.6 enrichment analysis

GO enrichment analysis

A database established by the Gene Ontology Consortium (Gene Ontology, http://geneontology.org/).GO was created primarily to address the confusion in the definition of the same gene in different databases as well as the confusion in the definition of the function of the same gene in different species. It is an internationally standardized gene function classification system that provides a set of dynamically updated standard vocabulary (Controlled Vocabulary) to comprehensively describe the attributes of genes and gene products in living organisms.GO covers three aspects, which are describing the molecular function of genes (Molecular Function), the role of cellular components

(Cellular Component, and Biological Processes Involved.
(Biological Process.) Genes or proteins can be assigned a GO number by ID correspondence or sequence annotation, and the GO number can be used to correspond to a GO Term, i.e., a functional class or cellular localization. The basic unit of a GO is a Term, each Term has a unique identifier (consisting of "GO:" plus 7 numbers, e.g., GO:0072669); each Ontology class has a GO number (e.g., GO:0072669) and a GO number (e.g., GO:0072669).

of Term form a directed acyclic topology by means of the links (is_a, part_of, regulate) between them.GOSlim is a reduced version of the GO terminology, which provides an overview of the results of the GO annotations.

We performed GO enrichment analysis using topGO, which uses GO term annotated differential genes for each term's gene list and gene number, and then calculates the P-value by hypergeometric distribution method (the criterion for significant enrichment is P-value<0.05) to find out the GOs that are significantly enriched in differential genes compared to the whole genomic background of the term , thus identifying the major biological functions exercised by the differential genes.

Analysis results.

Go enrichment analysis table

GO.ID	Category	Term	Up
GO:0010200	BP	response to chitin	13
GO:0006952	BP	Defense response	198
GO:0010243	BP	response to organonitrogen compound	39
GO:0005576	CC	extracellular region	399
GO:0042493	BP	response to drug	91
GO:1901700	BP	response to oxygen-containing compound	309
GO:0030312	CC	external encapsulating structure	262
GO:0005618	CC	cell wall	261
GO:0006950	BP	response to stress	541
GO:0051707	BP	response to other organisms	178

GO.ID: GO number Catergory: classification in which GO Term is located GO_Term: GO entry Up/Down: up/down-regulated genes enriched to this GO entry Total: total number of genes enriched to the changed GO entry Pvalue: enrichment significance P-value adjustPvalue: P-value correction value

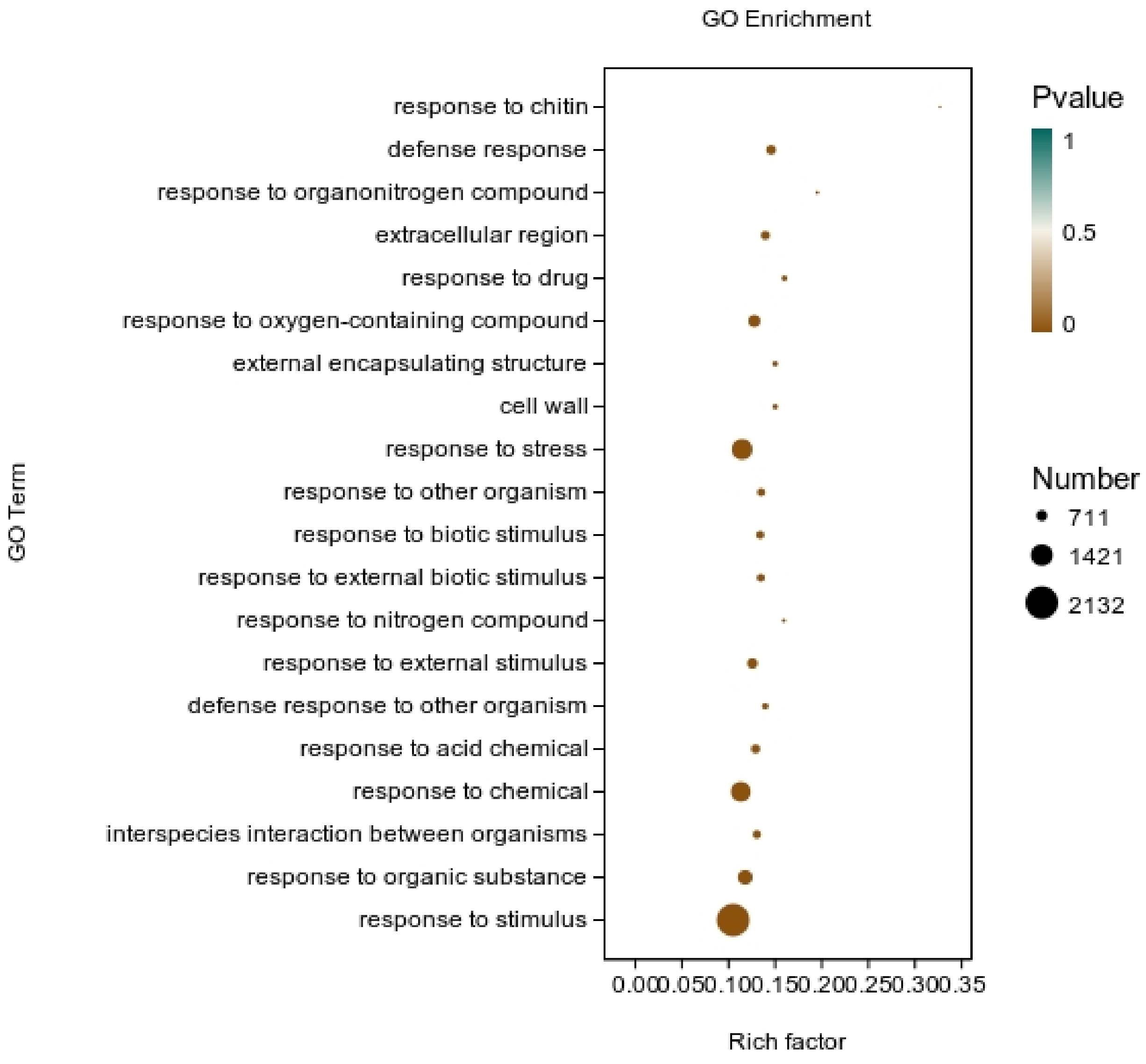

GO Enrichment
response to chitin
defense response
response to organonitrogen compound
extracellular region
response to drug
response to oxygen-containing compound
external encapsulating structure
cell wall
response to stress
response to other organism
response to biotic stimulus
response to external biotic stimulus
response to nitrogen compound
response to external stimulus
defense response to other organism
response to acid chemical
response to chemical
interspecies interaction between organisms
response to organic substance
response to stimulus
GO Term
0.000.050.100.150.200.250.300.35
Rich factor
Pvalue
1
0.5
0
Number
711
1421
2132

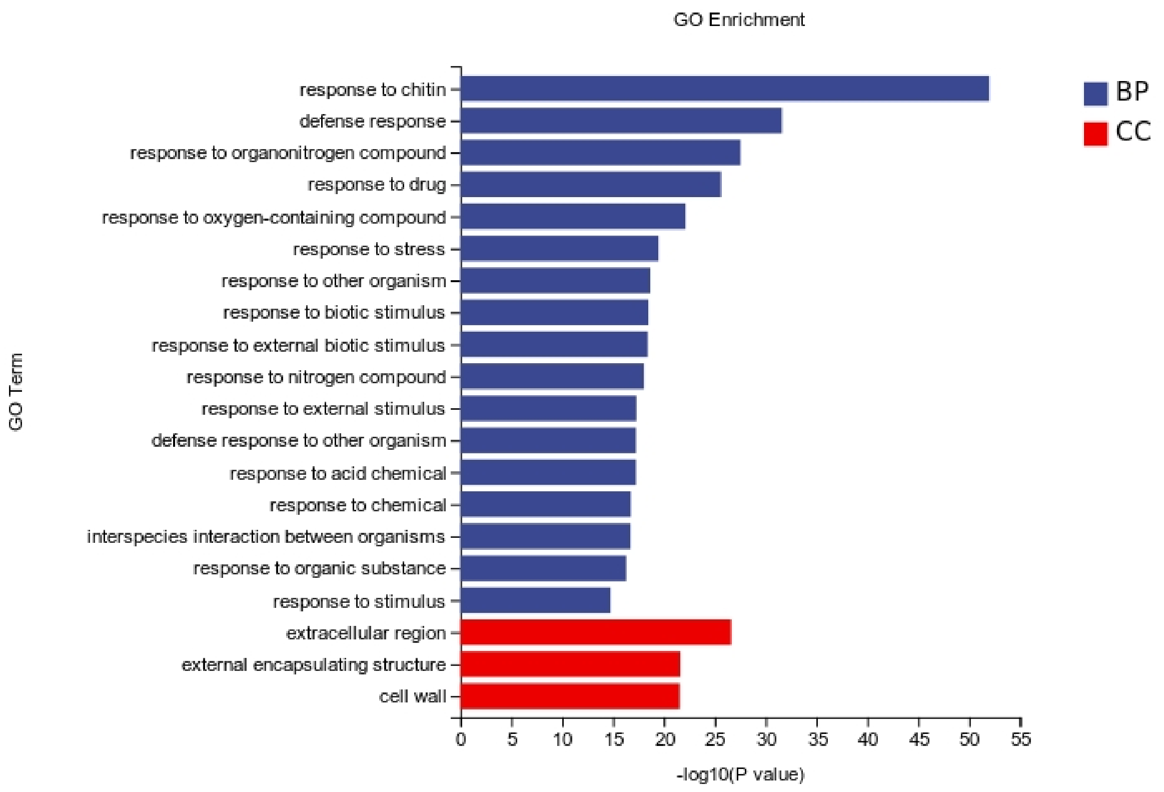

Bar chart: horizontal coordinate is GO Term, vertical coordinate is GO Term enriched -log10 (p-value) factor plot: horizontal coordinate is rich factor (number of differential genes annotated to the GO Term/total number of genes annotated to that GO Term), vertical coordinate is GO Term, the size of the dots in the plot indicates the number of differentials (up- or down-regulated) genes annotated to the corresponding term, and the color of the dots indicates the level of significance. The size of the dots in the graph indicates the number of differential (up- or down-regulated, depending on the gene set selected for analysis) genes annotated in the corresponding term, and the color indicates the level of significance.

KEGG enrichment analysis

Kyoto Encyclopedia of Genes and Genomes (KEGG), http:// www.kegg.jp/是一个整合了基因组, a database of chemical and systemic functional information. One of the features of the KEGG database is the association of gene catalogs derived from fully sequenced genomes with higher-level system functions at the cellular, species, and ecosystem levels.KEGG annotations include: (1) KO (KEGG Ortholog) annotations, which are cross-species annotations of molecular networks; (2) KEGG Pathway annotations, which are metabolic pathway annotations, which obtain interactions between molecules within a species and metabolic pathways; (3) KO annotations, which are metabolic pathway annotations. KEGG Pathway annotation, i.e., metabolic pathway annotation, to obtain the network of molecular interactions and reactions within a species.

We performed KEGG enrichment analysis using clusterprofiler, in which the list of genes and the number of genes per pathway were calculated using the KEGG pathway annotated differential genes, and then the P-value was calculated by the hypergeometric distribution method (the criterion for significant enrichment was P-value<0.05) to identify the KEGG pathways that were significantly enriched in KEGG pathways compared to the whole P-value (P-value<0.05 was used as the criterion for significant enrichment) was then calculated by the hypergeometric distribution method to identify KEGG pathways that

were significantly enriched for differential genes compared to the whole genomic background, thus determining the major biological functions exercised by the differential genes.

Analysis results.

KEGG enrichment analysis table

PathwayID	Pathway	lev
bna00940	Phenylpropanoid biosynthesis	Metab
bna00040	Pentose and glucuronate interconversions	Metab
bna04075	Plant hormone signal transduction	Environmental Infor
bna00520	Amino sugar and nucleotide sugar metabolism	Metab
bna00906	Carotenoid biosynthesis	Metab
bna00920	Sulfur metabolism	Metab
bna00604	Glycosphingolipid biosynthesis - ganglio series	Metab
bna00270	Cysteine and methionine metabolism	Metab
bna04626	Plant-pathogen interaction	Organisma
bna00900	Terpenoid backbone biosynthesis	Metab

PathwayID: KEGG Pathway number Pathway: Pathway description level1/level2: Classification of the Pathway at level1 or level2 Up/Down: Enrichment of up/down-regulated genes to the Pathway entry Total: Total number of genes enriched to the changed Pathway entry. Pvalue: P-value of enrichment significance adjustPvalue: P-value correction value

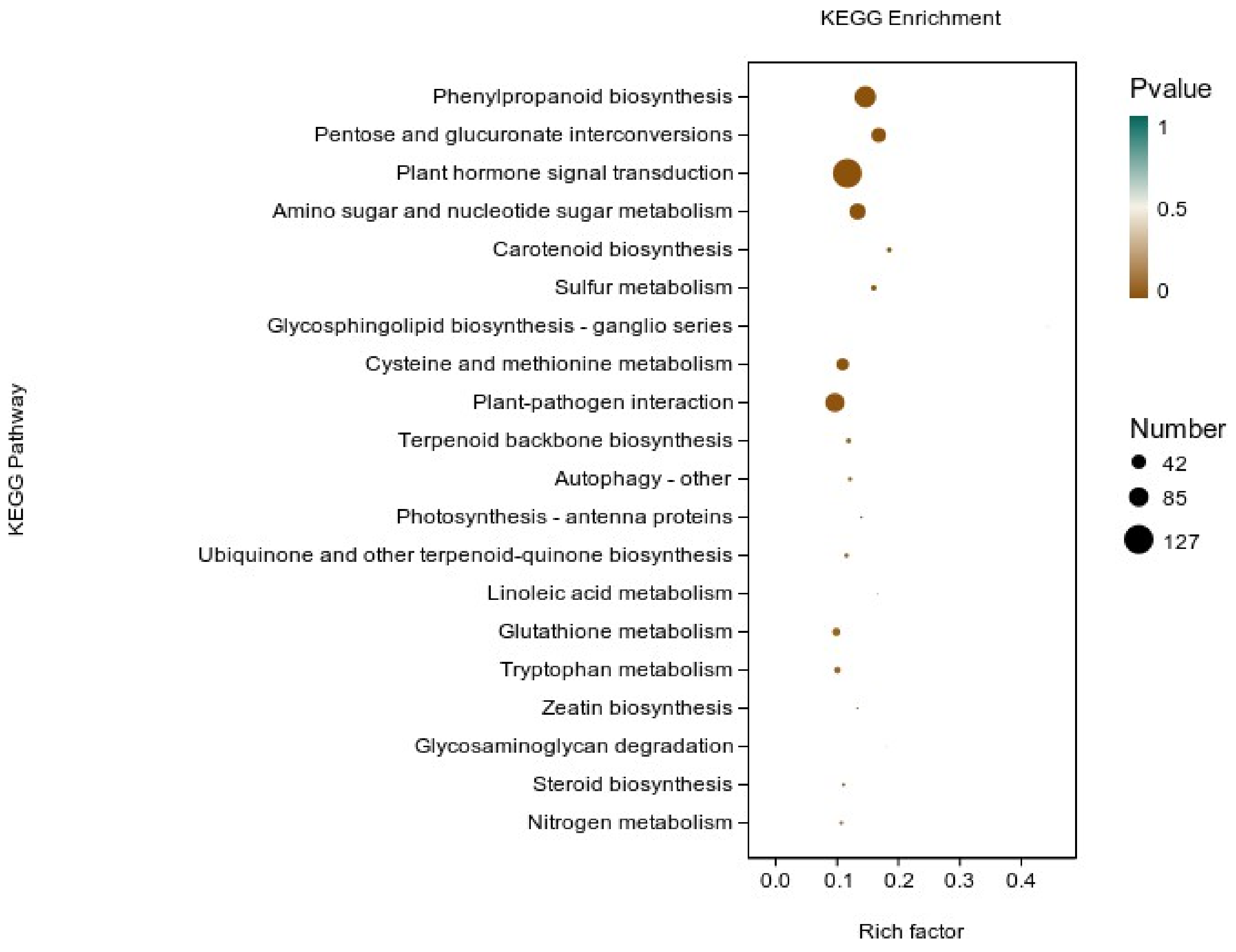

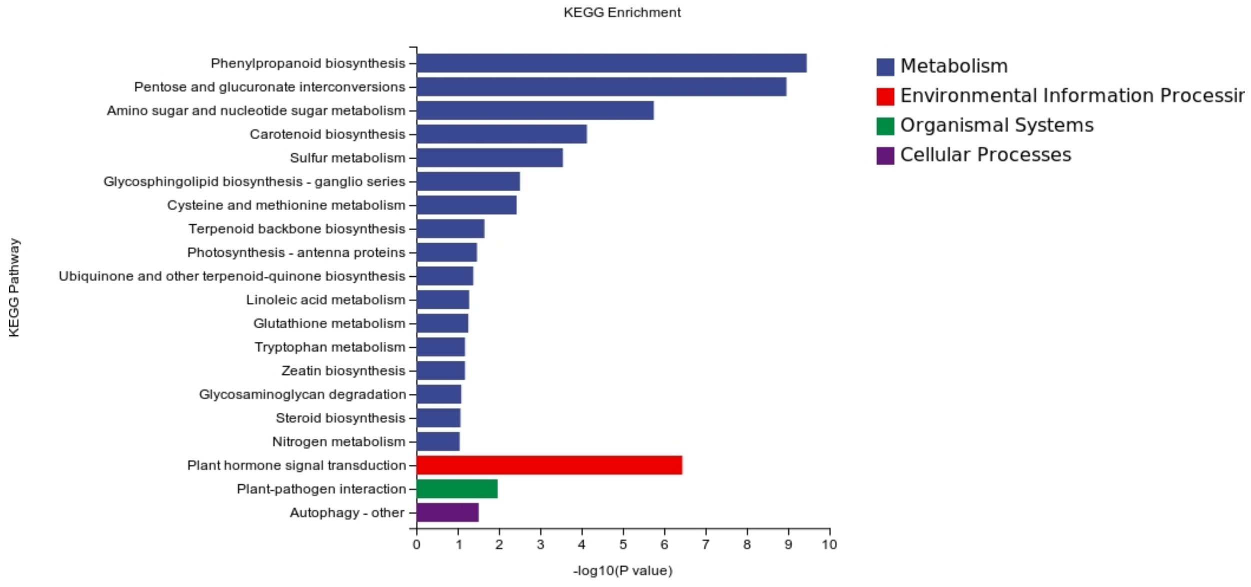

Bar chart: horizontal coordinate is Pathway, vertical coordinate is Pathway enriched -log10 (p-value) factor plot: horizontal coordinate is rich factor (number of differential genes annotated to the Pathway/total number of genes annotated to the Pathway), vertical coordinate is Pathway, and the size of the dots in the plot indicates the number of differential (up- or down-regulated) genes annotated to the corresponding Pathway, and the color shade indicates the level of significance. The size of the dots in the plot indicates the number of differentially (up- or down-regulated, depending on the gene set selected for analysis) annotated genes in the corresponding Pathway, and the shade of the color indicates the level of

significance.

2.7 structural analysis

transcript splicing

Transcripts are one or more mature mRNAs that can code for proteins formed by transcription of genes.For the results of second-generation transcriptome sequencing, based on the presence or absence of the reference genome, there are two main ways of transcript assembly: mapping-based assembly and ab initio assembly.Based on the existing reference genome, we chose the mapping-based assembly method and assembled and spliced the mapped reads by using the software StringTie (http://ccb.jhu.edu/software/stringtie/) to assemble the mapped reads for splicing.

New transcript analysis

We compared the spliced transcript sequences with the known transcripts to obtain the transcripts without annotation information, and the transcripts with Class Code "j", "i", "u" were treated as new transcripts, and "x" might be the antisense transcript of the known transcripts, so it was also treated as a new transcript, and all the new transcripts were functionally annotated.

Class Code is a description of the position of the spliced transcript in relation to known

genes and transcripts given by Stringtie as shown in the following table:Analysis Results.

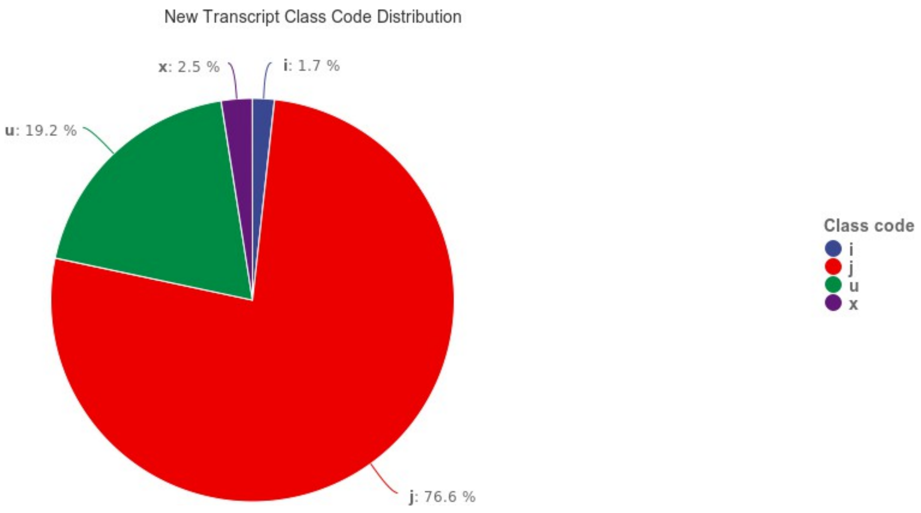

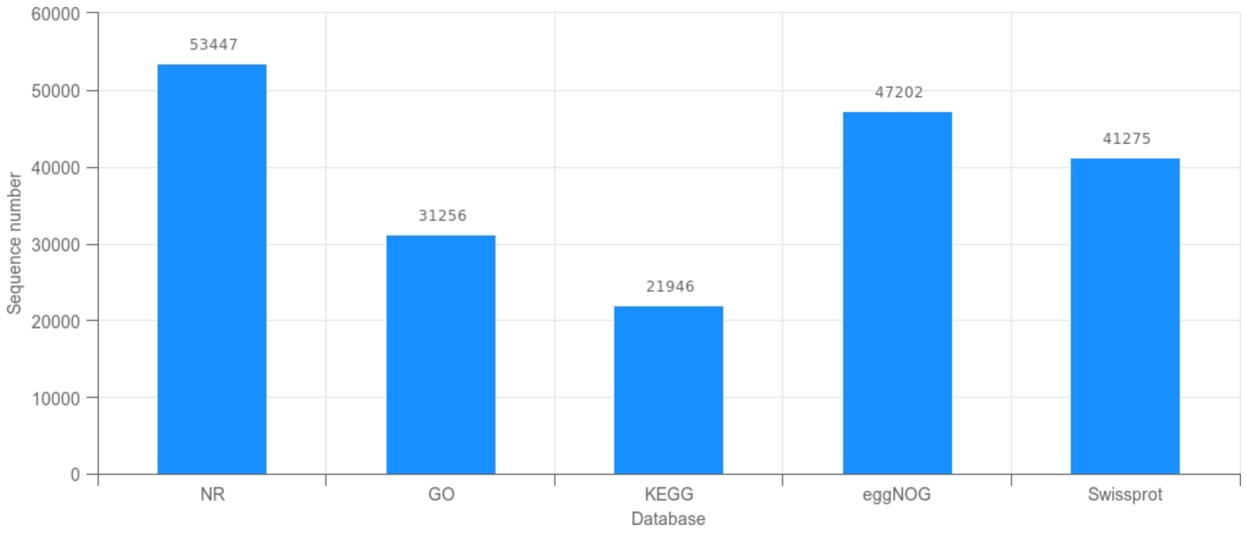

1. pie chart: each area of the chart is the proportion of Class_code; 2. bar chart: the horizontal coordinate is the individual databases and the vertical coordinate is the number of new transcripts annotated.

UTR Optimization Analysis

The functional units obtained can be used to obtain the abundance values of metabolic pathways based on metabolic pathway databases and certain calculation methods.KEGG databases, MetaCyc data, and COG data are commonly used databases.

Differential Variable Shear Analysis

Alternative Splicing (AS) refers to the generation of different mRNA splicing isoforms from one mRNA precursor of some genes by different splicing modes (selection of different splice sites). It is a very common mode of gene expression in eukaryotes and is important for regulating gene expression and generating proteomic diversity. Variable shearing studies can be divided into two levels: 1. the prediction of variable shearing isoforms (qualitative); and 2. the study of changes in the relative expression of variable shearing isoforms (quantitative), the latter of which tends to be the focus of practical research.

Differential variable shearing refers to the quantification of transcripts generated by variable shearing based on the identification of variable shearing events, followed by differential analysis based on a comparative grouping approach to identify whether differential variable shearing occurs at a certain shear site in two groups of samples. In summary, differential variable shear analysis consists of the following four steps: 1) transcript assembly; 2) identification of variable shear events; 3) quantification of variable shear transcripts; and 4) differential analysis.

We used rMATS (http://rnaseq-mats.sourceforge.net/index.html) software for differential variable shear analysis. The software recognizes five types of variable shear events: skipped exon (SE) skipped exon, alternative 5′ splice site (A5SS) exon 3′ shear site variable (i.e., the 5′ shear site of the intron that follows it is variable), alternative 3′ splice site (A3SS) exon 5′ variable shear (i.e., the 3′ shear site of its preceding intron is variable), mutually exclusive exons (MXE) mutually exclusive exons, and retained intron (RI) retained introns. The diagram is shown below:

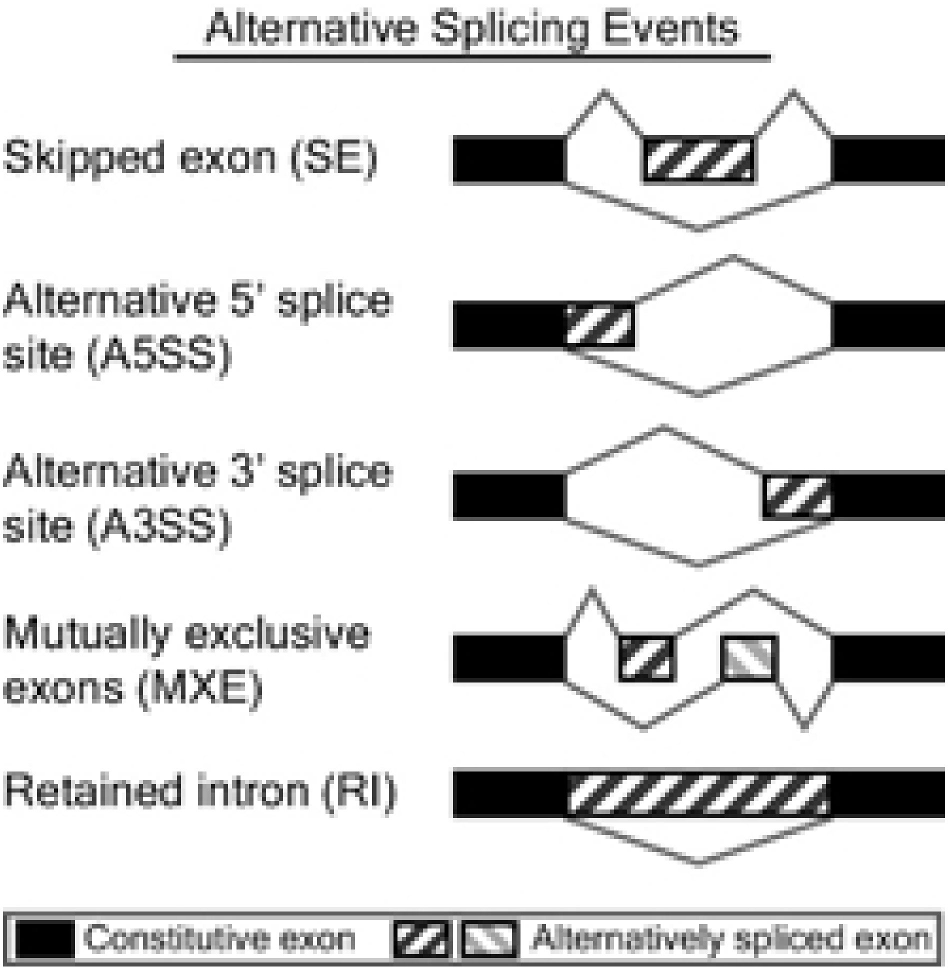

There are two ways to quantify rMATS, Junction counts, which only use Reads that span the splice site, and Reads On Target And Junction Counts, which take into account all Reads that are compared to the spliced fragments. in general, comparing two sets of samples with differential variable splicing requires only the Junction counts results. Here we give the results of Junction counts. The statistical principle of rMATS is ф = (I/LI) / (I/LI + S/LS).

ф (exoninclusion level) was used to quantify variable shear, i.e., the percentage of transcripts containing variable shear event regions among those containing and skipping variable shear event regions. The Δф (Δф = | ф1- ф2 |) and FDR values were used to confirm the presence of differential variable shear between the two groups of samples. ф1 and ф2 are the exon inclusion level of the two groups of samples, respectively, and the exon inclusion level of the two groups of samples was determined when Δф >5% was met and the FDR was determined.

At <=1%, the two groups of samples were considered to have undergone differential variable shear at that shear site.

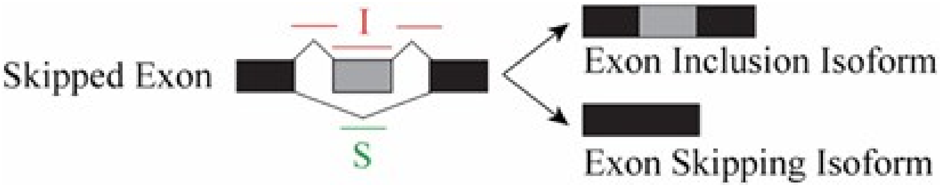

Analysis results.

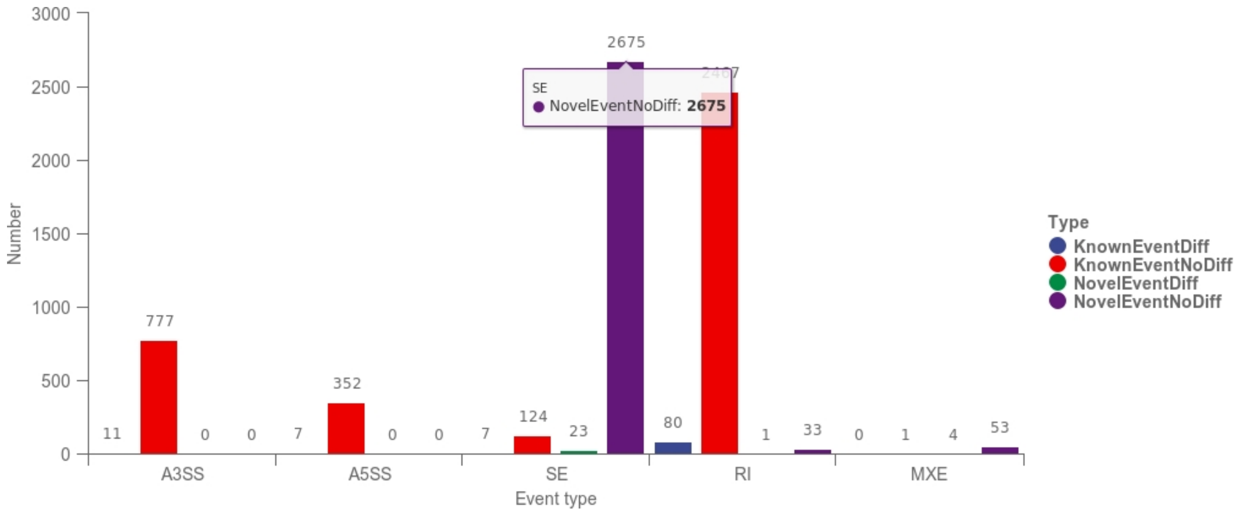

KnownEventDiff is a known transcript undergoing variable shear KnownEventNoDiff is a known transcript not undergoing variable shear NovelEventDiff is a new transcript undergoing variable shear NovelEventNoDiff is a new transcript not undergoing variable shear Horizontal coordinates show the five forms of variable shear recognized by rMATS. Vertical coordinates show the number of transcripts

2.8 variation detection

SNP

SNPs (SingleNucleotide Polymorphisms) are genetic markers formed by single nucleotide variations in the genome, which are numerous and rich in polymorphisms.SNPs appear most frequently in CG sequences, and most of them are the conversion of C to T. The reason is that C is often methylated in CG, and is spontaneously deaminated to thymine.There are a number of reasons for the appearance of SNPs. SNPs can occur for a variety of reasons, including single nucleotide polymorphisms in the genetic background, mutations caused by library construction techniques, or read errors in sequencing. SNPs in the transcriptome are cSNPs, referring to SNPs that occur in the coding region.

SNPs can have an effect on the translation of genes, and the distribution of various types of codon mutations in each sample was counted, including: frameshift substitution: codon shift substitution; nonframeshift substitution: nonsynonymous substitution; nonsynonymous SNV: nonsynonymous single nucleotide mutation; stopgain: stop codon increase; stoploss: stop codon decrease; synonymous SNV: synonymous single nucleotide mutation; unknown: unknown type.

The Varscan program acquires SNP loci with filtering criteria:

1) SNP site base Q >20;

2) The number of Reads covering the locus is >8;

3) The number of Reads supporting the mutated locus is >2;

4) SNP loci with p-value <

0.01. Analysis results.

SNP statistics table

Chr	Pos	Ref	Alt	GeneID	Gene
scaffoldA03	670022	C	G	BnaA03G0014000ZS (dist=85)	-
scaffoldA03	670023	C	T	BnaA03G0014000ZS (dist=84)	-
scaffoldA03	670025	A	C	BnaA03G0014000ZS (dist=82)	-
scaffoldA03	670026	A	T	BnaA03G0014000ZS (dist=81)	-
scaffoldA03	670027	A	C	BnaA03G0014000ZS(dist=80)	-
scaffoldA03	1179413	A	C	BnaA03G0025300ZS	-
scaffoldA03	1179747	T	C	BnaA03G0025300ZS	-
scaffoldA03	1180273	T	C	BnaA03G0025300ZS	-
scaffoldA03	1180509	T	G	BnaA03G0025300ZS	-
scaffoldA03	1722959	C	T	BnaA03G0037500ZS	-

CHROM: chromosome on which the SNP locus is located POS: position of the SNP locus on the chromosome REF/ALT: genotype/mutant genotype of the reference sequence at the locus Columns under the sample name: number of Reads supporting each genotype

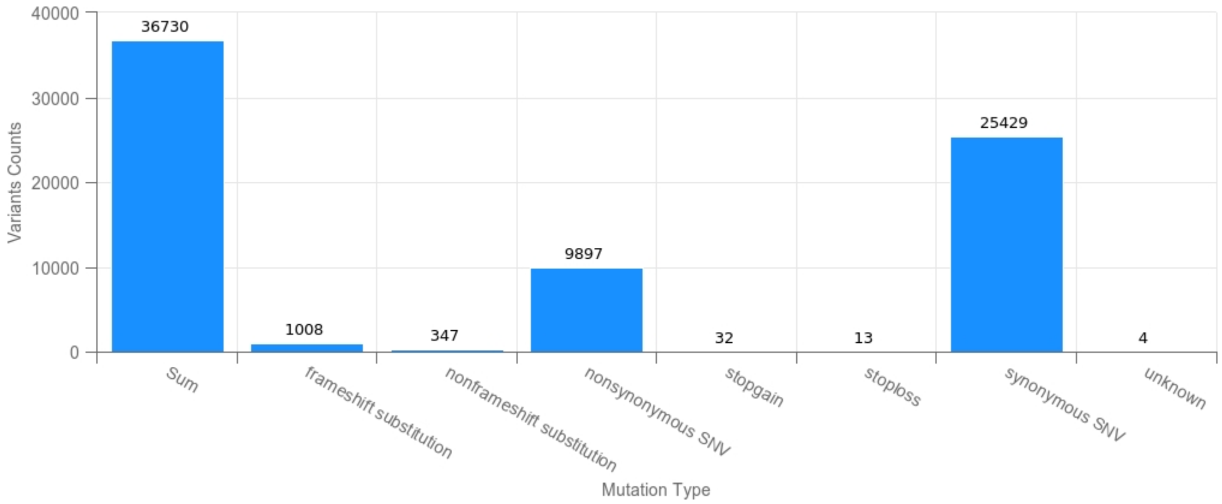

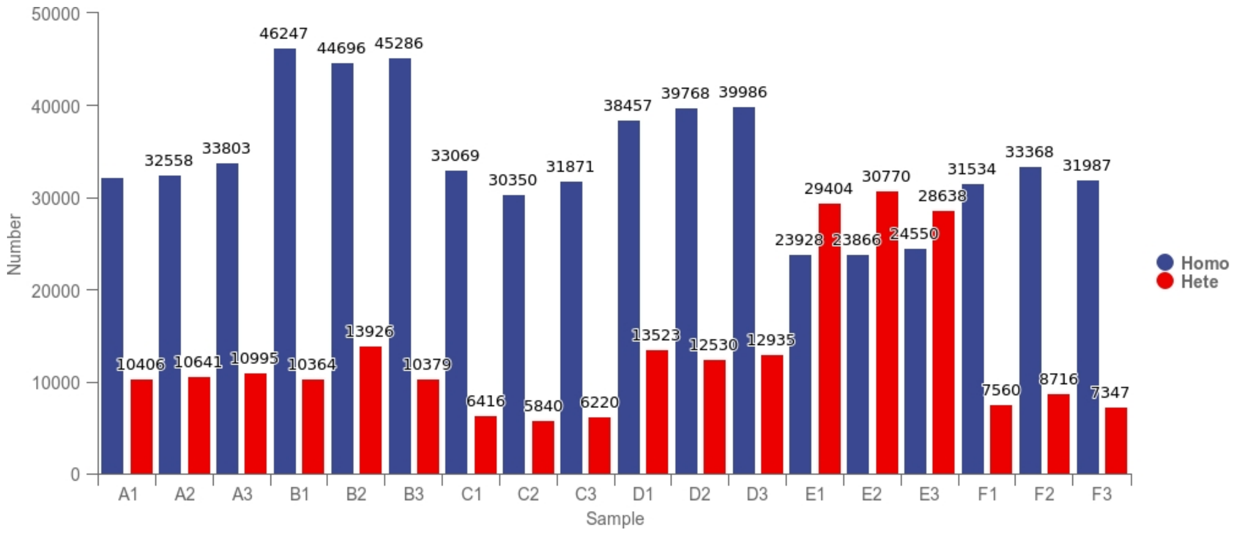

Homo (homozygous - variant) denotes a pure mutant, i.e., the alleles at this locus are all mutated and the mutations are identical, Hete
(heterozygous-variant) denotes a heterozygous variant, i.e., at least one of the alleles at this locus is mutated and the mutations are allele different. frameshift substitution: codeshift substitution; nonframeshift substitution: nonshift substitution; nonsynonymous SNV: nonsynonymous single-nucleotide mutation; stopgain: stop codon gain; stoploss: stop codon loss; synonymous SNV: synonymous single nucleotide mutation; unknown: unknown type.

InDel

InDel (Insertion-Deletion) refers to an insertion or deletion of a small segment of a sample relative to a reference genome, which may contain one or more bases.InDel can be used as a genetic marker to study phylogeny or to identify a species.InDel may cause a shifted mutation, resulting in an incorrect termination codon during mRNA translation. InDel may cause a shifted mutation that results in an incorrect stop codon during mRNA translation. Generally, InDels that are not multiples of 3 occur infrequently in coding regions and relatively frequently in non-coding regions. Except in the vicinity of highly repetitive regions, InDel generally occurs less frequently than SNPs.

The Varscan program acquires InDel sites with

the following filtering criteria: 1) InDel site base

Q>20;

2) The number of Reads covering the locus is >8;

3) Number of Reads supporting

the mutation site > 2; 4) p-value

< 0.01 for the InDel site. analysis

results.

InDel Statistical Tables

Chr	Pos	Ref	Alt	GeneID
scaffoldA03	68281	C	CT	BnaA03G000140
scaffoldA03	115328	C	CT	BnaA03G0002700ZS (
Chr	Pos	Ref	Alt	GeneID
scaffoldA03	128005	G	GT	BnaA03G000300
scaffoldA03	152018	AC	A	BnaA03G000360
scaffoldA03	178444	G	GA	BnaA03G000420
scaffoldA03	238199	GA	G	BnaA03G000480
scaffoldA03	290839	TA	TAA,T	BnaA03G0005900ZS(dist=3287),BnaA
scaffoldA03	318714	A	AT	BnaA03G0006400ZS
scaffoldA03	335493	G	GT	BnaA03G0006700ZS (
scaffoldA03	341782	TA	T,TAA	BnaA03G000690

CHROM: chromosome on which the SNP locus is located POS: position of the SNP locus on the chromosome REF/ALT: genotype/mutant genotype of the reference sequence at the locus Columns under the sample name: number of Reads supporting each genotype

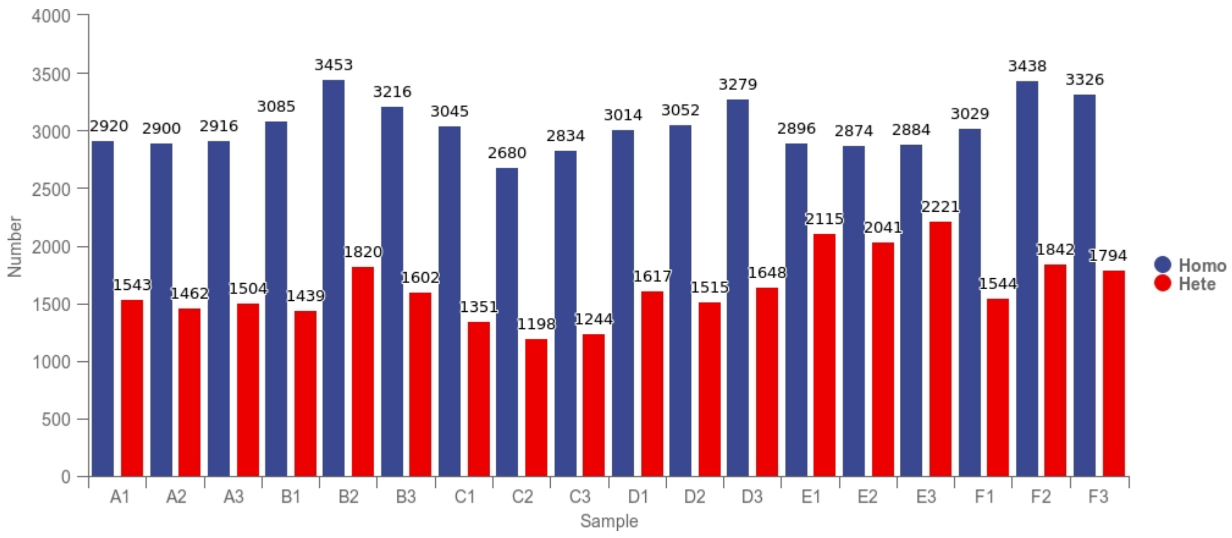

Homo (homozygous-variant) denotes a pure mutant, i.e., the alleles at this locus are all mutated and the mutations are identical, Hete

(heterozygous-variant) denotes a heterozygous variant, i.e., at least one of the alleles at this locus has been mutated and the alleles are different after the mutation.

Annovar Notes

ANNOVAR is a software that analyzes genetic variation using up-to-date data, giving a list containing: chromosome, starting position, ending position, nucleotide information on the reference sequence and nucleotide information detected. The distribution of SNPs/ InDel on several functional elements is statistically determined based on ANNOVAR analysis.

> Analysis results.

Annovar annotation table

Type	Count	Percent(%)
UTR3	2714	1.88
intronic	10593	7.36
downstream	4780	3.32
exonic;splicing	12	0.0
intergenic	9158	6.36
upstream	6551	4.55
splicing	325	0.22
UTR5	3566	2.47
upstream;downstream	1850	1.28
exonic	104295	72.5

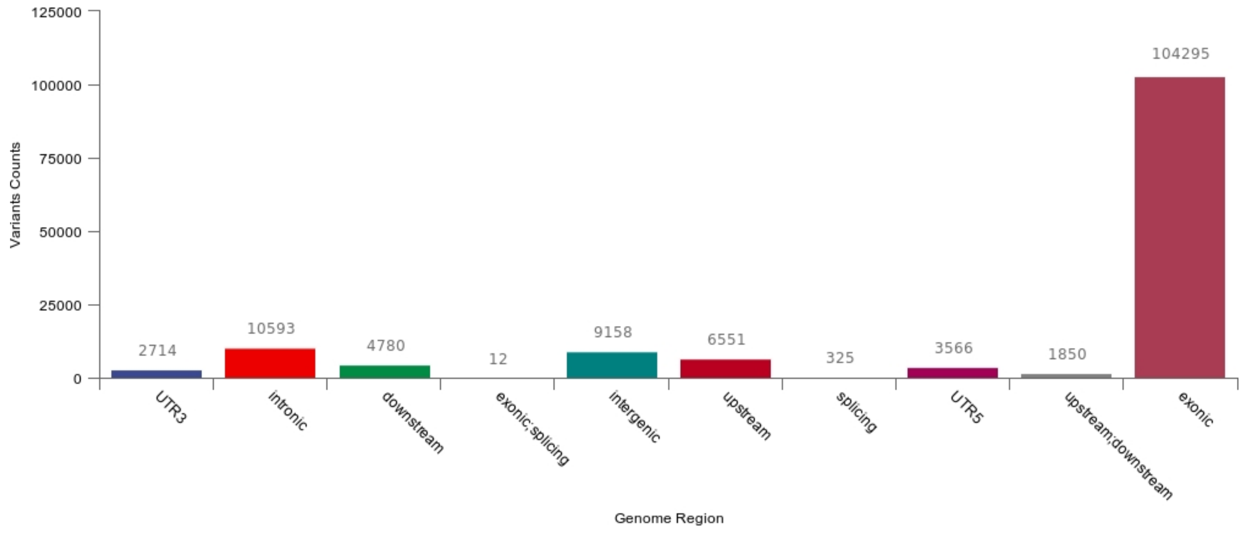

The horizontal coordinates are the regions annotated with SNP/InDel and the vertical coordinates are the number of SNP/InDel annotated to the different regions

Mutation type statistics

cSNPs are SNPs that occur in the coding region that directly affect the amino acid codons. cSNPs include conversion and subversion. SNP conversion: pyrimidines become pyrimidines or purines become purines, i.e., A and G are interchanged, and T and C are interchanged. cSNP subversion: pyrimidines are mutated to purines or vice versa, i.e., A and T are interchanged, A and C are interchanged, G and T are interchanged, and G and C are interchanged. The number of each type of conversion/subversion was counted separately;

> Analysis results.

Statistical table of mutation types

Genetype	Number
A/C	13568
A/G	38349
A/N	2
A/T	14196
C/G	11925
C/N	7
C/T	38287
G/N	1
G/T	13653
N/T	6

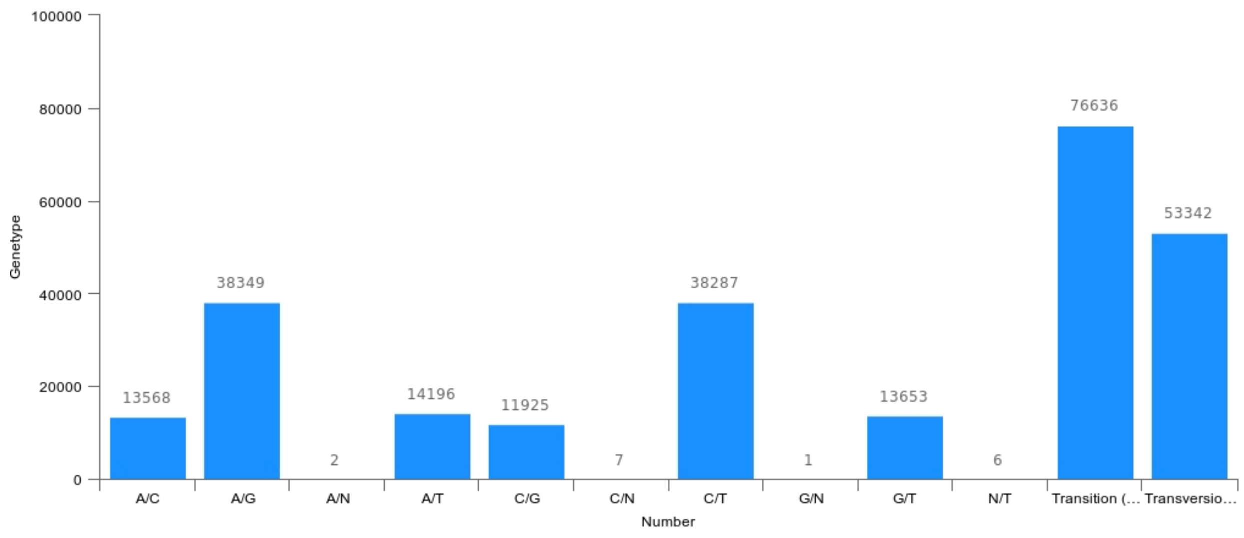

The horizontal coordinate is the mutation type and the vertical coordinate is the number of mutation types.

2.9 transcription factor analysis

Transcription factor family distribution

The process of eukaryotic transcription initiation is very complex and often requires the assistance of multiple protein factors. Transcription Factor (TF) is a class of protein molecules that can bind specifically to specific sequences upstream of the 5' end of a gene and form a transcription initiation complex with RNA polymerase II, which together participate in the process of transcription initiation. Transcription factors were predicted by comparing plants and animals separately with PlantTFDB (Plant Transcription FactorDatabase) and AnimalTFDB (Animal TranscriptionFactor DataBase) databases were compared to get the prediction of the transcription factors and the family information to which the transcription factors belong.

Analysis results.

Transcription Factor Family Distribution Table

Gene_ID	Symbol	Family
bnascaffold0022g0000200zs	bnascaffold0022g0000200zs	HD-ZIP
bnascaffold0025g0000500zs	bnascaffold0025g0000500zs	C2H2
Bnascaffold0025G0000700ZS	Bnascaffold0025G0000700ZS	HSF
bnascaffold0025g0003800zs	bnascaffold0025g0003800zs	ARF
Bnascaffold0025G0005300ZS	Bnascaffold0025G0005300ZS	bHLH

Bnascaffold0025G0009500ZS	Bnascaffold0025G0009500ZS	C2H2
Gene_ID	Symbol	Family
bnascaffold0025g0010300zs	bnascaffold0025g0010300zs	NAC
bnascaffold0025g0020100zs	bnascaffold0025g0020100zs	DBB
Bnascaffold0025G0021400ZS	Bnascaffold0025G0021400ZS	MYB_related
bnascaffold0025g0025400zs	bnascaffold0025g0025400zs	C2H2

GeneID: gene ID **Symbol**: gene name **Family**: family of transcription factors to which the gene belongs **Description**: gene description

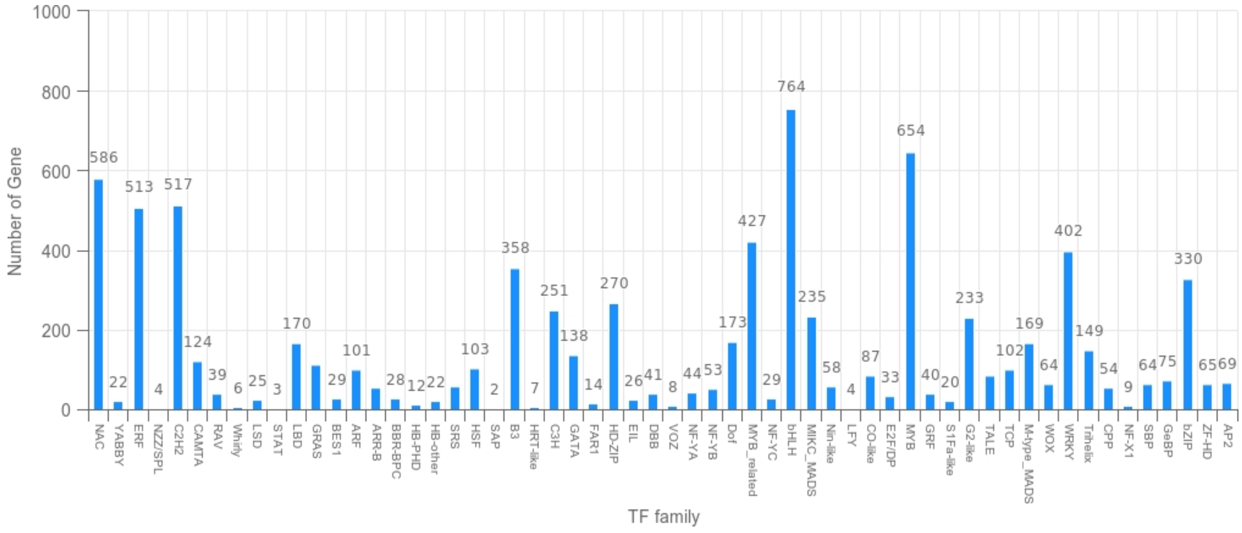

The horizontal coordinates are the different transcription factor families and the vertical coordinates are the number of genes that fall into that transcription factor family.

Distribution of differentially expressed transcription factors

The differentially expressed genes predicted to be transcription factors were counted, and the number of differentially expressed transcription factors contained in each transcription factor family in the comparison group was displayed in a bar graph based on the information of the family to which the transcription factors belonged.

Analysis results.

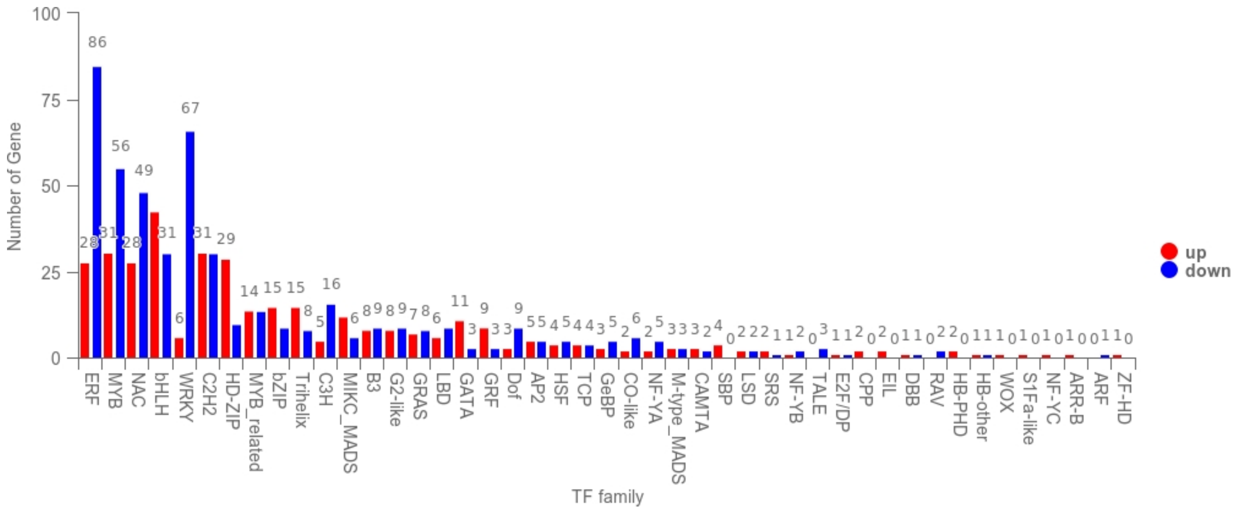

The horizontal coordinates are the different transcription factor families and the vertical coordinates are the number of differential genes that fall into that transcription factor family.

2.10 appendice

Introduction to the database

GO

A database established by the Gene Ontology Consortium (Gene Ontology, http://geneontology.org/).GO was created primarily to address the confusing nature of the definition of the same gene in different databases as well as the confusing nature of the functional definition of the same gene in different species. It is an internationally standardized classification system of gene functions, which provides a set of dynamically updated standard vocabulary (Controlled Vocabulary) to comprehensively describe the attributes of genes and gene products in living organisms.GO covers three aspects, which describe the molecular function of genes (Molecular Function), the role of cellular components (Cellular Component, and Biological Processes Involved.

(Biological Process.) Genes or proteins can be identified by ID correspondence or sequence annotation to find their corresponding GOs.

number, and the GO number can be used to correspond to a GO Term, i.e., functional class or cellular localization.

The basic unit of a GO is the Term, each of which has a unique identifier (consisting of "GO:" plus seven digits, e.g., GO: 0072669); the Terms of each class of Ontology form a directed acyclic topology by means of the links between them (is_a, part_of, regulate). form a directed acyclic topology.GOSlim is a reduced version of GO terminology, which provides an overview result of GO annotations.

KEGG

The Kyoto Encyclopedia of Genes and Genomes (KEGG, http:// www.kegg.jp/) is a database that integrates genomic, chemical, and systemic functional information. One of the features of the KEGG database is the association of gene catalogs derived from fully sequenced genomes with higher-level cellular, species, and ecosystem-level system functions.KEGG annotations include: (1) KO (KEGG Ortholog) annotations, which are cross-species annotations of information related to molecular networks; (2) KEGG Pathway annotations, which are metabolic pathway annotations, obtaining cross-species information; and (3) KEGG Pathway annotations, which are metabolic pathway annotations. (2) KEGG Pathway annotation, i.e. metabolic pathway annotation, to obtain the network of molecular interactions and reactions within a species.

UniProt

Subdatabases of the UniProt Knowledgebase (http://www.uniprot.org/help/uniprotkb) are high-quality, hand-annotated, non-redundant protein datasets that have been carefully verified by experienced molecular biologists and protein chemists. Each entry in the SwissProt database is annotated in detail, including structural domains, functional sites, transmembrane regions, disulfide bond positions, post-translational modifications, mutants, and more. The database also includes cross-reference codes with the nucleic acid sequence databases EMBL/GenBank/DDBJ, the protein structure database PDB, and more than a dozen secondary databases such as Prosite and PRINTTS.

EC

The Enzyme Commission of the International Society of Biochemistry

(http://enzyme.expasy.org/), classifies enzymes into six major groups based on the type and mechanism of the reactions they catalyze: oxidoreductases, transferase, hydrolases, cleavage enzymes, isomerases, and synthetases.

eggNOG

Evolutionary Genealogy of Genes: Non-supervised Orthologous Groups (http://eggnog.embl.de/version_3.0/) for eukaryotic direct homologous protein clustering, see (http:// www. ncbi.nlm.nih.gov/COG/). We will list the eggNOG IDs of all the genes, and then assign these eggNOG IDs to appropriate eggNOG classification units (Category), so as to classify the functions of all the genes in the genome, and to recognize the distribution of gene functions of the species from a macroscopic point of view.

The proteins that make up each eggNOG are hypothesized to be derived from an ancestral protein and are therefore either directly homologous to the

(Orthologs or paralogs are vertical lineages (objects) from different species.

(species formation) proteins that evolved and typically retain the same function as the original protein. paralogs are those proteins derived from gene duplication in a given species that may evolve new functions related to the original.

Introduction to the software used

hardware	functionality	parameters
Cutadapt	data filtering	At least 10 bp Overlap (AGATCGGAAG), allowing 20% of the
FastQC	quality control	default parameter
Tophat2	verify by comparing	Using microexon-search and library-type=fr-first
RSeQC	RPKM saturation analysis	default parameter
HTSeq	Expression quantification	Use of the union program
ggplot2	Mapping of volcanoes, MA	default parameter
Pheatmap	cluster analysis	default parameter
topGO	GO maps to DAG	default parameter
Sringtie	Quantification of assembled transcripts and LncRNA expression	default parameter
ASprofile	Variable shear event statistics	default parameter
Varscan	SNP and Indel detection	-p-value 0.01 --min-avg-qual 20
bowtie	nchor Reads Recompare	default parameter
DESeq	Variance analysis	log2foldchang|>1 and pvalue<0.05
Bowtie2	eads with transcript/gene sequence comparison	default parameter
Diamond	sequence comparison	-evalue 1e-5 -max_target_seqs 5
Blast2go	GO Note	default parameter

KAAS	KEGG Notes	Bi-directional Best Hit (BBH)

interpretation of nouns

Fuzzy bases / **N**: bases that cannot be identified in the sequencing, denoted by N. The more N in a sequence, the lower the quality of the sequence. The more N's in a sequence, the lower the quality of the sequence, and generally the sequence needs to be eliminated.

Adapter: An adapter is an artificial sequence that is added at each end of the sequence during sequencing. The adapter contains sequences that bind complementarily to the sequencing primers and sequences the target fragment by binding to the sequencing primers. When the sequence fragment after the addition of the adapter is shorter than the actual sequencing read length, the adapter sequence will be detected at the 3' end, and the adapter sequence needs to be removed before analysis.

Read count: the number of Reads matched to a gene.

Transcript: One or more mature mRNAs that can be used to code for proteins, formed by

the transcription of a gene. **Read / Reads**: Each sequence in the sequencing is called a Read

Raw Data / Raw Reads: Data after removal of connectors and low quality reads, subsequent analysis is based on Clean Data.

Clean Data / Clean Reads: the number of Reads compared to a gene.

K-mer: K-mer refers to a nucleotide sequence of length K obtained by successive cutting of a Read and base-by-base scribing.K-mer frequency statistical information is used to reveal the distribution pattern of various sub-sequences in a biological sequence, and it is an important tool for measuring sequence similarity.

ф (**exon inclusion level**): indicates the percentage of transcripts that include variable shear event regions among those that include and skip variable shear event regions

Chimera**:** A chimeric sequence formed by the absence of a splice or primer between two RoIs of the original sequence due to splice concentration, PCR primer concentration, or biological reasons.

Common Terminology

FASTQ format

The FASTQ format (http://en.wikipedia.org/wiki/FASTQ_format) is a text format commonly used for storing biological sequences and their corresponding quality scores. FASTQ format files can be opened by text editing software (e.g., WritePad, UltraEdit, EditPlus, etc.). FASTQ format files can be opened by text editing software (e.g., UltraEdit, EditPlus, etc.), which requires more memory on the computer due to the large size of the file.

The first row is the sequence name, starting with @, followed by the sequence description; the second row is the base sequence; the third row is the "+" sign, which doesn't mean anything; and the fourth row is the base mass, which corresponds to the base sequence in the second row. An example is shown below:

@M00200:111:000000000-A6VNV:1:1101:15594:1337 1:N:06

ACGCGGGGTATCTAATCCTGTTTGCTCCCCACGCTTTCGCGCCTCAGTGTCAGTTAC

+

ABABADBBDFFFGGGGFGGGFGGGHGBGHGGGGGGGGGGGGGGGGGGGGGGGGG

GGGGGGGGGGGGGGGGGGGGGGGGGGGGGGGGGGGGGGG

quality value

We used Sanger quality values to assess the sequencing quality of the downstream data. The quality value, or Q-value for short, is the result of a rounded mapping of the base read error rate p, which is equal to the Phred quality score, calculated as:

Qphred = -10 log10 p

Phred quality score formula

The concise correspondence between sequencing error rate and Q-value is shown in Table 2.

sequencing error rate	Q-value
5%	13
1%	20
0.1%	30
0.01%	40

Different sequencing platforms use different schemes to encode the quality of the bases in the FASTQ file. The correspondence between the Q value and the quality of the bases is as follows: the Q value plus an offset value, and the result obtained is converted into the corresponding characters according to the ASCII code value comparison table (see Table 3), and the reference information is shown below:

```
 !"#$%&'()*+,-./0123456789:;<=>?@ABCDEFGHIJKLMNOPQRSTUVWXYZ[\]^_`abcdefghijklmnopqrstuvwxyz{|}~
 |                         |    |        |                              |                     |
33                        59   64       73                            104                   126
 0........................26...31.......40
                          -5....0........9.............................40
                                0........9.............................40
                                   3.....9.............................40
 0.2......................26...31.........41

S - Sanger        Phred+33,  raw reads typically (0, 40)
X - Solexa        Solexa+64, raw reads typically (-5, 40)
I - Illumina 1.3+ Phred+64,  raw reads typically (0, 40)
J - Illumina 1.5+ Phred+64,  raw reads typically (3, 40)
    with 0=unused, 1=unused, 2=Read Segment Quality Control Indicator (bold)
    (Note: See discussion above).
L - Illumina 1.8+ Phred+33,  raw reads typically (0, 41)
```

Our FASTQ file is encoded in Illumina version 1.8+, and the Q-values of the bases are obtained by subtracting the offset value of 33 from the ASCII values of all characters. For example, if the ASCII value of character I is 73, and the offset value of 33 is subtracted to get 40, then the base quality of the corresponding position of this character is 40, and the sequencing error rate is 0.01%.

十进制	字符	Q值	十进制	字符	Q值	十进制	字符	Q值	十进制	字符	Q值
32			48	0	15	64	@	31	80	P	47
33	!	0	49	1	16	65	A	32	81	Q	48
34	“	1	50	2	17	66	B	33	82	R	49
35	#	2	51	3	18	67	C	34	83	S	50
36	$	3	52	4	19	68	D	35	84	T	51
37	%	4	53	5	20	69	E	36	85	U	52
38	&	5	54	6	21	70	F	37	86	V	53
39	’	6	55	7	22	71	G	38	87	W	54
40	(	7	56	8	23	72	H	39	88	X	55
41	)	8	57	9	24	73	I	40	89	Y	56
42	*	9	58	:	25	74	J	41	90	Z	57
43	+	10	59	:	26	75	K	42	91	[	58
44	,	11	60	<	27	76	L	43	92		59
45	-	12	61	=	28	77	M	44	93	]	60
46	.	13	62	>	29	78	N	45	94	^	61
47	/	14	63	?	30	79	O	46	95	_	62

quartile (math.)

Quartiles are all values arranged from smallest to largest and divided into four equal parts, with the values at the first and third division points being the quartiles.

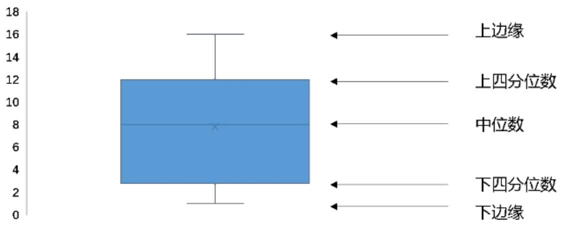

Sam / Bam format

Sam (sequence alignment/map format) is a sequence alignment format standard developed by Sanger, with Tab as the separator of the text format, which can be opened by text editing software (such as WritePad, UltraEdit, EditPlus, etc.), and is mainly used for the representation of the results of sequencing sequences aligned to the genome, of course, any multiple alignment results can be represented. Of course, it can also be used to represent arbitrary multiple alignment results. After aligning a fastq file to a genome, we usually get a file with the extension Sam or Bam. Bam is a binary file of Sam (B is derived from binary), which takes up less space and cannot be opened, but can only be converted to Sam format by software such as samtools.

Sam is divided into two sections, header section and alignment section. Each line starts with @ and different tags are used to indicate different information. The tags include @HD (standardized version, description of the alignment of the comparison sequence), @SQ (description of the reference sequence), @RG (description of the sequences on the comparison), @PG (description of the program used), @CO (arbitrary description information). Each line of the comparison result section represents the comparison

information of a segment, including 11 mandatory fields and one optional field, which is separated by Tab. Examples and descriptions are given below:

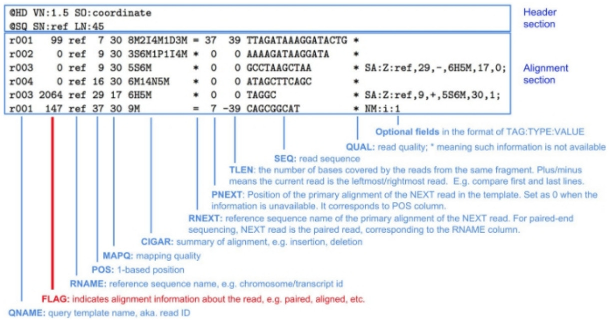

1. QNAME, the number of the comparison segment

Sam is divided into two sections, header section and alignment section. Each line starts with @ and different tags are used to indicate different information. The tags include @HD (standardized version, description of the alignment of the comparison sequence), @SQ (description of the reference sequence), @RG (description of the sequences on the comparison), @PG (description of the program used), @CO (arbitrary description information). Each line of the comparison result section represents the comparison information of a segment, including 11 mandatory fields and one optional field, which is separated by Tab. Examples and descriptions are given below:

2. FLAG, bit identifier, 1 means that the read is one of the pair (read means this read, mate means another read in the pair), 2 means that the pair is positively and negatively compared to the reference sequence, 4 means that this read is not compared to the reference sequence, 8 means that the mate is not compared to the reference sequence, 16 means that this read is compared to the negative chain, 32 means that the mate is compared to the negative chain, 64 means that this read is read1, 128 means that this read is read2 and so on. The value of FLAG is the sum of the numbers that match the situation, i.e., 83= (64+16+2+1) means that the read is read1, which is compared to the negative chain, and its mate is compared to the positive chain.

3. RNAME, reference sequence number

4. POS, the position of the match, note that the counting starts from 1, if there is no match, it is 0 here.

5. MAPQ, the quality of the comparison, the higher it is the more unique the locus is, calculated as Q = -10 log 10p, p is the estimate that the sequence does not come from this locus

6. CIGAR (Compact Idiosyncratic Gapped Alignment Report), using numbers and letters to indicate the results, such as M for match/mismatch, I for insertion, D for deletion, etc., and the numbers indicate the number of bases, i.e. 42M4I5M for the sequence 42 base matches, 4 insertions, 5 base matches.

7. RNEXT, the name of the mate, denoted by * if no mate is available

8. PNEXT, the position of the mate, if there is no mate, it is denoted by 0

9. TLEN, the distance between paired reads, which is negative when the mate sequence is upstream of this sequence, or 0 if the comparison region has only one segment, or is unavailable

10. SEQ, read sequence

11. QUAL, read quality

12. Optional Fields, optional fields, format such as: TAG:TYPE:VALUE, where TAG consists of two uppercase letters, each TAG represents a kind of information, such as AS means match score, XS means second best match score, YS means mate sequence match score, etc., TYPE means the type of the value corresponding to the TAG, which can be a string (TYPE represents the type of the value corresponding to the TAG, which can be a string (Z), integer (i), etc.

RPKM / FPKM

The amount of data obtained after filtering is unlikely to be identical across samples, and the length of different genes varies greatly. In order to be able to compare gene expression within samples (different genes) and between samples (different groups), expression normalization using FPKM is required.

(Normalization).

FPKM (Fragments Per Kilobase Million), the number of Reads per Kilobase Length from a gene per Million Reads, is a commonly adopted method of gene expression normalization that takes into account both the effects of sequencing depth and gene length on gene expression counts. The formula is shown below:

$$\text{FPKM} = \frac{total\ exon\ reads}{mapped\ reads\ (millions) * exon\ length\ (KB)}$$

Currently, the FPKM-based expression normalization method is gradually replaced by other statistical methods, but as an absolute normalization method, its biological significance is clear, which facilitates the comparison between different projects. In the reference transcriptome, we generally consider that genes with FPKM>1 are expressed. This threshold is recommended by mainstream journals, and can also reflect the gene expression level well.

In addition, the RPKM value can also be used to characterize the amount of gene expression, and the calculation methods of FPKM and RPKM are basically the same, with the following difference: for Pair-End sequencing, there are two Reads for each Fragment, and FPKM only calculates the number of Fragments that can be matched to the same transcript with two Reads, whereas RPKM calculates the number of Reads that can be matched to the same transcript with two Reads, and RPKM calculates the number of Reads that can be matched to the same transcript with one Read. RPKM calculates the number of Reads that can be matched to a transcript.

GFF /GTF format

The gff format is a data format defined by the Sanger Institute to easily characterize DNA, RNA, and protein sequences. It has become a common format for sequence annotation, and many software programs support importing or exporting the gff format. Each row represents a feature entry (e.g., gene, transcript, CDS, exon, etc.), and each row has 9 columns separated by a tab, with each column listing some information about the feature entry. gff can be opened by text editing software (e.g. WritePad, UltraEdit, EditPlus, etc.). The latest version of gff is version 3, an example of which is shown below:

ctg123	PFAM gene	1000 5000 . + . ID=gene001;Name=EDEN
ctg123	PFAM TF_binding_site	1000 1012 . + . Parent=gene001
ctg123	PFAM mRNA	1050 5000 . + . ID=mRNA001;Parent=gene001
ctg123	PFAM mRNA	1050 5000 . + . ID=mRNA002;Parent=gene001
ctg123	PFAM exon	1300 1500 . + . Parent=mRNA001

ctg123	PFAM exon	1050 1500 . + . Parent=mRNA001,mRNA002
ctg123	PFAM CDS	1201 3902 . + 0 ID=cds001;Parent=mRNA001
ctg123	PFAM CDS	3000 4600 . + 2 ID=cds001;Parent=mRNA001
ctg123	PFAM CDS	1201 1500 . + 1 ID=cds002;Parent=mRNA002

1. Sequence number, may be the name of a chromosome or scaffold

2. Source, program, database or project that generated the feature entry

3. Types such as gene, transcript, CDS, mRNA, exon, five/three_prime_utr, start/stop_codon, etc.

4. Start site, the starting position of this feature entry on the sequence, counting from 1

5. The termination site, where this feature entry terminates on the sequence, cannot be larger than the length of the sequence

6. The score, which is an indication of the likelihood of the annotation information, can be an E-values value for sequence similarity comparison or a P-values value for gene prediction. "." indicates that it is empty

7. The direction of the sequence, + denotes the justice chain, - the antisense chain , ? denotes unknown

8. Phase, valid only for entries of type "CDS", valid values are 0, 1, 2, 0 means that the first base of this feature entry is the first base of a codon, 1 means that the second base of this feature entry is the first base of a codon, and so on

9. attribute, described by annotation information consisting of multiple key-value pairs, with "=" between keys and values, different key-value pairs separated by ";", a key can have multiple values, and different values are separated by ",". ". A key can have multiple values, and different values are separated by ",". The key can be ID (the number of the feature entry, which must be unique in a gff file), Name (the name of the feature entry, which can be repeated), Parent (the parent of the feature entry, and the value is the number of the parent feature entry, such as the number of the transcript to which the exon belongs, the number of the gene to which the transcript belongs, etc), etc. The value can be more than one. The value can be more than one), etc.

The gtf format is basically the same as the first 8 columns of the gff format, but the difference is in the 9th column. Although it is the same case of tag-value pairing, the gtf format separates the tags from the values by a space, and each attribute must be followed by a semicolon; (including the last attribute), and the 9th column must start with gene_id and transcript_id.

Variable shear events

Variable splicing (or selective splicing) refers to the generation of different mRNA splicing isoforms from an mRNA precursor of some genes by different splicing methods (selection of different splice sites). It is generally believed that there are five basic forms of variable splicing: (1) intron retention; ② variable 5' end; (3) variable 3' end; (4) skipping exons; and (5) mutually exclusive exons (only one of a group of exons can be expressed). There are also categorized into seven forms
style, i.e., the above five variable shear forms plus variable first or last exon, while these two forms are more likely to be caused by variable promoter, variable polyA site. As shown in the figure below:

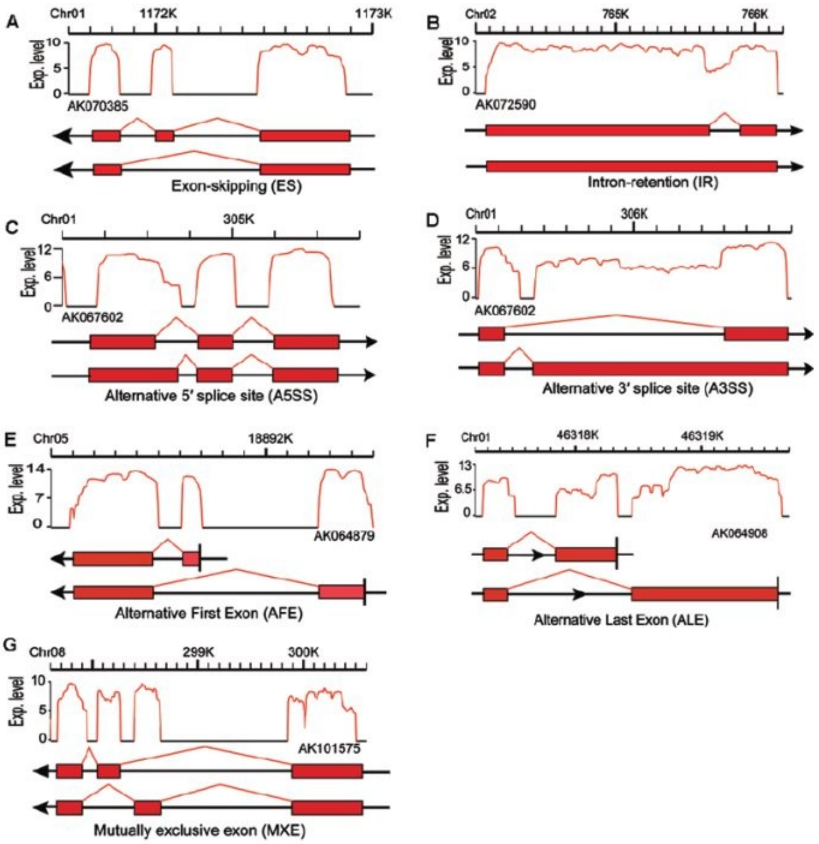

The ASprofile software then categorizes variable clipping events into 12 categories:

1) TSS: Alternative 5' first exon, variable first exon 2)

TTS: Alternative 3' last exon, variable last exon 3) SKIP:

Skipped exon, skipped single exon

4) XSKIP: Approximate SKIP, skipping single exons (fuzzy boundaries)

5) MSKIP: Multi-exon SKIP, skipping multiple exons

6) XMSKIP: Approximate MSKIP, skipping multiple exons

(fuzzy boundaries) 7) IR: Intron retention, single intron

retention

8) XIR: Approximate IR, single intron retention

(fuzzy boundaries) 9) MIR: Multi-IR, multiple intron

retention

10) XMIR: Approximate MIR, multiple introns retained

(fuzzy boundaries) 11) AE: Alternative exon ends, 5' or 3'

ends of exon variable 12) XAE: Approximate AE, 5' or 3'

ends of exon variable (fuzzy boundaries) **FASTA** Formats

In bioinformatics, the FASTA format (also known as Pearson format), is a text-based format for representing nucleotide sequences or amino acid sequences, which can be opened by text editing software (e.g., WritePad, UltraEdit, EditPlus, etc.). The first line of the sequence file is an arbitrary text description beginning with a greater-than sign ">" or a semicolon ";" (it is customary to use ">" as the start), which is used for sequence labeling. Starting on the second line is the sequence itself, and only established nucleotide or amino acid coding symbols are allowed. Nucleotide symbols are usually case-sensitive, while amino acids are often written in capital letters. The file should normally have no more than 80 characters of letters per line. Examples are as follows: (only one expression in a set of exons is allowed). There are also categorized into 7 forms, i.e., the above 5 variable shear forms plus variable first or last exon, while these two forms are more likely to be caused by variable promoters, variable polyA sites. The figure below:

>Seq1

ADQLTEEQIAEFKEAFSLFDKDGDGTITTKELGTVMRSLGQNPTEAELQDMINEVDAD*

Class Code

Class Code is a description given by Stringtie of the position of the spliced transcript in relation to known genes and transcripts.

prioritization	Code	descriptive
1	=	Intron chain exact match
2	c	incorporate
3	j	Potential new transcripts: at least one variable shear site shared with known transcripts
4	e	An exonic fragment covering a known exon and a known intron of at least 1[illegible] bp, probably an mRNA precurso[illegible]
5	i	The transcript fragment is entirely in a known intron
6	o	Essentially covers a known transcript at the exon level
7	p	Possibly polymerase-generated fragments (contained within 2k bases of a known transcript)
8	r	Repeat fragments when the reference genomic sequence has soft-masked bases and 50% of the bases in the transcript are lowercase
9	u	Unknown fragment, transcript in intergenic region
10	x	Exons cover the reverse strand of known genes

11	s	An intron of the transcript covers the reverse strand of a known intron, possibly due to a comparison error
12		Includes a wide range of scenarios

common problems

Q: What are the static and images folders in the results file for?

A: static folder is the static files required for the web page format, does not contain the results of the content, please do not change the files under the static folder.
Otherwise, the content of the web report will be affected. In the images folder, there are all the pictures in png format, and their analyzed contents and corresponding pdfs are stated in the "Result Files" under each section, so if you modify or move the images folder and its contents, it will also affect the display of the webpage report.

Q: Which is more reliable when the clustering results from PCA analysis, sample correlation test, and cluster analysis heatmap are different?

A: PCA analysis and sample relevance test are both based on the expression of all genes in the sample, while clustering heatmap is based on the expression of differential genes for two-way clustering of samples and genes, and the two are targeting different problems. If the clustering results of PCA and sample correlation test are different, the PCA clustering results will prevail, because the PCA analysis will keep the information of genes contributing to the samples, while the sample correlation test will treat all the genes in the same way, and the PCA clustering results are more accurate.

Q: Why does Parsonage use the p value in the variance analysis and not the corrected p value for screening?

A: Because the p value calculation method of DESeq2 is already very strict, using the p value is sufficient as a screening criterion, and fewer differential genes/LncRNAs/CircRNAs may be screened out, or even none, if the corrected p value is used for screening. If the number of differential genes/LncRNAs/CircRNAs screened is high, the p value can be adjusted to reduce the number of differential genes screened.

Q: Can the screening conditions be modified to obtain different numbers of differential genes / LncRNA / CircRNA?

A: Yes, differential expression is a relative concept, and the desired number of differential genes / LncRNAs / CircRNAs can be obtained by modifying the screening conditions, although pvalue < 0.05 and |log2FoldChange|>1 are generally recommended.

Q: Is it possible to do differential analysis using only some genes / LncRNA / CircRNA?

A: No. Differential analysis is done based on all genes/LncRNA/CircRNA as background. If some genes/LncRNA/CircRNA are used for differential analysis, the overall information, such as sequencing depth, reads distribution characteristics, etc., will be lost, resulting in bias.

Q: Why are there genes / LncRNAs / CircRNAs that are significantly different in expression in two samples, but are not significantly different genes / LncRNA / CircRNA ?

A: Because significant difference is a statistically based concept, it is not possible to intuitively determine whether a gene / LncRNA / CircRNA is significantly different by the magnitude of its expression, but rather, it needs to be judged after calculation on an

overall basis.

Q: Can a cluster analysis heat map adjust the order of genes / LncRNA / CircRNA or samples?

A: No, the cluster analysis heatmap is formed by automatic clustering based on expression, similar genes / LncRNAs / CircRNAs or samples are clustered together and the result represents the distance between samples or genes / LncRNAs / CircRNAs.

Q: What is the difference between GO functional enrichment and GO functional classification?

A: GO Function Classification is to annotate genes under the GO classification of the corresponding function, while GO Function Enrichment Analysis is to enrich the set of functionally similar genes together by a statistical test algorithm, thus facilitating the study of genes with a certain type of function.

Q: Why does Paisano only provide links to metabolic pathway maps in the results of differential gene KEGG enrichment analysis instead of providing metabolic pathway maps directly?

A: Firstly, the image format will take up a lot of space; secondly, the KEGG webpage linked to will provide richer content. Hovering over a node will bring up information such as the node number, clicking on the node will jump to the node's detailed information, and you can also select the species to view the metabolic pathways specific to the corresponding species, and if you want to save the metabolic pathway diagram, you can right click on the image to save it.

bibliography

[1] Tatusov RL, Fedorova ND, Jackson JD, Jacobs AR, Kiryutin B, Koonin EV, Krylov DM, Mazumder R, Mekhedov SL, Nikolskaya AN, Rao BS, Smirnov S, Sverdlov AV. Vasudevan S, Wolf YI, Yin JJ, Natale DA. The COG database: an updated version includes eukaryotes[J]. BMC Bioinformatics. 2003 Sep 11,4:41.

[2] Powell S, Szklarczyk D, Trachana K, Roth A, Kuhn M, Muller J, Arnold R, Rattei T, Letunic I, Doerks T, Jensen LJ, von Mering C, Bork P. eggNOG v3.0. Orthologous groups covering 1133 organisms at 41 different taxonomic ranges[J]. Nucleic Acids Res. Epub 2011 Nov 16; PubMed 22096231.

[3] The Gene Ontology Consortium, Michael Ashburner, Catherine A. Ball, Judith A. Blake, David Botstein, Heather Butler, J. Michael Cherry, Allan P. Davis, Kara Dolinski, Selina S. Dwight, Janan T. Eppig, Midori A. Harris, David P. Hill, Laurie Issel-Tarver, Andrew Kasarskis, Suzanna Lewis, John C. Matese, Joel E. Richardson, Martin Ringwald, Gerald M. Rubin, and Gavin Sherlock. gene ontology: tool for the unification of biology[J]. Nat Genet. 2000 May, 25(1): 25-29.

[4] Minoru Kanehisa,* Susumu Goto, Shuichi Kawashima, Yasushi Okuno, and Masahiro Hattori. The KEGG resource for deciphering the genome[J]. Nucleic Acids Res. 2004
January 1; 32 (Database issue): D277-D280.

[5] Zhou L., Chen J., Li Z., Li X., Hu X., et al. (2010). Integrated profiling of microRNAs and mRNAs: microRNAs located on Xq27.3 associate with clear cell renal cell carcinoma. PLoS One 5: e15224.

[6] Michael I Love,Wolfgang Huber,Simon Anders.(2014).Moderated estimation of fold change and dispersion for RNA-seq data with DESeq2.Genome Biology.

[7] Wang L., Feng Z., Wang X., Wang X., Zhang X. (2010). DEGseq: an R package for identifying differentially expressed genes from RNA-seq data. Bioinformatics 26, 136-8.

[8] Kanehisa M, Araki M, Goto S, Hattori M, Hirakawa M, et al. (2008). KEGG for linking genomes to life and the environment. Nucleic Acids researpartA_ch16:D480-484.

[9] Trapnell C, Hendrickson DG, Sauvageau M, Goff L, Rinn JL, Pachter L: Differential analysis of gene regulation at transcript resolution with RNA-seq. Nature

biotechnology 2013, 31(1):46-53.

[10] Gotz S, Garcia-Gomez JM, Terol J, Williams TD, Nagaraj SH, Nueda MJ, Robles M, Talon M, Dopazo J, Conesa A: High-throughput functional annotation and data mining with the Blast2GO suite. nucleic acids research 2008, 36(10):3420-3435.

[11] Moriya Y, Itoh M, Okuda S, Yoshizawa AC, Kanehisa M: KAAS: an automatic genome annotation and pathway reconstruction server. nucleic acids research 2007 , 35(Web Server issue):W182-185.

[12] Rogers MF, Thomas J, Reddy AS, Ben-Hur A: SpliceGrapher: detecting patterns of alternative splicing from RNA-Seq data in the context of gene models and EST data. Genome Biol. 2012, 13(1):R4.
