# Supplementary material for: Targeted Metabolites and Transcriptome Analysis Uncover the Putative Role of Auxin in Floral Sex Determination in Litchi chinensis Sonn
Source: Plants (Basel). 2024 Sep 16;13(18):2592. doi: 10.3390/plants13182592 (PMC11435090; doi:10.3390/plants13182592)
Supplement: Supplementary file 1 [file plants-13-02592-s001.zip › Supplementary Figures.pdf]

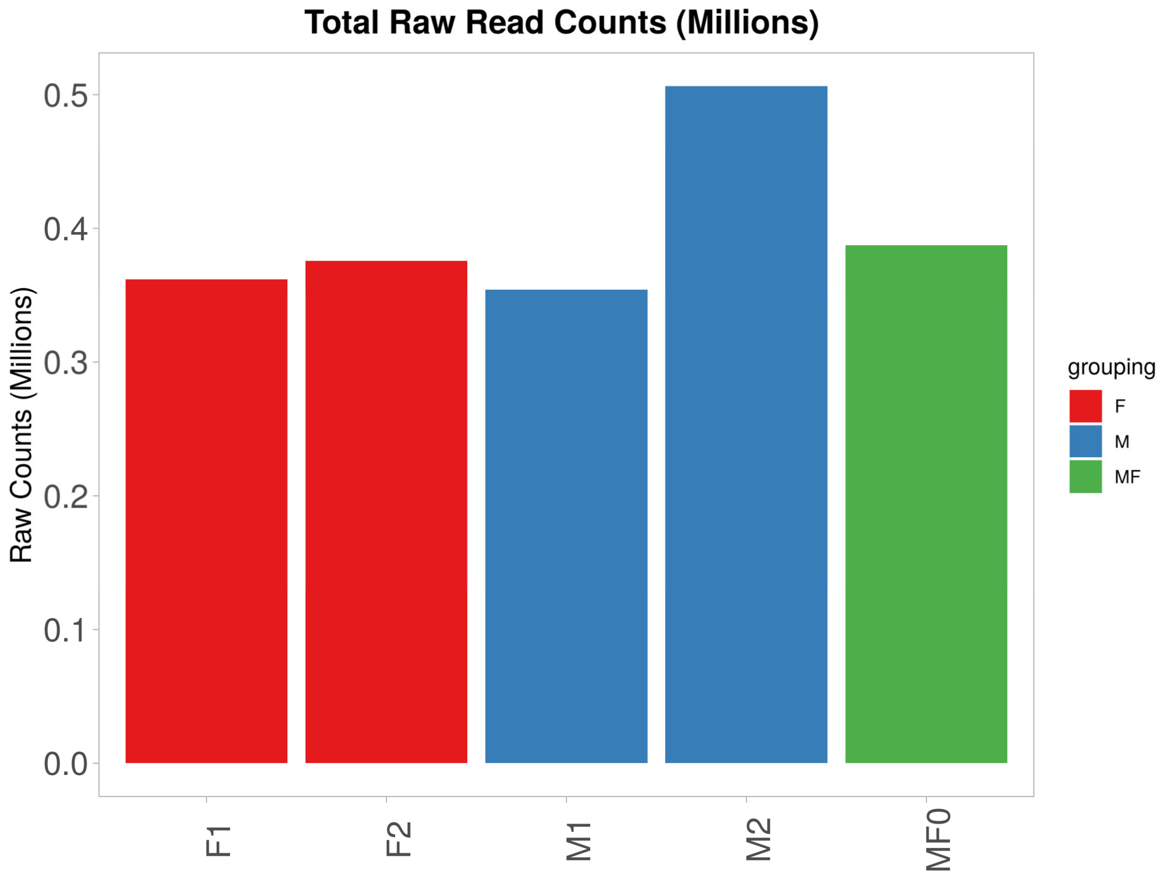

Figure S1: Total raw read counts found from the litchi transcriptome dataset.

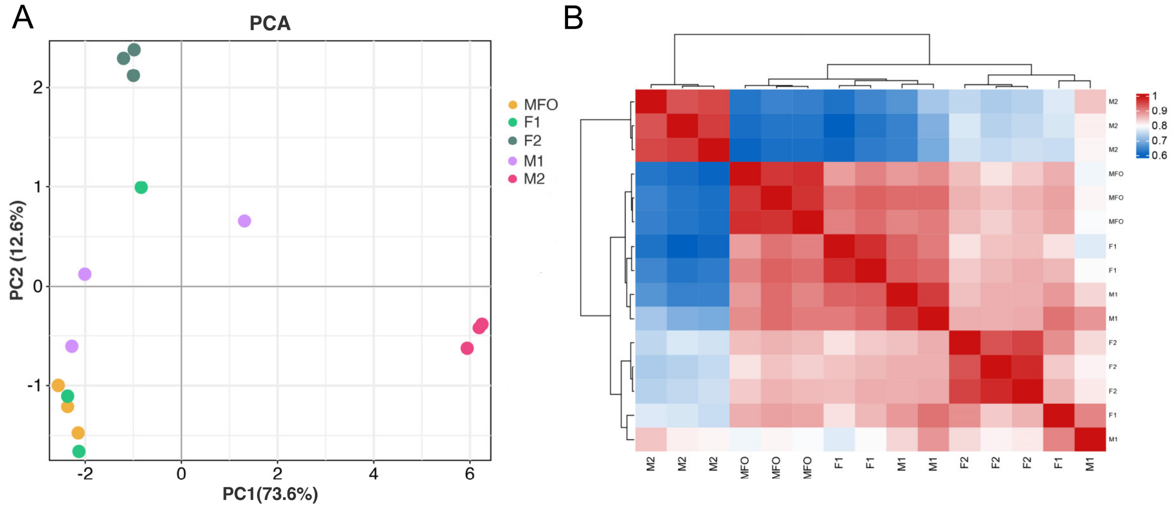

Figure S2: PCA and correlation analysis of sampling data during floral bud development.
